# A Clinician-Configurable FAHP-Based Ensemble Framework for Multi-Label Chest X-Ray Classification

**DOI:** 10.3390/diagnostics16193132

**Published:** 2026-09-26

**Authors:** Hui-Chu Chiu, Yu-Hsiang Tsai, Cheng-Hsuan Juan, Chia-Ching Chang, Ya-Hui Li, Deng-Yiu Chiu, Chun-Jung Juan

**Affiliations:** 1 Ph.D. Program of Management, Chung-Hua University, Hsinchu 300, Taiwan; 2 Department of Medical Imaging, China Medical University Hsinchu Hospital, Hsinchu 302, Taiwan; 3 Department of Medical Imaging, China Medical University Hospital, Taichung 404, Taiwan; 4 Graduate Institute of Biomedical Electronics and Bioinformatics, National Taiwan University, Taipei 106, Taiwan; 5 Department of Management Science, National Yang Ming Chiao Tung University, Hsinchu 300, Taiwan; 6 Department of Information Management, Chung-Hua University, Hsinchu 300, Taiwan; 7 Department of Radiology, School of Medicine, College of Medicine, China Medical University, Taichung 406, Taiwan; 8 Department of Biomedical Engineering and Environmental Sciences, National Tsing Hua University, Hsinchu 300, Taiwan; 9 Department of Computer Science and Information Engineering, National Taiwan University, Taipei 106, Taiwan

**Keywords:** chest X-ray, deep learning, ensemble learning, fuzzy analytic hierarchy process, clinical decision support, model interpretability

## Abstract

**Background/Objectives:** Deep learning-based chest X-ray (CXR) analysis has shown strong diagnostic performance; however, conventional ensemble methods typically rely on equal-weight aggregation and do not account for disease-specific model behavior or clinician-defined priorities. **Methods:** We developed a clinician-configurable ensemble framework based on the fuzzy analytic hierarchy process (FAHP) for multi-label CXR classification. The proposed method employs a two-stage design consisting of disease-specific model weighting using multi-criteria decision analysis and utility-guided seed aggregation based on ΔNet Benefit (ΔNB). Five base architectures, including Vision Transformer (ViT), SwinV2 Transformer, DenseNet121, DenseNet121 + ViT and DenseNet121 + SwinV2, were trained on the National Institutes of Health (NIH) ChestX-ray14 dataset using 10 random seeds. Performance was evaluated on a held-out test set using area under the receiver operating characteristic curve (AUROC), area under the precision–recall curve (AUPRC), balanced accuracy, and F1 score and compared with single-model baselines, Hard Voting, and Soft Voting. **Results:** In the seed-level analyses, Soft Voting achieved the highest overall discrimination performance (AUROC, 0.851 ± 0.001; AUPRC, 0.293 ± 0.003), whereas seed-level FAHP showed comparable performance (AUROC, 0.850 ± 0.001; AUPRC, 0.287 ± 0.004). In the final-level analyses, the sensitivity-priority FAHP–ΔNB configuration achieved a macro-averaged AUROC of 0.855 and an AUPRC of 0.299. Hard Voting demonstrated inferior performance, particularly for AUPRC. Per-disease analysis revealed heterogeneous results, with FAHP showing favorable performance in selected conditions, including Effusion, Emphysema, Mass, Nodule, and Pleural Thickening, while Soft Voting remained superior in others. Statistical analysis confirmed that differences between FAHP and Soft Voting were generally modest and disease-dependent. **Conclusions:** The proposed FAHP-based ensemble maintains performance comparable to strong equal-weight baselines while enabling clinician-configurable, disease-specific model weighting and utility-guided aggregation. This framework provides an aggregation-level transparent approach for incorporating configurable diagnostic priorities into multi-label CXR classification.

## 1. Introduction

Deep learning (DL) has demonstrated strong performance in automated chest X-ray (CXR) analysis, with convolutional neural networks (CNNs) and transformer-based architectures achieving promising results across multiple thoracic diseases [1,2,3]. To further improve robustness and generalization, ensemble learning techniques have been widely adopted [4]. Among them, Hard Voting and Soft Voting are commonly used due to their simplicity and effectiveness. However, these conventional ensemble approaches rely on equal-weight aggregation, implicitly assuming that all models contribute equally across diseases and performance criteria.

This assumption represents an important limitation. In practice, different model architectures exhibit heterogeneous performance profiles, often involving trade-offs among sensitivity, specificity, and precision across diseases. Moreover, the relative importance of these metrics is inherently context-dependent in clinical settings. For example, sensitivity is typically prioritized in screening scenarios to reduce missed diagnoses, whereas specificity may be more critical in confirmatory or resource-limited settings [5]. Despite these considerations, most existing ensemble frameworks do not incorporate clinician preferences or disease-specific model behavior into the aggregation process. In addition, variability arising from stochastic training processes (e.g., different random seeds) is typically handled using equal-weight averaging, without considering the relative utility of individual model instances.

Accordingly, a key research gap exists in the development of ensemble methods that are simultaneously performance-aware, disease-specific, and clinically configurable. Specifically, there remains a lack of frameworks that (1) incorporate multi-criteria diagnostic performance into model weighting, (2) allow for the explicit integration of clinician-defined priorities, and (3) account for seed-level variability using decision-relevant utility measures [6].

Our previous work focused on the design and evaluation of hybrid CNN–transformer architectures and class-wise ensemble fusion strategies for multi-label CXR classification, including external validation across multiple datasets [7]. In contrast, the present study addresses a fundamentally different research objective centered on ensemble-level decision modeling rather than model architecture design. Specifically, instead of proposing new backbone architectures or feature fusion mechanisms, this work investigates whether a clinician-configurable, multi-criteria ensemble framework can improve the interpretability and adaptability of model aggregation. Furthermore, the current study employs a distinct set of base models, including Vision Transformer (ViT), SwinV2 Transformer architectures [8,9], and parallel DenseNet121–transformer hybrids, which were not used in the previous study. All base models were independently trained within the present experimental pipeline using ImageNet-pretrained initialization, and all validation procedures, threshold derivation, fuzzy analytic hierarchy process (FAHP) weighting, ΔNet Benefit (ΔNB)-guided seed aggregation, and test-set predictions were newly generated, with no reuse of model weights, checkpoints, predictions, or experimental results from our previous study.

We hypothesize that an ensemble framework integrating clinician-defined preferences and validation-based utility signals can achieve performance comparable to conventional equal-weight methods while improving interpretability and enabling disease-specific adaptation. In particular, by weighting models according to sensitivity-, specificity-, and precision-based criteria and incorporating validation-derived ΔNB-guided aggregation across independently trained seed-level ensemble members, the ensemble can better align with configurable diagnostic priorities without sacrificing overall discrimination performance.

To test this hypothesis, we developed a clinician-configurable ensemble framework based on the FAHP [10,11] for multi-label CXR classification. The proposed framework integrates disease-specific model weighting using multi-criteria decision analysis with validation-derived ΔNB-guided seed aggregation across random seeds. The purpose of this study was to evaluate whether the proposed FAHP-based ensemble can provide comparable or improved diagnostic performance relative to conventional ensemble methods while offering enhanced configurability and interpretability in multi-label CXR analysis.

## 2. Materials and Methods

This retrospective study utilized the publicly available National Institutes of Health (NIH) ChestX-ray14 dataset [12]. All data were fully de-identified and publicly accessible, and no identifiable personal information was available to the authors. The study protocol was reviewed by the China Medical University & Hospital Research Ethics Center and was determined to be exempt from ethical review. The requirement for informed consent was also waived.

The dataset selection and preparation workflow is illustrated in Figure 1. The dataset comprised 112,120 frontal-view CXRs from 30,805 patients. It was partitioned on a patient-wise basis into training, validation, and internal test subsets. The final split included 78,468 images from 21,528 patients for training, 11,219 images from 3090 patients for validation, and 22,433 images from 6187 patients for internal testing. All partitions were defined at the patient level, and all CXRs belonging to the same patient were restricted to a single subset. A formal leakage audit confirmed zero image overlap and zero patient overlap among the training, validation, and internal test subsets. The complete patient-wise dataset partition and leakage audit are provided in Supplementary Table S1.

The dataset was annotated for 14 thoracic disease categories in a multi-label classification setting, including Atelectasis, Cardiomegaly, Consolidation, Edema, Effusion, Emphysema, Fibrosis, Hernia, Infiltration, Mass, Nodule, Pleural Thickening, Pneumonia, and Pneumothorax. Each image may contain one or more co-existing disease labels.

### 2.1. Study Overview

We developed and evaluated a clinician-configurable ensemble framework for multi-label CXR classification using five base architectures and 10 independent random seeds per architecture. The framework was designed to address two distinct sources of variability: inter-architecture heterogeneity and seed-to-seed stochastic variation. Under this design, Soft Voting and Hard Voting served as equal-weight baselines, whereas the proposed FAHP ensemble was formulated as a two-stage preference-aware ensemble incorporating disease-specific model weighting and utility-guided seed aggregation (Figure 2). In stage 1, disease-specific model weights are derived using the FAHP based on sensitivity, specificity, and precision. In stage 2, seed-level predictions are aggregated using validation-derived ΔNB to incorporate decision-relevant utility. The primary proposed method in this study is therefore FAHP–ΔNB, whereas seed-level FAHP analyses are presented as an ablation of the disease-specific model-weighting stage. The primary FAHP configuration was defined as an illustrative sensitivity-priority scenario designed to reflect a plausible screening-oriented clinical preference. Formal preference elicitation from a clinician panel was not performed in this study. To empirically examine the configurability of the framework, additional specificity-priority and balanced FAHP configurations were evaluated in supplementary analyses while retaining the same trained checkpoints, validation and test predictions, γ parameter, ΔNB weighting procedure, and evaluation datasets. Therefore, the term clinician-configurable refers to the framework’s ability to incorporate user-defined or clinician-defined priorities rather than to a preference model empirically elicited from clinicians in the present study. Constrained weight optimization (CWO) was evaluated separately as a conventional validation-based weighted-ensemble comparator at the final aggregation level.

### 2.2. Base Models and Training

Five base architectures were used: ViT [8], SwinV2 Transformer (SwinV2) [9], DenseNet121 [13], a parallel DenseNet–ViT hybrid, and a parallel DenseNet–SwinV2 hybrid (DenseNet121 + SwinV2). All models were trained on the NIH ChestX-ray14 dataset using the predefined patient-wise data split described above. To assess robustness to random-seed variation, each architecture was trained using 10 different random seeds, generating 10 seed-level ensemble members per architecture. For each retained checkpoint, validation-set and test-set probabilities were exported and used for thresholding and ensemble analyses.

To ensure reproducibility, all models were trained using a standardized configuration. Input images were resized to 224 × 224 pixels. Training augmentation included RandomResizedCrop with a scale range of 0.9–1.0 and horizontal flipping, followed by ImageNet normalization. ImageNet normalization was applied as an input preprocessing step, and all backbone architectures were initialized using ImageNet-pretrained weights. The batch size was 32. To address class imbalance, all models were trained using positive-class-weighted binary cross-entropy implemented with the pos_weight argument of BCEWithLogitsLoss using PyTorch (2.7.1+cu126). For each disease label, the positive-class weight was calculated exclusively from the training split using a log-scaled negative-to-positive imbalance ratio with clipping to the range 1–50. Specifically, the raw imbalance ratio was transformed as

$$w_{d}^{+}=clip[\ln\left(1+r_{d}\right),1,50]$$

where $w_{d}^{+}$ denotes the positive-class weight for disease, d indexes the 14 disease labels, and $r_{d}$ denotes the ratio of negative to positive training samples for disease d. The resulting $w_{d}^{+}$ was supplied to the pos_weight argument of BCEWithLogitsLoss. No focal loss, oversampling, undersampling, or other class-resampling strategy was used. Models were optimized using AdamW with an initial learning rate of 1 × 10^−4^ and weight decay of 1 × 10^−4^. A cosine learning-rate scheduler was used to gradually decrease the learning rate from the initial value of 1 × 10^−4^ to a minimum learning rate of 1 × 10^−6^. The maximum number of epochs was 100. Early stopping was based on validation loss with a patience of 10 epochs and min_delta = 0. The checkpoint with the lowest validation loss was retained for subsequent thresholding and ensemble analysis. Ten random seeds were used: 306, 346, 668, 696, 985, 797, 492, 775, 762, and 228. With five architectures and 10 seeds per architecture, a total of 50 training runs were completed. Detailed training settings are summarized in Supplementary Table S2.

### 2.3. Computational Cost and Training Runtime

Computational feasibility was assessed by recording training runtime across all model architectures and random seeds. Across all architectures and seeds, the total training time was 121.728 GPU-hours. Architecture-specific training runtime and stopping behavior are summarized in Supplementary Table S3. These results indicate that the major computational burden of the proposed framework arises from repeated training and inference across multiple base architectures and random seeds, whereas FAHP-based probability aggregation itself is computationally lightweight. In supplementary benchmarking on an NVIDIA GeForce RTX 3090 using a fixed 200-image test subset and TenCrop inference, mean model-only latency ranged from 22.97 ms/image for ViT to 61.36 ms/image for DenseNet121 + SwinV2, demonstrating architecture-dependent inference costs that should be considered in real-time deployment. The benchmarking protocol and computing environment are reported in Supplementary Table S4, and architecture-level latency, throughput, parameter count, and peak GPU memory are summarized in Supplementary Table S5.

### 2.4. Validation-Based Threshold Derivation

Each base model was treated as an independent multi-label classifier, producing one probability score per disease. For every architecture and random seed, disease-specific decision thresholds were determined on the validation set using Youden’s index. These thresholds were then fixed and applied unchanged to the corresponding test-set predictions to enable threshold-based performance evaluation. The validation set was used both for checkpoint selection and threshold derivation; however, no information from the held-out test set was used during model development or threshold derivation, thereby preventing direct test-set leakage. The derivation dataset, selection rule, locking status, and test-set role of the principal analysis components are summarized in Supplementary Table S6.

### 2.5. Soft Voting

Soft Voting was implemented as an equal-weight probability-level ensemble. For each disease and each random seed, the predicted probabilities from the five base models were first averaged to obtain a seed-level probability. These were subsequently aggregated across the 10 seeds using equal weights to produce final validation and test-set probabilities. Disease-specific decision thresholds were then determined on the aggregated validation probabilities using Youden’s index and were fixed and applied unchanged to the corresponding test-set predictions. This implementation therefore represents a two-stage equal-weight probability-level ensemble, consisting of averaging across model architectures followed by averaging across seed-level ensemble members.

### 2.6. Hard Voting

Hard Voting was implemented as a thresholded decision-level ensemble. For each disease, each model’s probability output was first converted into a binary prediction using its validation-derived, disease-specific threshold for the corresponding random seed. For threshold-dependent metrics, Hard Voting assigned a positive classification when at least three of the five base models produced positive predictions. For discrimination metrics, the model-level positive vote proportion, ranging from 0/5 to 5/5, was used as the score for area under the receiver operating characteristic curve (AUROC) and area under the precision–recall curve (AUPRC) calculation. Thus, seed-level AUROC and AUPRC for Hard Voting were computed from vote-proportion scores rather than from a single binary output. In the final-level analysis, seed-level Hard Voting outputs were aggregated using the positive-vote rate across the 10 seeds, and a positive-rate threshold of 0.5 was used for the final binary classification.

### 2.7. Fuzzy Analytic Hierarchy Process Ensemble

The proposed FAHP-based ensemble was implemented as a two-stage framework consisting of (1) disease-specific model weighting and (2) utility-guided seed aggregation. The fuzzy linguistic scale, pairwise-comparison configurations, defuzzified criterion weights, and consistency assessments for all predefined FAHP configurations are provided in Supplementary Table S7. These weights were fixed before test-set evaluation and were used to calculate disease-specific model-weighted FAHP scores. The primary FAHP configuration was defined as a sensitivity-priority scenario. After defuzzification, the criterion weights were 0.7646 for sensitivity, 0.1464 for specificity, and 0.0890 for precision. The approximate consistency measures were λmax ≈ 3.091, consistency index (CI) ≈ 0.046, and consistency ratio (CR) ≈ 0.079, indicating acceptable consistency of the pairwise comparison matrix under the conventional CR < 0.10 criterion. 

### 2.8. Disease-Specific Model Weighting

For each disease, the five base models were first evaluated on the validation set using their respective seed-specific, disease-specific Youden thresholds. Sensitivity, specificity, and precision were computed for each model and seed, and subsequently averaged across the 10 seeds to obtain a single validation performance triplet per model for that disease. Based on these aggregated performance metrics, a model-level validation matrix $M_{d}\in R^{5\times 3}$ was constructed for each disease d, where d = 1, …, 14 indexes the disease labels, the five rows correspond to the five base models, and the three columns correspond to sensitivity, specificity, and precision. The matrix $M_{d}$ was min–max-normalized separately for each criterion across the five models to produce the normalized performance matrix $Z_{d}\in {\left[0,1\right]}^{5\times 3}$. A linear weighted score was computed as:

$$S_{d,k}^{linear}=\sum_{c=1}^{3}Z_{d,k,c}\;w_{c},$$

where $S_{d,k}^{linear}$ denotes the linear weighted score for model k and disease d; k=1,…,5 indexes the five base models; d = 1, …, 14 indexes the 14 disease labels; c = 1, …, 3 indexes the three criteria of sensitivity, specificity, and precision; $Z_{d,k,c}$ denotes the normalized validation performance of model k for disease d under criterion c; and $w_{c}$ denotes the defuzzified FAHP weight to criterion c.

A parameter γ was used to increase separation among disease-specific model scores after FAHP-based weighting using the following equation:

$$S_{d,k}={\left(S_{d,k}^{linear}\right)}^{\gamma }.$$

where $S_{d,k}$ denotes the γ-transformed score for model k, disease d, and transformation factor γ. A value of γ = 2.0 was selected as the primary setting because it moderately accentuates higher-scoring models while avoiding overly sparse model weights. 

The resulting scores were normalized to obtain disease-specific model weights:

$$\alpha_{d,k}=\frac{S_{d,k}}{\sum_{j=1}^{5}S_{d,j}}.$$

where $\alpha_{d,k}$ denotes the normalized weight assigned to model k for disease d, and j = 1, …, 5 denotes the summation index over the five base models. This process yields a disease-specific weight vector across the five base models, reflecting the observation that no single architecture is uniformly optimal across sensitivity, specificity, and precision for all diseases. The resulting disease-specific FAHP architecture weights are reported in Supplementary Table S8.

### 2.9. Utility-Guided Seed Aggregation

Using the disease-specific model weights, a seed-level FAHP probability was computed for each disease:

$$p_{d,s}^{FAHP}(x)=\sum_{k=1}^{5}\alpha_{d,k}\;p_{d,s,k}(x),$$

where $p_{d,s,k}(x)$ is the predicted probability for image x from model k, seed s, and disease d. For each seed, a validation-based Youden threshold was determined on the corresponding FAHP probabilities. At this threshold, the net benefit (NB) was computed as:

$${NB}_{model}(p_{t})=\frac{TP}{N}-\frac{FP}{N}\times \frac{p_{t}}{1-p_{t}},$$

where TP and FP denote the numbers of true-positive and false-positive classifications, respectively, and N is the total number of validation samples, and $p_{t}$ denotes the probability threshold using the validation-derived Youden threshold for the corresponding disease and seed.

The baseline NB was defined as the maximum of the treat-none and treat-all strategies:

$${NB}_{baseline,s}\left(p_{t}\right)=\max\left({NB}_{treat-none},{NB}_{treat-all,s}\left(p_{t}\right)\right).$$

The ΔNet Benefit (ΔNB) was then calculated as:

$$\Delta {NB}_{s}={NB}_{model,s}(p_{t})-{NB}_{baseline,s}(p_{t}).$$

To obtain seed-level aggregation weights, a positive-utility weighting scheme was applied. The γ_NB_ parameter was predefined and was not optimized on the test set. A value of γ_NB_ = 1.0 was selected to preserve a linear relationship between positive ΔNB and seed-level aggregation weights. Sensitivity analyses for γ and γ_NB_ were subsequently performed to assess the robustness of the final ensemble to reasonable perturbations of γ and γ_NB_. For each seed *s*, the unnormalized seed weight ${\tilde{w}}_{s}$ was defined as:

$${\tilde{w}}_{s}=\varepsilon +(\Delta {NB}_{s}{)}^{\gamma_{NB}}\;\mathrm{if}\;\Delta {NB}_{s}>0,\;\mathrm{and}\;{\tilde{w}}_{s}=\varepsilon \;\mathrm{otherwise},\;\mathrm{where}\;\varepsilon =10^{-6}\;\mathrm{and}\;\gamma_{NB}=1.0.$$

The weights were normalized as follows:

$$w_{s}=\frac{{\tilde{w}}_{s}}{\sum_{r=1}^{10}{\tilde{w}}_{r}}$$

where $w_{s}$ denotes the normalized aggregation weight for seed s, and summation index r over the 10 seeds.

The final validation probability was obtained by weighted aggregation of seed-level FAHP probabilities:

$$p_{d}^{final}(x)=\sum_{s=1}^{10}w_{s}\;p_{d,s}^{FAHP}(x).$$

where $p_{d}^{final}(x)$ is the final predicted probability for image x from and disease d. 

A single disease-specific final threshold was then determined on the aggregated validation probabilities using Youden’s index and applied unchanged to the test set. Importantly, ΔNB was used solely as an auxiliary validation-derived weighting for seed-level probability aggregation. It was not used as a full decision curve analysis framework and was not used to optimize clinically actionable probability thresholds. Final decision thresholds were determined only after seed aggregation, using Youden’s validation-derived index. Therefore, the present ΔNB analysis should be interpreted as a utility-informed aggregation heuristic rather than as definitive evidence of clinical net benefit. The resulting disease-specific ΔNB-guided seed aggregation weights are provided in Supplementary Table S9.

### 2.10. Diagnostic Performance Evaluation

Diagnostic performance was evaluated on the held-out test set, with each disease treated as an independent binary classification task. The evaluated methods included the five single-model baselines, Hard Voting, Soft Voting, seed-level FAHP, final-level FAHP with equal seed aggregation, and the complete FAHP–ΔNB framework. CWO was evaluated only as a supplementary final-level comparator and was not a component of the proposed FAHP–ΔNB framework.

For the per-disease single-model comparator, the best base architecture was selected separately for each disease according to the highest mean validation-set AUROC across the 10 random seeds. FAHP architecture weights were derived from validation-set sensitivity, specificity, and precision combined with the predefined FAHP criteria weights. ΔNB seed weights were also derived from validation-set ΔNB values and fixed before test-set evaluation.

Discrimination performance was assessed using AUROC and AUPRC. Threshold-dependent performance was evaluated using sensitivity, specificity, precision, F1 score, negative predictive value (NPV), and balanced accuracy, where balanced accuracy was defined as the arithmetic mean of sensitivity and specificity. For seed-level analyses, performance metrics were summarized across the 10 random seeds using the mean and standard deviation. For final-level analyses, outputs from all 10 seeds were aggregated before evaluation, yielding a single disease-specific final estimate for each method.

Probability prediction error and calibration were evaluated as supplementary final-level analyses. The Brier score was calculated directly from final continuous predicted probabilities and was treated as a threshold-independent measure, with lower values indicating lower squared probability prediction error. Calibration intercept, calibration slope, and reliability curves were assessed for the principal final-level probability-based methods, including Soft Voting, CWO, FAHP, and FAHP–ΔNB. Hard Voting was excluded from calibration-intercept and calibration-slope analyses because its final output is a discrete positive vote proportion rather than a continuous model-derived probability.

### 2.11. Statistical Analysis

Statistical analyses were designed to characterize two distinct sources of uncertainty: seed-level variability associated with stochastic model training and patient-level sampling uncertainty in the held-out test cohort. Seed-level analyses were used to compare the five base architectures, Hard Voting, Soft Voting, and seed-level FAHP across the 10 paired random seeds. For each disease and each principal performance endpoint, overall differences among methods were assessed using the Friedman test, a non-parametric test suitable for comparing multiple related methods across paired seed-level measurements. Pairwise method comparisons were subsequently performed using two-sided Wilcoxon signed-rank tests on paired seed-level metric values, irrespective of the Friedman omnibus results. The Benjamini–Hochberg false discovery rate procedure was applied within each disease-by-metric family across the full set of tested method pairs. Final-level pairwise comparisons were conducted separately from the seed-level analyses. For these comparisons, the 14 disease labels were treated as paired units, and FAHP–ΔNB was compared with Hard Voting, Soft Voting, and final-level FAHP using two-sided Wilcoxon signed-rank tests. *p* values were adjusted using the Benjamini–Hochberg procedure within each performance metric. These final-level comparisons were interpreted as exploratory disease-wise comparisons rather than seed-level inferential tests.

To quantify patient-level sampling uncertainty in final-level performance, disease-stratified patient-cluster bootstrap resampling was performed on the held-out test set. The patient, rather than the individual CXR, was used as the resampling unit, and all CXRs belonging to a sampled patient were retained together. For each disease label, 2000 bootstrap replicates were generated while preserving the disease-specific patient-level positive-to-negative composition of the test cohort. Model training, architecture selection, FAHP architecture weights, ΔNB seed weights, CWO weights, and validation-derived operating thresholds were not re-estimated during bootstrap resampling. AUROC, AUPRC, balanced accuracy, F1 score, and Brier score were recalculated in each bootstrap replicate. Method-specific 95% confidence intervals and paired bootstrap confidence intervals were obtained from the 2.5th and 97.5th percentiles of the corresponding bootstrap distributions. The patient-cluster bootstrap confidence intervals were not multiplicity-adjusted simultaneous confidence intervals and were therefore interpreted as descriptive uncertainty estimates rather than multiplicity-controlled confirmatory hypothesis tests. All statistical tests were two-sided. Statistical significance was denoted as * for p<0.05, ** for p<0.01, and *** for p<0.001 after adjustment where applicable.

## 3. Results

### 3.1. Overall Performance Across Diseases

Overall performance across the 14 diseases is summarized in Table 1. Among the five single-model architectures, DenseNet121 achieved the highest AUROC (0.842 ± 0.003), whereas DenseNet121 + SwinV2 showed the highest AUPRC (0.277 ± 0.005). Overall, DenseNet121, SwinV2 Transformer, and the hybrid models showed closely comparable discrimination performance. Vision Transformer showed comparatively lower performance across most metrics.

Compared with the single-model baselines, probability-level ensembles demonstrated improved discrimination performance, whereas Hard Voting showed inferior performance. Soft Voting achieved the highest overall performance, with mean ± SD values of 0.851 ± 0.001 for AUROC and 0.293 ± 0.003 for AUPRC. The proposed FAHP ensemble showed closely comparable performance, with corresponding values of 0.850 ± 0.001 and 0.287 ± 0.004.

For threshold-based metrics, Soft Voting and FAHP showed similar balanced accuracy (0.775 ± 0.002 vs. 0.774 ± 0.002), whereas FAHP exhibited a slightly lower F1 score (0.233 ± 0.005 vs. 0.236 ± 0.004), reflecting its sensitivity-priority configuration. In contrast, Hard Voting showed substantially lower discrimination performance, particularly for AUPRC (0.169 ± 0.002), despite comparable balanced accuracy.

These results suggest that probability-level aggregation is more effective than thresholded decision-level voting for preserving discrimination performance in multi-label CXR classification. Soft Voting provided a strong equal-weight reference, while seed-level FAHP offered comparable overall performance with the added advantage of clinician-configurable, disease-specific model weighting. Utility-guided seed aggregation was evaluated separately in the final-level FAHP–ΔNB analysis. Seed-level analyses under alternative FAHP preference configurations showed similar overall performance across sensitivity-priority, specificity-priority, and balanced settings, indicating that the disease-specific FAHP model-weighting stage was robust to the predefined preference configuration before final ΔNB-guided seed aggregation (Supplementary Table S10).

### 3.2. Per-Disease Performance Comparison

Per-disease performance comparisons are presented in Table 2. The relative performance of FAHP and Soft Voting varied across disease labels. FAHP demonstrated favorable discrimination or balanced-accuracy profiles, including Effusion, Emphysema, Mass, Nodule, and Pleural Thickening. For example, FAHP achieved higher AUROC and AUPRC in Emphysema compared with Soft Voting, whereas F1 score was lower, reflecting a shift in operating characteristics. In Effusion, FAHP showed higher AUROC, AUPRC, and balanced accuracy, whereas F1 score was comparable to Soft Voting. In Mass and Nodule, FAHP improved discrimination metrics, including AUROC and AUPRC, but did not consistently improve F1 score, indicating a shift in operating characteristics rather than uniform gains across all endpoints. Conversely, Soft Voting remained favorable in several diseases, including Atelectasis, Cardiomegaly, Fibrosis, Hernia, and Infiltration, particularly for discrimination metrics. Overall, these findings indicate that FAHP does not uniformly outperform equal-weight aggregation but instead enables disease-specific and preference-dependent reconfiguration of ensemble behavior.

### 3.3. Pairwise Statistical Comparisons

Seed-level statistical comparisons are summarized in Table 3. Mean differences were calculated as FAHP minus the comparator method. Friedman omnibus tests were used to assess overall differences among methods across paired random seeds, and the complete disease-specific omnibus results are provided in Supplementary Table S11. Pairwise method comparisons were subsequently performed using two-sided Wilcoxon signed-rank tests, with Benjamini–Hochberg-adjusted *p* values derived from the complete disease-by-metric comparison families.

Compared with the best single-model comparator and Hard Voting, FAHP showed consistent improvements in AUROC and AUPRC across most diseases. In contrast, comparisons between FAHP and Soft Voting revealed a mixed pattern. FAHP achieved statistically significant improvements over Soft Voting in selected diseases, such as Effusion, Emphysema, Mass, Nodule, and Pleural Thickening for selected discrimination or threshold-based endpoints. However, statistically significant decreases were also observed in other diseases, including Atelectasis, Cardiomegaly, Fibrosis, Hernia, and Infiltration for selected metrics.

For F1 score, FAHP exhibited a preference-dependent pattern. Under the current sensitivity-priority configuration, small positive mean differences relative to Soft Voting were observed in Cardiomegaly, Consolidation, Fibrosis, Hernia, and Pneumonia, whereas negative mean differences were observed in Atelectasis, Edema, Effusion, Emphysema, Infiltration, Mass, Nodule, Pleural Thickening, and Pneumothorax. These results support the interpretation that FAHP primarily shifts disease-specific operating characteristics rather than producing uniform improvement across all endpoints.

### 3.4. Final-Level Comparison After 10-Seed Aggregation

Final-level performance after aggregation across all 10 random seeds is summarized in Table 4. In this analysis, each disease–method pair yielded a single final estimate, allowing for a direct comparison among Hard Voting, Soft Voting, FAHP, and FAHP–ΔNB. Overall, FAHP–ΔNB showed final-level performance broadly comparable to FAHP and Soft Voting, while consistently outperforming Hard Voting in discrimination metrics, particularly AUROC and AUPRC. Disease-stratified patient-cluster bootstrap confidence intervals for final-level performance, including AUROC, AUPRC, balanced accuracy, F1 score, and Brier score, are reported in Supplementary Table S12.

Focused final-level pairwise comparisons across the 14 disease labels are presented in Table 5. FAHP–ΔNB outperformed Hard Voting for AUROC and AUPRC across all 14 diseases, with significant adjusted *p* values. Compared with Soft Voting and FAHP, however, differences were small and not statistically significant for most metrics. FAHP–ΔNB showed a small, statistically nonsignificant increase in balanced accuracy compared with FAHP, and no consistent improvement was observed for F1 score, sensitivity, specificity, or precision. Patient-level sampling uncertainty for these final-level method comparisons was further evaluated using paired disease-stratified patient-cluster bootstrap analysis, with 95% empirical percentile confidence intervals reported in Supplementary Table S13. These findings indicate that ΔNB-guided seed aggregation provides a utility-informed mechanism for modifying final operating characteristics but should not be interpreted as producing uniform performance gains across all metrics or disease labels.

At the final level, the macro-averaged Brier scores were 0.0446 for Soft Voting, 0.0437 for CWO, 0.0447 for FAHP, and 0.0444 for FAHP–ΔNB. CWO showed the lowest macro-averaged Brier score, whereas FAHP–ΔNB showed a slightly lower probability prediction error than Soft Voting and FAHP. The absolute differences among probability-level methods were small (Supplementary Table S14). In the supplementary ablation analysis, CWO achieved the lowest macro-averaged Brier score (0.0437), but its AUROC (0.8518), AUPRC (0.2959), and balanced accuracy (0.7751) were lower than those of FAHP–ΔNB (0.8549, 0.2986, and 0.7804, respectively), illustrating the different trade-offs produced by direct probability-error optimization and preference- and utility-guided weighting (Supplementary Table S15). Disease-specific calibration curves for the principal final-level probability-based ensemble methods are shown in Supplementary Figure S1. These reliability curves compare predicted probabilities with observed event proportions using 10 quantile-based probability bins and provide a visual assessment of calibration across Soft Voting, CWO, FAHP, and FAHP–ΔNB.

Sensitivity analyses showed that final FAHP–ΔNB performance was stable across the tested γ and γ_NB_ values. Across γ = 1.0–3.0, macro-averaged AUROC varied only from 0.8540 to 0.8553 and balanced accuracy from 0.7789 to 0.7804; across γ_NB_ = 0.5–2.0, the corresponding ranges were 0.8548–0.8550 and 0.7792–0.7804, respectively (Supplementary Table S16).

Calibration intercepts were generally negative and calibration slopes were greater than one across disease–method combinations, indicating a tendency toward probability overestimation and insufficient dispersion of predicted log-odds. These patterns varied by disease and method and were interpreted descriptively rather than as evidence of post hoc recalibration (Supplementary Figure S1).

Supplementary preference analyses demonstrated that overall performance remained similar across FAHP configurations, while operating characteristics varied according to the specified criterion priorities. At the final FAHP–ΔNB level, the sensitivity-priority configuration achieved the highest sensitivity (0.7840) and balanced accuracy (0.7804); the specificity-priority configuration achieved the highest specificity (0.7973), precision (0.1542), and F1 score (0.2383), as well as the lowest Brier score (0.0441); and the balanced configuration achieved the highest AUROC (0.8559) and AUPRC (0.3020) (Supplementary Table S17). Disease-specific analyses further demonstrated heterogeneous preference effects. For example, in Cardiomegaly, sensitivity decreased from 0.881 under the sensitivity-priority configuration to 0.845 under the specificity-priority configuration, whereas specificity increased from 0.823 to 0.844. For Nodule, specificity increased from 0.741 to 0.839 as the configuration shifted from the sensitivity-priority configuration to the specificity-priority configuration (Supplementary Table S18). Importantly, prioritizing a criterion did not deterministically maximize that metric for every disease, because preference-dependent model weighting was followed by ΔNB-guided seed aggregation and configuration-specific validation-derived thresholding (Supplementary Table S18).

### 3.5. Seed-Level Variability Analysis

Seed-level variability in sensitivity across methods is illustrated in Figure 3. Soft Voting exhibited relatively stable performance across seeds, reflecting the smoothing effect of equal-weight probability averaging. Hard Voting showed limited variability but generally lower sensitivity. In contrast, FAHP demonstrated disease-dependent variability patterns, with increased sensitivity in some diseases but greater dispersion in others. Because Figure 3 represents the disease-specific FAHP model-weighting stage before final seed aggregation, these findings indicate that model-level weighting alone can alter seed-level sensitivity profiles across diseases.

Similar patterns were observed for precision variability, as shown in Figure 4. Compared with Soft Voting, FAHP exhibited disease-dependent changes in precision, with reductions in some conditions, including Emphysema, Effusion, Infiltration, and Mass. These findings indicate that FAHP may shift operating characteristics toward other criteria, such as sensitivity and balanced accuracy, rather than uniformly improving precision.

Representative Grad-CAM-based spatial comparisons are shown in Figure 5. Representative examples were selected from a pre-generated final-level Grad-CAM candidate manifest derived from the held-out test set. The visualization manifest was generated separately from the quantitative performance analyses and did not alter any reported test-set result. Ground-truth-positive cases were prioritized to permit visual inspection of spatial attention for CXRs containing the target disease label.

Figure 5 presents four representative ground-truth-positive examples under the primary sensitivity-priority FAHP configuration, including Pneumothorax, Cardiomegaly, Effusion, and Edema. These examples illustrate how visualization-level spatial summaries may differ when alternative architecture- and seed-aggregation strategies are applied. The displayed Grad-CAM maps should be interpreted as qualitative post hoc spatial summaries rather than quantitative localization results, because bounding-box or pixel-level lesion annotations were not available for formal localization assessment.

Across the illustrated cases, the ensemble methods showed heterogeneous spatial emphasis within the thoracic region, with differences in the extent and concentration of activation across aggregation strategies. FAHP and FAHP–ΔNB frequently produced broadly similar spatial patterns because both methods share the same disease-specific FAHP architecture weights, whereas differences between them arise from the additional ΔNB-guided seed weighting used by FAHP–ΔNB. CWO could exhibit more localized or differently distributed activation because its architecture weights were obtained by direct validation-set Brier score optimization rather than by the FAHP multi-criteria preference structure. These qualitative differences should not be interpreted as evidence of anatomically correct localization or superiority of one ensemble method in lesion localization.

## 4. Discussion

This study developed and evaluated a clinician-configurable ensemble framework based on the FAHP for multi-label CXR classification. The proposed two-stage framework—consisting of disease-specific model weighting and utility-guided seed aggregation—was designed to address key limitations of conventional ensemble approaches that rely on equal-weight aggregation. Overall, the results support our hypothesis that a preference-aware and utility-informed ensemble can achieve performance comparable to equal-weight methods while improving interpretability and configurability.

Although both the present study and our prior work utilize the NIH ChestX-ray14 dataset [7], the two studies differ fundamentally in research objectives and methodological design. Our previous work focused on architecture-level innovation and external validation of hybrid CNN–transformer models, whereas the present study addresses ensemble-level decision modeling through a clinician-configurable FAHP framework grounded in multi-criteria decision analysis for healthcare decision-making [14]. Importantly, all models, training processes, and evaluation outputs in this study were independently generated, with no reuse of previously published model weights or predictions, ensuring experimental independence.

A principal finding of this study is that Soft Voting remains a strong and stable baseline across multiple diseases and performance metrics. Equal-weight probability-level aggregation can leverage complementary information across model architectures and is consistent with recent evidence that ensemble learning and simple statistical pooling can improve robustness and performance in DL-based medical image classification [15,16]. In our experiments, Soft Voting achieved the highest overall discrimination performance, highlighting the difficulty of improving upon a strong equal-weight baseline.

Within this context, the proposed FAHP-based framework demonstrated comparable overall performance while offering distinct advantages in flexibility and interpretability. Unlike conventional ensemble methods, FAHP enables explicit incorporation of clinician-defined priorities through multi-criteria weighting of sensitivity, specificity, and precision [17,18]. This approach is grounded in multi-criteria decision analysis, which has been widely applied in healthcare decision-making to formalize trade-offs among competing objectives and incorporate stakeholder preferences [14,19,20]. By integrating such criteria into model aggregation, the proposed method provides a structured and transparent mechanism for aligning AI predictions with clinical priorities. The interpretability provided by the proposed FAHP framework should be understood as aggregation-level transparency rather than intrinsic spatial interpretability of the underlying deep networks. Specifically, FAHP makes explicit how sensitivity, specificity, and precision contribute to disease-specific model weighting and how validation-derived utility signals influence seed-level aggregation. As a qualitative explainability assessment, Grad-CAM-based ensemble visualizations of representative true-positive test cases are shown in Figure 5. These maps illustrate the spatial evidence emphasized by the component models and their weighted combinations under different aggregation strategies, but they should not be interpreted as causal explanations or formal lesion-localization validation.

Another key contribution of this study is the introduction of disease-specific model weighting. Our results confirm that no single architecture consistently dominates across all diseases and performance criteria, consistent with recent studies emphasizing pathology-aware model design, long-tailed label distributions, and disease-specific challenges in multi-label CXR classification [21,22,23]. By assigning weights separately for each disease, the FAHP framework adapts to disease-dependent performance characteristics, resulting in favorable disease-specific performance profiles in selected conditions such as Effusion, Emphysema, Mass, Nodule, and Pleural Thickening. These findings suggest that ensemble design may benefit from moving beyond global weighting strategies toward disease-aware configurations.

In addition to inter-model variability, this study addressed seed-level stochastic variability through utility-guided aggregation. Net benefit is a clinically interpretable metric derived from decision curve analysis that relates prediction threshold probabilities to the relative consequences of false-positive and false-negative decisions [6,24,25]. In the present framework, however, ΔNB was not used as a full decision curve analysis framework and was not used to optimize clinically actionable probability thresholds. Instead, validation-derived ΔNB was used only as an auxiliary weighting signal for aggregating seed-level FAHP probabilities. This design emphasizes ensemble members that showed positive validation-set utility while preserving a separate validation-based thresholding procedure using Youden’s index. Accordingly, the present ΔNB analysis should be interpreted as a utility-informed aggregation heuristic rather than as definitive evidence of clinical net benefit. Future work should evaluate net benefit over prespecified clinically relevant threshold-probability ranges and incorporate formal probability recalibration and assess its effect on clinical decision utility before clinical deployment.

The Brier score analysis provided complementary evidence regarding probability prediction error. In the supplementary calibration analysis, CWO achieved the lowest macro-averaged Brier score (0.0437), whereas FAHP–ΔNB showed a slightly lower Brier score (0.0444) than Soft Voting (0.0446) and FAHP (0.0447). The absolute differences among probability-level ensemble methods were small, and none of the evaluated methods achieved ideal calibration across all diseases.

Despite these advantages, the performance of FAHP relative to Soft Voting was heterogeneous across diseases and metrics. In some diseases, FAHP achieved improvements in AUROC, AUPRC, or balanced accuracy, whereas in others, Soft Voting remained superior. These results highlight a fundamental distinction between probability-level aggregation and thresholded decision-level voting. Probability-level methods preserve continuous model-output information, whereas thresholded decision-level methods such as Hard Voting binarize predictions before aggregation and may therefore discard confidence information, resulting in reduced discrimination performance [16,26]. Furthermore, under the current sensitivity-priority configuration, trade-offs were observed in F1 score, reflecting shifts in the operating point of the ensemble rather than uniform improvements across all metrics.

The variability analyses presented in Figure 3 and Figure 4 provide additional insight into how disease-specific FAHP model weighting modifies ensemble behavior beyond average performance metrics. Because these analyses were performed at the seed level before final ΔNB-guided seed aggregation, they specifically reflect the effect of model-level FAHP weighting on sensitivity and precision variability. FAHP exhibited disease-dependent changes in sensitivity (Figure 3), with increased sensitivity in some conditions but greater variability across seeds compared with Soft Voting. Complementary patterns were observed in precision (Figure 4), where FAHP showed disease-dependent changes, including reductions in Emphysema, Effusion, Infiltration, and Mass compared with Soft Voting. These findings indicate that FAHP model weighting can shift operating characteristics toward other criteria, such as sensitivity or balanced accuracy, rather than uniformly improving precision. Together, these findings indicate that FAHP model weighting induces a systematic shift in the operating point of the ensemble, producing disease-specific trade-offs between sensitivity and precision. This observation is consistent with recent reports that multi-label CXR classification is affected by disease-specific heterogeneity, label imbalance, and pathology-dependent performance variability [21]. The use of criterion-specific weighting further aligns with contemporary healthcare multi-criteria decision analysis frameworks, which emphasize explicit trade-offs among competing performance criteria and stakeholder preferences [14]. These patterns provide a mechanistic explanation for the heterogeneous performance observed across diseases. In contrast, Soft Voting demonstrated more stable behavior across seeds for both metrics, consistent with the smoothing effect of equal-weight probability aggregation.

Collectively, these observations underscore a key distinction between the proposed approach and conventional ensemble methods. Rather than aiming to maximize a single performance metric, the FAHP framework enables controlled, preference-aware reconfiguration of model behavior. This design allows the ensemble to adapt to different clinical priorities, such as emphasizing sensitivity in screening or balancing specificity in confirmatory settings, while maintaining transparency in how model contributions are weighted.

Several limitations should be acknowledged. First, the study was conducted using a single public dataset (NIH ChestX-ray14), which may limit generalizability to other populations and imaging settings. External validation on independent datasets is necessary to confirm robustness. Second, the primary clinician preference model was based on predefined pairwise comparisons emphasizing sensitivity rather than formal preference elicitation from a clinician panel. Supplementary analyses of sensitivity-priority, specificity-priority, and balanced configurations demonstrated that alternative preferences produced modest but measurable shifts in discrimination and disease-specific operating characteristics. Nevertheless, these predefined scenarios do not substitute for empirically elicited clinician preferences, which should be evaluated prospectively. Third, the same validation set was used for checkpoint selection, threshold derivation, estimation of FAHP performance criteria, ΔNB-based seed weighting, and final ensemble threshold selection. Although no test-set information was used and therefore no direct test-set leakage occurred, this design may increase dependence on a single validation set and may lead to validation overfitting, particularly for rare labels such as Hernia and Pneumonia. Future studies should consider nested validation, separate calibration and ensemble-construction subsets, or resampling-based strategies to reduce validation-set dependence. Fourth, while ΔNB provides a clinically meaningful utility signal, it was applied only as an auxiliary validation-derived weighting mechanism rather than within a full decision-analytic optimization framework. Because the ΔNB calculation in this study used validation-derived Youden thresholds rather than prespecified clinically meaningful threshold probabilities, the resulting ΔNB values should be interpreted as utility-informed aggregation signals rather than definitive evidence of clinical net benefit. Future work should evaluate net benefit over clinically prespecified threshold-probability ranges and should investigate formal probability recalibration before clinical deployment. Fifth, γ = 2.0 and γ_NB_ = 1.0 were predefined design parameters rather than formally optimized hyperparameters. Supplementary sensitivity analyses showed that the principal findings were stable across the tested γ and γ_NB_ values; however, broader hyperparameter evaluation and prospective validation remain warranted before clinical deployment. Sixth, this study focused primarily on aggregation-level interpretability. Although Grad-CAM-based visualizations were added to provide qualitative spatial examples, these analyses were not radiologist-validated and did not include bounding-box or pixel-level lesion annotations. Future work should incorporate expert-annotated lesion regions and quantitative localization metrics to determine whether ensemble decisions are anatomically consistent with clinically relevant findings.

## 5. Conclusions

In conclusion, we present a clinician-configurable and disease-aware ensemble framework for multi-label CXR classification. The proposed FAHP–ΔNB framework maintained performance comparable to strong equal-weight probability-level baselines while enabling explicit, disease-specific incorporation of configurable diagnostic priorities. Supplementary preference analyses further demonstrated that sensitivity-priority, specificity-priority, and balanced configurations produced similar overall discrimination but different operating characteristics, supporting the configurability of the framework at the aggregation level. These features may support more transparent and context-aware ensemble construction in radiology AI research. However, external validation and prospective clinical evaluation are required before clinical deployment.

## Figures and Tables

**Figure 1:**
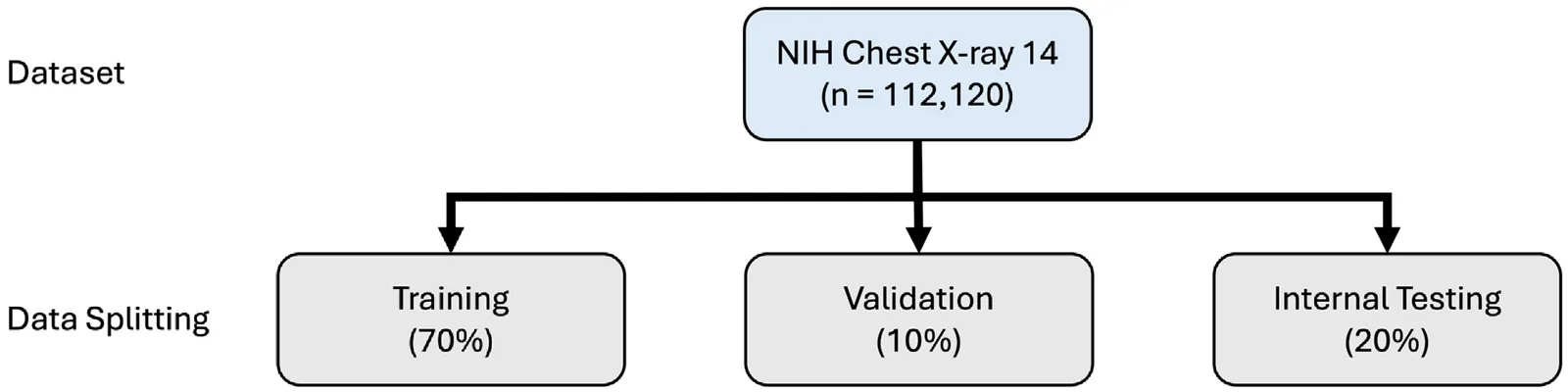
Overview of dataset preparation and data partitioning. A publicly available frontal NIH ChestX-ray14 dataset was used in this study. The training set was used for model development. The validation set was used for checkpoint/model selection, threshold derivation, and ensemble configuration, whereas the internal test set was reserved for final performance evaluation. NIH denotes National Institutes of Health.

**Figure 2:**
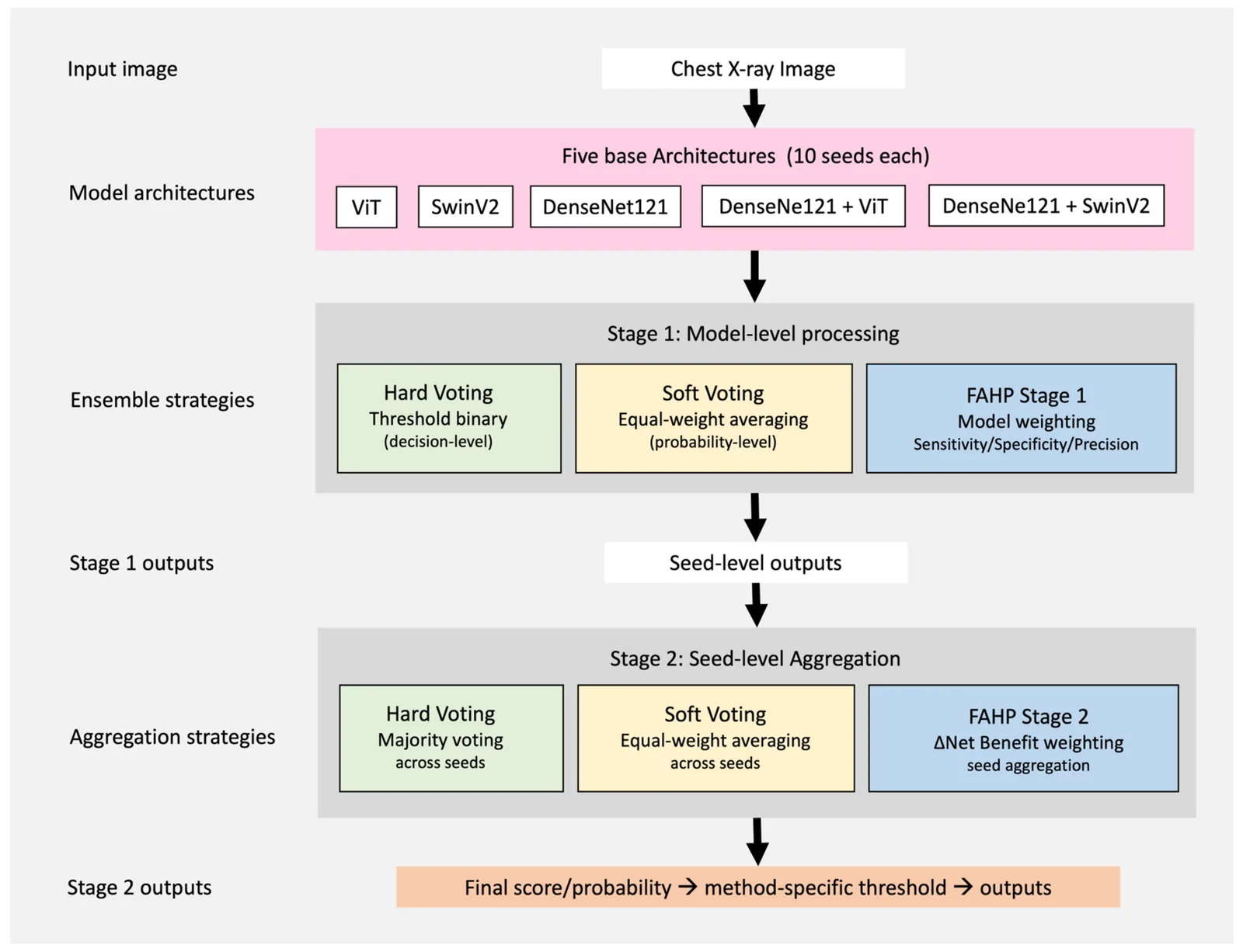
Overview of the proposed FAHP-based two-stage ensemble framework and comparison with conventional ensemble strategies for multi-label chest X-ray classification. ViT denotes Vision Transformer; FAHP denotes fuzzy analytic hierarchy process.

**Figure 3:**
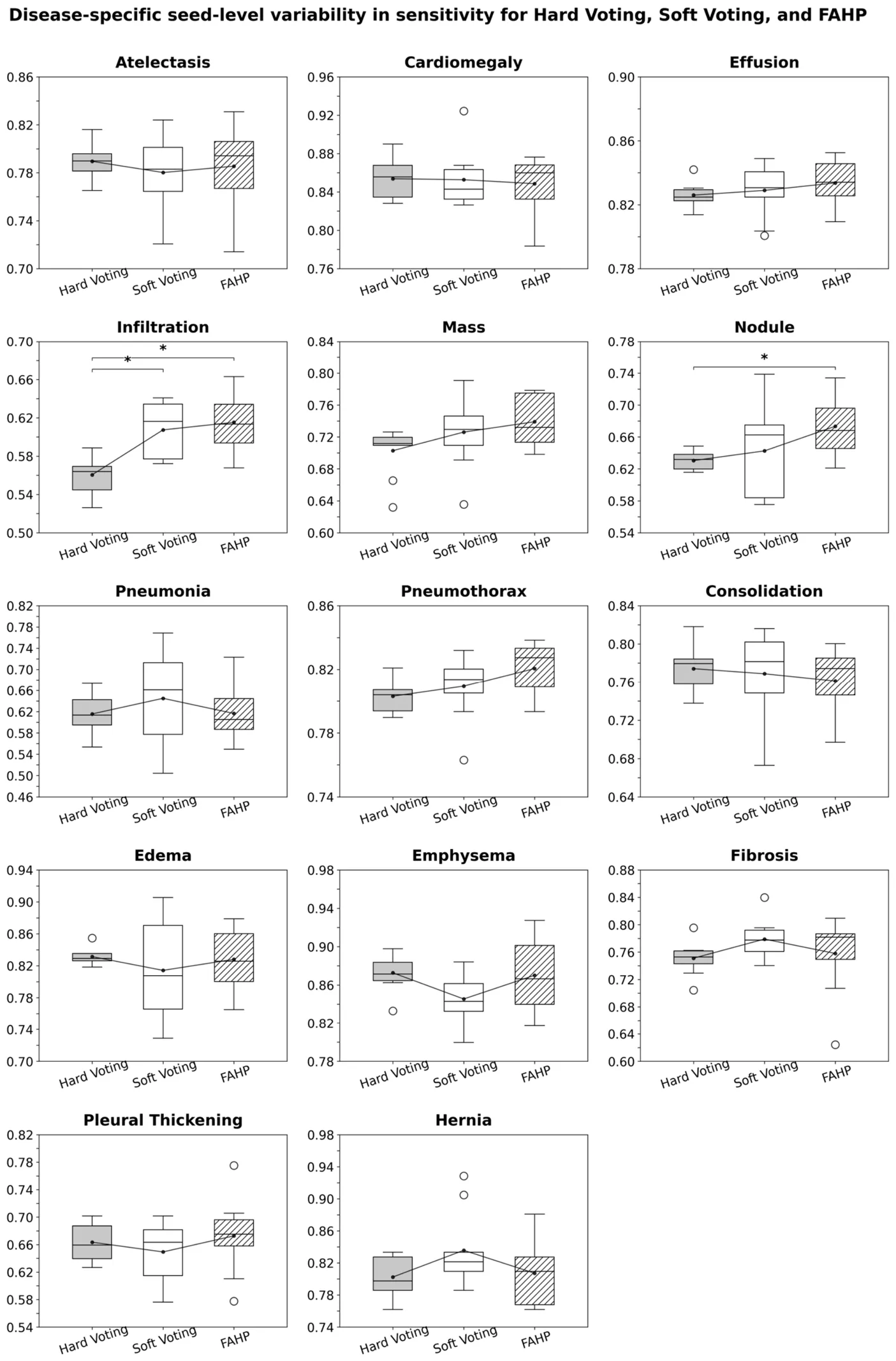
Disease-specific seed-level variability in sensitivity for Hard Voting, Soft Voting, and FAHP. Boxplots show the distribution of test-set sensitivity across 10 random seeds for Hard Voting, Soft Voting, and FAHP. Statistical significance is indicated as *p* < 0.05 (*) after Benjamini–Hochberg correction. Circles represent outliers—data points that fall significantly above the upper quartile or below the lower quartile. FAHP denotes fuzzy analytic hierarchy process.

**Figure 4:**
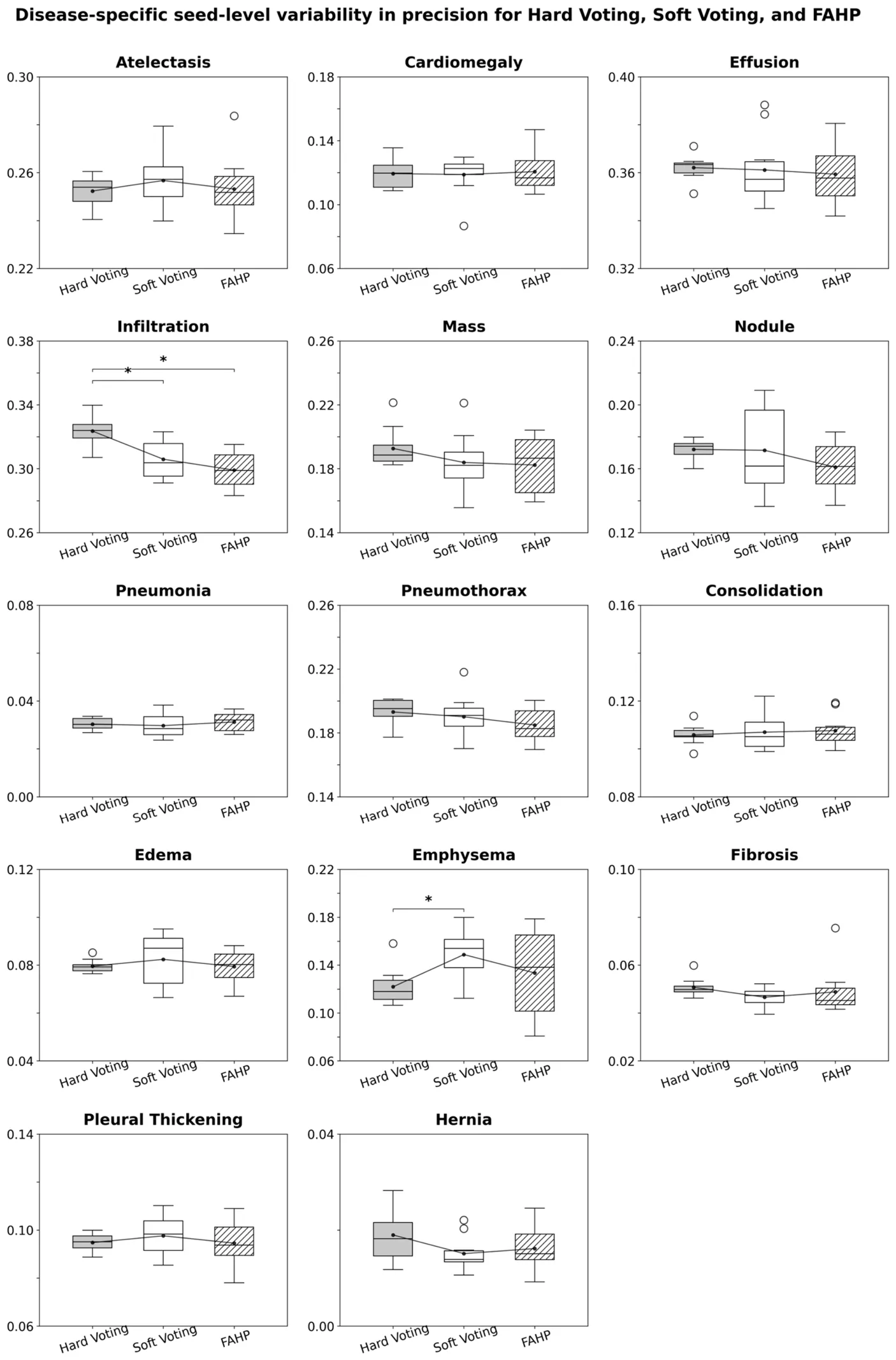
Disease-specific seed-level variability in precision for Hard Voting, Soft Voting, and FAHP. Boxplots illustrate the distribution of test-set precision values across 10 random seeds for each disease. FAHP represents disease-specific model-weighted probability fusion before final 10-seed aggregation. Statistical significance is indicated as *p* < 0.05 (*) after Benjamini–Hochberg correction. Circles represent outliers—data points that fall significantly above the upper quartile or below the lower quartile. FAHP denotes fuzzy analytic hierarchy process.

**Figure 5:**
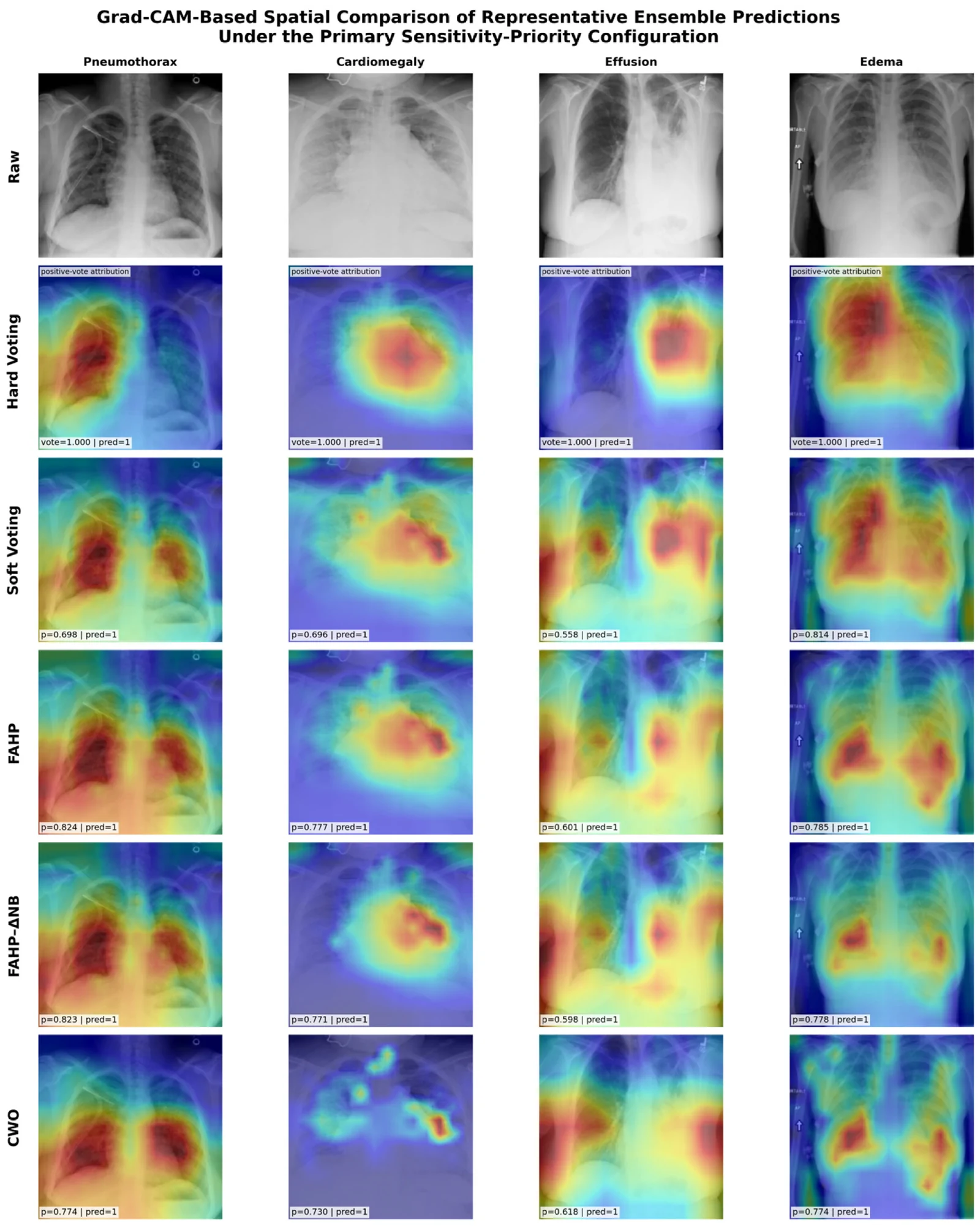
Grad-CAM-based spatial comparison of representative ensemble predictions under the primary sensitivity-priority configuration. Four representative ground-truth-positive examples from the held-out test set are shown for Pneumothorax, Cardiomegaly, Effusion, and Edema. Columns correspond to the target disease examples, and rows show the original chest X-ray (Raw) followed by visualization-level Grad-CAM summaries for Hard Voting, Soft Voting, FAHP, FAHP–ΔNB, and CWO. For Soft Voting, FAHP, FAHP–ΔNB, and CWO, p denotes the corresponding final aggregated disease probability. For Hard Voting, p denotes the final positive-vote rate across the 10 seed-level Hard Voting decisions. “pred” denotes the final binary prediction according to the corresponding method-specific decision rule. Heatmaps indicate the relative spatial distribution of target-class Grad-CAM activation within each image. Because the activation maps were normalized for visualization, absolute heatmap intensity should not be compared quantitatively across methods or cases. These visualizations are post hoc spatial summaries and should not be interpreted as radiologist-validated lesion localization. FAHP represents fuzzy analytic hierarchy process, FAHP–ΔNB represents FAHP with ΔNet Benefit, and CWO represents constrained weight optimization. The phrase of positive vote attribution denotes Grad-CAM maps from base models with positive binary predictions, determined using validation-derived disease- and seed-specific thresholds, that contributed to a positive seed-level Hard Voting decision.

**Table 1:** Macro-averaged diagnostic performance across 14 diseases for single-model architectures and ensemble methods.

Method	AUROC	AUPRC	Sensitivity	Specificity	Precision	F1 Score	Balanced Accuracy
ViT	0.808 ± 0.003	0.210 ± 0.005	0.723 ± 0.009	0.744 ± 0.009	0.125 ± 0.003	0.197 ± 0.003	0.733 ± 0.004
SwinV2	0.840 ± 0.002	0.270 ± 0.006	0.735 ± 0.007	0.791 ± 0.008	0.146 ± 0.003	0.227 ± 0.004	0.763 ± 0.003
DenseNet121	0.842 ± 0.003	0.276 ± 0.003	0.739 ± 0.016	0.790 ± 0.013	0.146 ± 0.005	0.228 ± 0.005	0.764 ± 0.003
DenseNet121 + ViT	0.836 ± 0.003	0.275 ± 0.005	0.740 ± 0.012	0.783 ± 0.010	0.144 ± 0.003	0.224 ± 0.005	0.762 ± 0.002
DenseNet121 + SwinV2	0.838 ± 0.002	0.277 ± 0.005	0.738 ± 0.016	0.789 ± 0.015	0.145 ± 0.005	0.226 ± 0.007	0.763 ± 0.002
Hard Voting	0.822 ± 0.001	0.169 ± 0.002	0.748 ± 0.007	0.800 ± 0.006	0.151 ± 0.002	0.235 ± 0.003	0.774 ± 0.001
Soft Voting	0.851 ± 0.001	0.293 ± 0.003	0.756 ± 0.013	0.793 ± 0.011	0.151 ± 0.004	0.236 ± 0.004	0.775 ± 0.002
FAHP	0.850 ± 0.001	0.287 ± 0.004	0.759 ± 0.008	0.789 ± 0.009	0.148 ± 0.004	0.233 ± 0.005	0.774 ± 0.002

Note: Values are presented as mean ± standard deviation across 10 random seeds. For each seed, performance was first macro-averaged across the 14 disease labels and then summarized across seeds. AUROC denotes area under the receiver operating characteristic curve; AUPRC denotes area under the precision–recall curve; ViT denotes vision transformer; FAHP denotes fuzzy analytic hierarchy process.

**Table 2:** Per-disease diagnostic performance comparison of the best single model and ensemble methods on the NIH ChestX-ray14 test set.

Disease	Method	AUROC	AUPRC	Sensitivity	Specificity	Precision	F1 Score	Balanced Accuracy
Atelectasis	DenseNet121 + SwinV2	0.824 ± 0.003	0.367 ± 0.005	0.792 ± 0.028	0.704 ± 0.030	0.245 ± 0.013	0.374 ± 0.012	0.748 ± 0.003
	Hard Voting	0.798 ± 0.003	0.268 ± 0.005	0.790 ± 0.013	0.717 ± 0.014	0.252 ± 0.006	0.382 ± 0.006	0.753 ± 0.002
	Soft Voting	0.830 ± 0.001	0.379 ± 0.003	0.780 ± 0.029	0.726 ± 0.025	0.257 ± 0.011	0.386 ± 0.008	0.753 ± 0.003
	FAHP	0.829 ± 0.002	0.376 ± 0.004	0.785 ± 0.032	0.718 ± 0.030	0.253 ± 0.013	0.382 ± 0.011	0.752 ± 0.002
Cardiomegaly	SwinV2	0.908 ± 0.004	0.303 ± 0.012	0.843 ± 0.025	0.815 ± 0.027	0.109 ± 0.011	0.193 ± 0.016	0.829 ± 0.008
	Hard Voting	0.892 ± 0.002	0.156 ± 0.008	0.854 ± 0.020	0.831 ± 0.017	0.119 ± 0.009	0.209 ± 0.013	0.842 ± 0.003
	Soft Voting	0.920 ± 0.002	0.322 ± 0.004	0.853 ± 0.028	0.828 ± 0.031	0.119 ± 0.012	0.208 ± 0.018	0.841 ± 0.004
	FAHP	0.919 ± 0.002	0.320 ± 0.006	0.849 ± 0.027	0.833 ± 0.023	0.121 ± 0.012	0.211 ± 0.018	0.841 ± 0.004
Consolidation	SwinV2	0.812 ± 0.003	0.165 ± 0.004	0.767 ± 0.065	0.704 ± 0.053	0.105 ± 0.011	0.185 ± 0.014	0.736 ± 0.008
	Hard Voting	0.782 ± 0.003	0.111 ± 0.002	0.774 ± 0.022	0.708 ± 0.020	0.106 ± 0.004	0.186 ± 0.005	0.741 ± 0.003
	Soft Voting	0.817 ± 0.001	0.173 ± 0.003	0.769 ± 0.044	0.711 ± 0.037	0.107 ± 0.007	0.187 ± 0.010	0.740 ± 0.005
	FAHP	0.816 ± 0.002	0.171 ± 0.003	0.761 ± 0.035	0.717 ± 0.030	0.108 ± 0.006	0.188 ± 0.008	0.739 ± 0.003
Edema	DenseNet121 + SwinV2	0.890 ± 0.004	0.156 ± 0.007	0.802 ± 0.035	0.816 ± 0.023	0.076 ± 0.006	0.139 ± 0.010	0.809 ± 0.008
	Hard Voting	0.875 ± 0.004	0.094 ± 0.002	0.831 ± 0.009	0.819 ± 0.007	0.080 ± 0.002	0.145 ± 0.004	0.825 ± 0.004
	Soft Voting	0.904 ± 0.002	0.173 ± 0.003	0.814 ± 0.059	0.825 ± 0.038	0.082 ± 0.011	0.149 ± 0.017	0.820 ± 0.011
	FAHP	0.904 ± 0.002	0.170 ± 0.004	0.828 ± 0.035	0.818 ± 0.024	0.079 ± 0.007	0.145 ± 0.011	0.823 ± 0.008
Effusion	DenseNet121	0.880 ± 0.003	0.524 ± 0.008	0.816 ± 0.013	0.794 ± 0.014	0.357 ± 0.012	0.496 ± 0.009	0.805 ± 0.002
	Hard Voting	0.852 ± 0.002	0.388 ± 0.004	0.826 ± 0.007	0.796 ± 0.006	0.362 ± 0.005	0.503 ± 0.004	0.811 ± 0.001
	Soft Voting	0.887 ± 0.001	0.540 ± 0.003	0.829 ± 0.016	0.794 ± 0.016	0.361 ± 0.014	0.503 ± 0.011	0.812 ± 0.001
	FAHP	0.888 ± 0.001	0.541 ± 0.003	0.834 ± 0.014	0.792 ± 0.013	0.359 ± 0.011	0.502 ± 0.008	0.813 ± 0.001
Emphysema	SwinV2	0.927 ± 0.007	0.401 ± 0.019	0.848 ± 0.035	0.852 ± 0.022	0.119 ± 0.011	0.208 ± 0.017	0.850 ± 0.010
	Hard Voting	0.913 ± 0.002	0.181 ± 0.016	0.873 ± 0.018	0.852 ± 0.020	0.122 ± 0.014	0.213 ± 0.021	0.862 ± 0.005
	Soft Voting	0.939 ± 0.002	0.409 ± 0.003	0.845 ± 0.023	0.884 ± 0.024	0.149 ± 0.021	0.252 ± 0.030	0.865 ± 0.005
	FAHP	0.940 ± 0.002	0.414 ± 0.004	0.870 ± 0.037	0.854 ± 0.058	0.133 ± 0.036	0.229 ± 0.053	0.862 ± 0.012
Fibrosis	SwinV2	0.831 ± 0.005	0.131 ± 0.009	0.734 ± 0.032	0.763 ± 0.031	0.049 ± 0.005	0.092 ± 0.008	0.748 ± 0.004
	Hard Voting	0.816 ± 0.003	0.064 ± 0.004	0.751 ± 0.023	0.768 ± 0.022	0.051 ± 0.004	0.095 ± 0.006	0.760 ± 0.003
	Soft Voting	0.844 ± 0.003	0.150 ± 0.005	0.779 ± 0.026	0.736 ± 0.031	0.047 ± 0.004	0.088 ± 0.006	0.758 ± 0.004
	FAHP	0.841 ± 0.002	0.148 ± 0.004	0.758 ± 0.052	0.748 ± 0.051	0.049 ± 0.010	0.091 ± 0.016	0.753 ± 0.004
Hernia	DenseNet121	0.948 ± 0.014	0.410 ± 0.042	0.771 ± 0.071	0.940 ± 0.039	0.032 ± 0.016	0.061 ± 0.029	0.856 ± 0.027
	Hard Voting	0.919 ± 0.009	0.058 ± 0.017	0.802 ± 0.024	0.916 ± 0.025	0.019 ± 0.005	0.037 ± 0.010	0.859 ± 0.011
	Soft Voting	0.943 ± 0.007	0.447 ± 0.039	0.836 ± 0.043	0.892 ± 0.026	0.015 ± 0.003	0.030 ± 0.006	0.864 ± 0.012
	FAHP	0.934 ± 0.011	0.355 ± 0.055	0.807 ± 0.039	0.898 ± 0.036	0.016 ± 0.005	0.032 ± 0.009	0.853 ± 0.012
Infiltration	SwinV2	0.706 ± 0.003	0.345 ± 0.005	0.556 ± 0.034	0.742 ± 0.031	0.316 ± 0.012	0.402 ± 0.003	0.649 ± 0.003
	Hard Voting	0.689 ± 0.003	0.301 ± 0.004	0.561 ± 0.019	0.750 ± 0.019	0.324 ± 0.009	0.410 ± 0.003	0.655 ± 0.002
	Soft Voting	0.717 ± 0.002	0.353 ± 0.002	0.607 ± 0.028	0.705 ± 0.029	0.306 ± 0.011	0.406 ± 0.004	0.656 ± 0.002
	FAHP	0.714 ± 0.002	0.349 ± 0.003	0.615 ± 0.028	0.692 ± 0.029	0.299 ± 0.011	0.402 ± 0.005	0.654 ± 0.003
Mass	DenseNet121 + SwinV2	0.851 ± 0.003	0.306 ± 0.006	0.719 ± 0.046	0.820 ± 0.038	0.179 ± 0.022	0.285 ± 0.024	0.769 ± 0.007
	Hard Voting	0.828 ± 0.005	0.208 ± 0.008	0.703 ± 0.029	0.842 ± 0.016	0.193 ± 0.012	0.302 ± 0.011	0.773 ± 0.007
	Soft Voting	0.859 ± 0.002	0.319 ± 0.003	0.726 ± 0.041	0.826 ± 0.028	0.184 ± 0.017	0.292 ± 0.018	0.776 ± 0.007
	FAHP	0.862 ± 0.002	0.321 ± 0.004	0.739 ± 0.032	0.821 ± 0.029	0.182 ± 0.017	0.292 ± 0.020	0.780 ± 0.003
Nodule	DenseNet121 + SwinV2	0.784 ± 0.006	0.263 ± 0.006	0.633 ± 0.040	0.794 ± 0.032	0.165 ± 0.014	0.261 ± 0.014	0.714 ± 0.005
	Hard Voting	0.768 ± 0.004	0.176 ± 0.003	0.630 ± 0.011	0.808 ± 0.011	0.172 ± 0.006	0.270 ± 0.007	0.719 ± 0.004
	Soft Voting	0.795 ± 0.002	0.276 ± 0.002	0.643 ± 0.054	0.796 ± 0.051	0.172 ± 0.025	0.268 ± 0.026	0.719 ± 0.003
	FAHP	0.798 ± 0.002	0.282 ± 0.002	0.673 ± 0.037	0.774 ± 0.038	0.161 ± 0.015	0.259 ± 0.017	0.724 ± 0.003
Pleural Thickening	DenseNet121	0.789 ± 0.010	0.144 ± 0.007	0.689 ± 0.060	0.742 ± 0.043	0.084 ± 0.007	0.149 ± 0.010	0.715 ± 0.013
	Hard Voting	0.770 ± 0.004	0.096 ± 0.003	0.663 ± 0.026	0.785 ± 0.017	0.095 ± 0.003	0.166 ± 0.004	0.724 ± 0.005
	Soft Voting	0.796 ± 0.003	0.151 ± 0.004	0.649 ± 0.044	0.794 ± 0.031	0.098 ± 0.008	0.169 ± 0.010	0.722 ± 0.008
	FAHP	0.798 ± 0.004	0.155 ± 0.004	0.673 ± 0.051	0.778 ± 0.039	0.095 ± 0.009	0.165 ± 0.012	0.726 ± 0.008
Pneumonia	SwinV2	0.762 ± 0.006	0.048 ± 0.005	0.585 ± 0.054	0.799 ± 0.050	0.032 ± 0.005	0.060 ± 0.008	0.692 ± 0.008
	Hard Voting	0.747 ± 0.008	0.031 ± 0.002	0.616 ± 0.039	0.783 ± 0.029	0.030 ± 0.002	0.058 ± 0.004	0.699 ± 0.009
	Soft Voting	0.775 ± 0.005	0.052 ± 0.003	0.645 ± 0.083	0.760 ± 0.063	0.030 ± 0.005	0.057 ± 0.008	0.703 ± 0.013
	FAHP	0.773 ± 0.006	0.051 ± 0.003	0.617 ± 0.051	0.786 ± 0.043	0.031 ± 0.004	0.059 ± 0.007	0.701 ± 0.010
Pneumothorax	DenseNet121 + SwinV2	0.879 ± 0.004	0.345 ± 0.012	0.802 ± 0.018	0.805 ± 0.021	0.174 ± 0.012	0.286 ± 0.015	0.804 ± 0.005
	Hard Voting	0.863 ± 0.003	0.234 ± 0.008	0.803 ± 0.010	0.828 ± 0.011	0.193 ± 0.008	0.311 ± 0.010	0.816 ± 0.003
	Soft Voting	0.890 ± 0.002	0.364 ± 0.002	0.809 ± 0.019	0.823 ± 0.018	0.190 ± 0.013	0.308 ± 0.016	0.816 ± 0.004
	FAHP	0.890 ± 0.002	0.366 ± 0.005	0.821 ± 0.016	0.815 ± 0.015	0.185 ± 0.010	0.302 ± 0.012	0.818 ± 0.003

Note: Values are presented as mean ± standard deviation across 10 random seeds. For each disease, the best-performing single model was selected based on validation performance and compared with Hard Voting, Soft Voting, and the FAHP-based ensemble. AUROC denotes area under the receiver operating characteristic curve; AUPRC denotes area under the precision–recall curve; FAHP denotes fuzzy analytic hierarchy process; NIH denotes National Institutes of Health.

**Table 3:** Pairwise seed-level comparisons between FAHP and comparator methods for each disease and primary endpoint.

Disease	Metric	Comparator	Mean Difference	*p* Value
Atelectasis	AUROC	DenseNet121 + SwinV2	0.005	0.002 **
		Hard Voting	0.031	0.003 **
		Soft Voting	−0.001	0.003 **
	AUPRC	DenseNet121 + SwinV2	0.009	0.003 **
		Hard Voting	0.108	0.003 **
		Soft Voting	−0.003	0.005 **
	Balanced Accuracy	DenseNet121 + SwinV2	0.004	0.006 **
		Hard Voting	−0.001	0.065
		Soft Voting	−0.001	0.376
	F1	DenseNet121 + SwinV2	0.008	0.104
		Hard Voting	−0.000	0.749
		Soft Voting	−0.004	0.325
Cardiomegaly	AUROC	SwinV2	0.011	0.003 **
		Hard Voting	0.026	0.003 **
		Soft Voting	−0.001	0.003 **
	AUPRC	SwinV2	0.016	0.003 **
		Hard Voting	0.164	0.003 **
		Soft Voting	−0.003	0.047 *
	Balanced Accuracy	SwinV2	0.012	0.004 **
		Hard Voting	−0.002	0.862
		Soft Voting	0.000	1
	F1	SwinV2	0.018	0.027 *
		Hard Voting	0.002	0.862
		Soft Voting	0.003	0.811
Consolidation	AUROC	SwinV2	0.005	0.005 **
		Hard Voting	0.035	0.003 **
		Soft Voting	−0.001	0.042 *
	AUPRC	SwinV2	0.006	0.006 **
		Hard Voting	0.060	0.003 **
		Soft Voting	−0.002	0.032 *
	Balanced Accuracy	SwinV2	0.003	0.335
		Hard Voting	−0.002	0.236
		Soft Voting	−0.001	0.335
	F1	SwinV2	0.004	0.602
		Hard Voting	0.002	0.602
		Soft Voting	0.001	0.829
Edema	AUROC	DenseNet121 + SwinV2	0.014	0.003 **
		Hard Voting	0.029	0.003 **
		Soft Voting	−0.001	0.118
	AUPRC	DenseNet121 + SwinV2	0.014	0.003 **
		Hard Voting	0.075	0.003 **
		Soft Voting	−0.003	0.032 *
	Balanced Accuracy	DenseNet121 + SwinV2	0.014	0.046 *
		Hard Voting	−0.003	0.709
		Soft Voting	0.003	0.779
	F1	DenseNet121 + SwinV2	0.006	0.501
		Hard Voting	−0.0005	1
		Soft Voting	−0.004	0.602
Effusion	AUROC	DenseNet121	0.007	0.003 **
		Hard Voting	0.036	0.003 **
		Soft Voting	0.000	0.012 *
	AUPRC	DenseNet121	0.017	0.003 **
		Hard Voting	0.153	0.003 **
		Soft Voting	0.001	0.410
	Balanced Accuracy	DenseNet121	0.008	0.003 **
		Hard Voting	0.002	0.050 *
		Soft Voting	0.001	0.038 *
	F1	DenseNet121	0.006	0.525
		Hard Voting	−0.001	0.877
		Soft Voting	−0.001	0.922
Emphysema	AUROC	SwinV2	0.013	0.003 **
		Hard Voting	0.027	0.003 **
		Soft Voting	0.001	0.035 *
	AUPRC	SwinV2	0.013	0.128
		Hard Voting	0.233	0.003 **
		Soft Voting	0.005	0.008 **
	Balanced Accuracy	SwinV2	0.012	0.016 *
		Hard Voting	−0.000	0.922
		Soft Voting	−0.003	0.922
	F1	SwinV2	0.021	0.457
		Hard Voting	0.015	0.451
		Soft Voting	−0.023	0.574
Fibrosis	AUROC	SwinV2	0.010	0.003 **
		Hard Voting	0.026	0.003 **
		Soft Voting	−0.003	0.003 **
	AUPRC	SwinV2	0.017	0.003 **
		Hard Voting	0.084	0.003 **
		Soft Voting	−0.002	0.035 *
	Balanced Accuracy	SwinV2	0.005	0.086
		Hard Voting	−0.007	0.023 *
		Soft Voting	−0.005	0.107
	F1	SwinV2	−0.000	0.593
		Hard Voting	−0.004	0.261
		Soft Voting	0.003	0.987
Hernia	AUROC	DenseNet121	−0.014	0.217
		Hard Voting	0.015	0.014 *
		Soft Voting	−0.009	0.004 **
	AUPRC	DenseNet121	−0.056	0.018 *
		Hard Voting	0.297	0.003 **
		Soft Voting	−0.092	0.003 **
	Balanced Accuracy	DenseNet121	−0.003	0.457
		Hard Voting	−0.0063	0.410
		Soft Voting	−0.0112	0.138
	F1	DenseNet121	−0.0295	0.064
		Hard Voting	−0.0054	0.525
		Soft Voting	0.002	0.829
Infiltration	AUROC	SwinV2	0.008	0.003 **
		Hard Voting	0.025	0.003 **
		Soft Voting	−0.003	0.003 **
	AUPRC	SwinV2	0.005	0.086
		Hard Voting	0.048	0.003 **
		Soft Voting	−0.004	0.003 **
	Balanced Accuracy	SwinV2	0.005	0.017 *
		Hard Voting	−0.002	0.187
		Soft Voting	−0.003	0.017 *
	F1	SwinV2	0.000	0.922
		Hard Voting	−0.008	0.006 **
		Soft Voting	−0.004	0.046 *
Mass	AUROC	DenseNet121 + SwinV2	0.011	0.003 **
		Hard Voting	0.034	0.003 **
		Soft Voting	0.003	0.003 **
	AUPRC	DenseNet121 + SwinV2	0.014	0.003 **
		Hard Voting	0.113	0.003 **
		Soft Voting	0.002	0.059
	Balanced Accuracy	DenseNet121 + SwinV2	0.011	0.004 **
		Hard Voting	0.007	0.014 *
		Soft Voting	0.004	0.047 *
	F1	DenseNet121 + SwinV2	0.006	0.979
		Hard Voting	−0.010	0.551
		Soft Voting	−0.001	1
Nodule	AUROC	DenseNet121 + SwinV2	0.014	0.003 **
		Hard Voting	0.031	0.003 **
		Soft Voting	0.004	0.003 **
	AUPRC	DenseNet121 + SwinV2	0.019	0.003 **
		Hard Voting	0.106	0.003 **
		Soft Voting	0.006	0.003 **
	Balanced Accuracy	DenseNet121 + SwinV2	0.010	0.004 **
		Hard Voting	0.005	0.018 *
		Soft Voting	0.005	0.006 **
	F1	DenseNet121 + SwinV2	−0.002	0.877
		Hard Voting	−0.011	0.164
		Soft Voting	−0.010	0.729
Pleural Thickening	AUROC	DenseNet121	0.010	0.003 **
		Hard Voting	0.029	0.003 **
		Soft Voting	0.002	0.018 *
	AUPRC	DenseNet121	0.011	0.003 **
		Hard Voting	0.059	0.003 **
		Soft Voting	0.003	0.003 **
	Balanced Accuracy	DenseNet121	0.010	0.024 *
		Hard Voting	0.001	0.577
		Soft Voting	0.004	0.224
	F1	DenseNet121	0.016	0.042 *
		Hard Voting	−0.001	1
		Soft Voting	−0.004	0.877
Pneumonia	AUROC	SwinV2	0.011	0.006 **
		Hard Voting	0.026	0.003 **
		Soft Voting	−0.002	0.167
	AUPRC	SwinV2	0.003	0.118
		Hard Voting	0.020	0.004 **
		Soft Voting	−0.001	0.037 *
	Balanced Accuracy	SwinV2	0.009	0.042 *
		Hard Voting	0.002	0.956
		Soft Voting	−0.001	1
	F1	SwinV2	−0.001	0.848
		Hard Voting	0.002	0.779
		Soft Voting	0.003	0.779
Pneumothorax	AUROC	DenseNet121 + SwinV2	0.011	0.003 **
		Hard Voting	0.027	0.003 **
		Soft Voting	−0.000	0.577
	AUPRC	DenseNet121 + SwinV2	0.021	0.003 **
		Hard Voting	0.132	0.003 **
		Soft Voting	0.002	0.623
	Balanced Accuracy	DenseNet121 + SwinV2	0.014	0.003 **
		Hard Voting	0.002	0.179
		Soft Voting	0.002	0.153
	F1	DenseNet121 + SwinV2	0.016	0.087
		Hard Voting	−0.010	0.181
		Soft Voting	−0.006	0.451

Note: Pairwise comparisons were performed using seed-level test-set results across 10 random seeds. Mean differences were calculated as FAHP minus the comparator method. For each disease, the single-model comparator corresponds to the best-performing base architecture. *p* values were obtained using two-sided Wilcoxon signed-rank tests and adjusted using the Benjamini–Hochberg procedure within each disease–metric family. Statistical significance is indicated as *p* < 0.05 (*) and *p* < 0.01 (**) after adjustment. These pairwise comparisons are intended to characterize disease- and metric-specific differences between FAHP and comparator methods and should not be interpreted as evidence of uniform improvement across all endpoints. AUROC denotes area under the receiver operating characteristic curve; AUPRC denotes area under the precision–recall curve; FAHP denotes fuzzy analytic hierarchy process.

**Table 4:** Per-disease final-level performance comparison of Hard Voting, Soft Voting, FAHP, and FAHP–ΔNB after aggregation across 10 random seeds.

Disease	Method	AUROC	AUPRC	Sensitivity	Specificity	Precision	F1 Score	Balanced Accuracy
Atelectasis	Hard Voting	0.791	0.259	0.801	0.713	0.253	0.384	0.757
	Soft Voting	0.833	0.383	0.809	0.705	0.249	0.381	0.757
	FAHP	0.833	0.382	0.777	0.733	0.260	0.310	0.755
	FAHP–ΔNB	0.833	0.382	0.781	0.732	0.260	0.390	0.756
Cardiomegaly	Hard Voting	0.892	0.152	0.869	0.829	0.119	0.209	0.849
	Soft Voting	0.923	0.328	0.871	0.825	0.117	0.207	0.848
	FAHP	0.922	0.328	0.861	0.836	0.123	0.215	0.848
	FAHP–ΔNB	0.923	0.327	0.881	0.823	0.117	0.207	0.852
Consolidation	Hard Voting	0.773	0.107	0.784	0.703	0.105	0.186	0.743
	Soft Voting	0.819	0.177	0.803	0.685	0.102	0.181	0.744
	FAHP	0.819	0.176	0.812	0.678	0.101	0.180	0.745
	FAHP–ΔNB	0.819	0.176	0.812	0.678	0.101	0.180	0.745
Edema	Hard Voting	0.866	0.087	0.843	0.816	0.079	0.145	0.810
	Soft Voting	0.906	0.176	0.872	0.794	0.074	0.136	0.833
	FAHP	0.907	0.174	0.872	0.798	0.075	0.138	0.835
	FAHP–ΔNB	0.906	0.175	0.881	0.787	0.072	0.133	0.834
Effusion	Hard Voting	0.841	0.370	0.831	0.794	0.361	0.503	0.813
	Soft Voting	0.889	0.544	0.849	0.777	0.348	0.493	0.813
	FAHP	0.889	0.545	0.832	0.797	0.365	0.507	0.815
	FAHP–ΔNB	0.889	0.545	0.866	0.759	0.335	0.483	0.812
Emphysema	Hard Voting	0.909	0.173	0.882	0.850	0.120	0.212	0.866
	Soft Voting	0.944	0.414	0.859	0.884	0.146	0.250	0.871
	FAHP	0.945	0.421	0.874	0.871	0.136	0.235	0.873
	FAHP–ΔNB	0.945	0.421	0.847	0.901	0.166	0.278	0.874
Fibrosis	Hard Voting	0.812	0.061	0.760	0.763	0.050	0.094	0.761
	Soft Voting	0.848	0.155	0.823	0.693	0.042	0.080	0.758
	FAHP	0.846	0.154	0.823	0.685	0.041	0.078	0.754
	FAHP–ΔNB	0.846	0.154	0.823	0.685	0.041	0.078	0.754
Hernia	Hard Voting	0.897	0.048	0.810	0.922	0.019	0.037	0.866
	Soft Voting	0.950	0.482	0.833	0.862	0.011	0.022	0.848
	FAHP	0.937	0.430	0.810	0.893	0.014	0.028	0.851
	FAHP–ΔNB	0.942	0.452	0.833	0.897	0.015	0.030	0.865
Infiltration	Hard Voting	0.682	0.293	0.565	0.750	0.325	0.412	0.657
	Soft Voting	0.720	0.355	0.588	0.728	0.315	0.410	0.658
	FAHP	0.720	0.354	0.636	0.679	0.297	0.405	0.657
	FAHP–ΔNB	0.720	0.354	0.627	0.689	0.300	0.406	0.658
Mass	Hard Voting	0.822	0.206	0.721	0.842	0.195	0.307	0.781
	Soft Voting	0.863	0.324	0.711	0.848	0.199	0.311	0.780
	FAHP	0.868	0.327	0.767	0.815	0.181	0.293	0.791
	FAHP–ΔNB	0.868	0.327	0.770	0.813	0.180	0.292	0.792
Nodule	Hard Voting	0.763	0.180	0.639	0.808	0.174	0.273	0.723
	Soft Voting	0.799	0.281	0.628	0.820	0.181	0.281	0.724
	FAHP	0.804	0.288	0.705	0.741	0.147	0.243	0.723
	FAHP–ΔNB	0.804	0.288	0.705	0.741	0.147	0.243	0.723
Pleural Thickening	Hard Voting	0.769	0.097	0.676	0.783	0.095	0.167	0.729
	Soft Voting	0.800	0.154	0.631	0.813	0.103	0.176	0.722
	FAHP	0.803	0.160	0.650	0.803	0.100	0.174	0.726
	FAHP–ΔNB	0.801	0.154	0.683	0.771	0.092	0.162	0.727
Pneumonia	Hard Voting	0.741	0.030	0.641	0.779	0.031	0.059	0.710
	Soft Voting	0.778	0.053	0.727	0.682	0.024	0.047	0.705
	FAHP	0.779	0.054	0.657	0.759	0.029	0.055	0.708
	FAHP–ΔNB	0.779	0.054	0.657	0.759	0.029	0.055	0.708
Pneumothorax	Hard Voting	0.858	0.224	0.812	0.828	0.194	0.313	0.820
	Soft Voting	0.894	0.370	0.780	0.839	0.202	0.322	0.819
	FAHP	0.894	0.373	0.808	0.839	0.204	0.326	0.824
	FAHP–ΔNB	0.894	0.373	0.812	0.838	0.203	0.325	0.825

Note. Values are final-level estimates obtained after aggregating outputs from all 10 random seeds; therefore, each disease–method pair has a single final estimate, and no seed-level standard deviation is reported. FAHP–ΔNB represents FAHP model-weighted fusion followed by validation-derived ΔNB-guided seed aggregation. For Consolidation, Fibrosis, Nodule, and Pneumonia, none of the 10 validation seeds had a positive ΔNB value; therefore, FAHP–ΔNB reverted to equal seed weighting and yielded identical final-level results to FAHP for these diseases. AUROC denotes area under the receiver operating characteristic curve; AUPRC denotes area under the precision–recall curve; FAHP denotes fuzzy analytic hierarchy process; FAHP–ΔNB represents FAHP with ΔNet Benefit.

**Table 5:** Focused final-level pairwise comparisons between FAHP–ΔNB and comparator methods across 14 disease labels.

Metric	Comparator	Mean Difference	Win/Loss/Tie	Adjusted *p* Value
AUROC	Hard Voting	0.039	14/0/0	0.000 ***
	Soft Voting	0.000	9/5/0	0.355
	FAHP	0.000	6/4/4	0.879
AUPRC	Hard Voting	0.135	14/0/0	0.000 ***
	Soft Voting	−0.001	7/7/0	0.903
	FAHP	0.001	4/6/4	0.690
Balanced Accuracy	Hard Voting	0.001	7/7/0	0.469
	Soft Voting	0.003	9/5/0	0.185
	FAHP	0.002	8/2/4	0.185
F1 score	Hard Voting	−0.003	3/11/0	0.695
	Soft Voting	−0.003	6/8/0	0.700
	FAHP	−0.000	4/6/4	0.695
Sensitivity	Hard Voting	0.025	11/2/1	0.115
	Soft Voting	0.012	9/3/2	0.260
	FAHP	0.007	8/2/4	0.228
Specificity	Hard Voting	−0.022	3/11/0	0.147
	Soft Voting	−0.006	4/10/0	0.429
	FAHP	−0.004	3/7/4	0.427
Precision	Hard Voting	−0.004	3/11/0	0.650
	Soft Voting	−0.004	6/8/0	0.650
	FAHP	−0.001	4/6/4	0.650

Note: Values represent final-level pairwise comparisons using the 14 disease labels as paired units. Mean differences were calculated as FAHP–ΔNB minus the comparator method. Win/Loss/Tie indicates the number of disease labels for which FAHP–ΔNB showed higher, lower, or identical performance than the comparator. Ties in the FAHP–ΔNB versus FAHP comparison occurred because, for Consolidation, Fibrosis, Nodule, and Pneumonia, none of the 10 validation seeds had a positive ΔNB value; therefore, FAHP–ΔNB reverted to equal seed weighting and yielded identical final-level results to FAHP for these diseases. Results should be interpreted as exploratory final-level comparisons across disease labels rather than seed-level inferential tests. Statistical significance is indicated as *p* < 0.001 (***) after Benjamini–Hochberg correction. AUROC denotes area under the receiver operating characteristic curve; AUPRC denotes area under the precision–recall curve; FAHP denotes fuzzy analytic hierarchy process; FAHP–ΔNB represents FAHP with ΔNet Benefit.

## Data Availability

The original contributions presented in this study are included in the article/Supplementary Materials. Further inquiries can be directed to the corresponding authors.

## Supplementary Materials

The following supporting information can be downloaded at: https://www.mdpi.com/article/10.3390/diagnostics16193132/s1. Figure S1: Disease-specific calibration curves of the principal final-level ensemble methods under the primary sensitivity-priority configuration; Table S1: Dataset split and leakage audit; Table S2: Training configuration and reproducibility; Table S3: Training computational cost; Table S4: Inference benchmark configuration and computing environment; Table S5: Computational efficiency of the five architectures; Table S6: Validation-derived analysis provenance and aggregation strategies; Table S7: FAHP fuzzy linguistic scale, pairwise-comparison configurations, and consistency assessment; Table S8: Disease-specific FAHP architecture weights under the primary sensitivity-priority configuration; Table S9: Disease-specific ΔNet Benefit-guided seed aggregation weights under the primary sensitivity-priority configuration; Table S10: Seed-level performance of FAHP weighted fusion under alternative preference configurations; Table S11: Friedman omnibus test results for seed-level method comparisons under the primary sensitivity-priority FAHP configuration; Table S12: Disease-stratified patient-cluster bootstrap confidence intervals for final-level performance under the primary sensitivity-priority FAHP configuration; Table S13: Paired disease-stratified patient-cluster bootstrap comparisons of FAHP–ΔNB with final-level comparator methods; Table S14: Disease-specific probability calibration metrics of the principal final-level ensemble methods on the held-out test set; Table S15: Component-wise ablation and conventional weighting comparison under the primary sensitivity-priority configuration; Table S16: Sensitivity analysis of the FAHP model-weighting exponent γ and the ΔNet Benefit seed-weighting exponent $\gamma_{NB}$; Table S17: Final-level macro-averaged performance of FAHP–ΔNB under alternative FAHP preference configurations; Table S18: Disease-specific final-level operating characteristics of FAHP–ΔNB under alternative preference configurations.

## Abbreviations

The following abbreviations are used in this manuscript:

AI	Artificial Intelligence
AUPRC	Area Under the Precision–Recall Curve
AUROC	Area Under the Receiver Operating Characteristic Curve
CI	Consistency Index
CNN	Convolutional Neural Network
CR	Consistency Ratio
CWO	Constrained Weight Optimization
CXR	Chest X-ray
DL	Deep Learning
FAHP	Fuzzy Analytic Hierarchy Process
NIH	National Institutes of Health
NPV	Negative Predictive Value
ViT	Vision Transformer
ΔNB	ΔNet Benefit
