# Supplementary material for: A Clinician-Configurable FAHP-Based Ensemble Framework for Multi-Label Chest X-Ray Classification
Source: Diagnostics (Basel). 2026 Sep 26;16(19):3132. doi: 10.3390/diagnostics16193132 (PMC13664622; doi:10.3390/diagnostics16193132)
Supplement: Supplementary file 1 [file diagnostics-16-03132-s001.zip › diagnostics-4326142-supplementary.pdf]

Supplementary Material

Supplementary S1. Dataset Partition and Leakage Audit

The NIH ChestX-ray14 dataset was partitioned at the patient level into training, validation, and held-out test subsets before model development. Patient identifiers were used as the grouping unit, and all radiographs belonging to the same patient were assigned to a single partition. The resulting patient and image assignments were retained as fixed split lists and were reused unchanged across all base architectures, random seeds, ensemble-construction procedures, and subsequent analyses. No repartitioning was performed across model architectures or training runs.

The final fixed partition consisted of 21,528 training patients with 78,468 images, 3,090 validation patients with 11,219 images, and 6,187 held-out test patients with 22,433 images. To verify the absence of data leakage, explicit image-level and patient-level overlap checks were performed for each pair of partitions. Dataset counts and leakage-audit results are summarized below.

Supplementary Table S1. Dataset split and leakage audit

Part A. Dataset partition

Split	Images	Patients
Training	78,468	21,528
Validation	11,219	3,090
Test	22,433	6,187
**Total**	112,120	30,805

Part B. Leakage audit

Comparison	Image overlap	Patient overlap
Train vs Validation	0	0
Train vs Test	0	0
Validation vs Test	0	0

Note: All dataset partitions were defined at the patient level before model development and were subsequently retained as fixed split assignments throughout all experiments. All radiographs belonging to the same patient were restricted to a single subset. The same training, validation, and held-out test partitions were used for all five architectures and all 10 random seeds. No repartitioning was performed during model training, ensemble construction, threshold derivation, or final evaluation. Explicit overlap checks confirmed that neither images nor patients were shared across the training, validation, and held-out test subsets.

Supplementary S2. Model Training and Computational Efficiency

This appendix provides detailed information on model training, computational cost, and inference benchmarking. These materials complement the methodological descriptions provided in the main manuscript and document the procedures used to ensure training reproducibility and computational transparency.

Training Configuration and Reproducibility

Five base architectures were independently trained using 10 predefined random seeds, resulting in 50 planned training runs. The same preprocessing, class-imbalance handling, optimization strategy, early-stopping rule, and checkpoint-selection procedure were applied consistently across experiments. Detailed training settings are summarized below.

Supplementary Table S2. Training configuration and reproducibility

Item	Setting
Architectures	5
Random seeds	306, 346, 668, 696, 985, 797, 492, 775, 762, 228
Total training runs	50
Input size	224 × 224
Training augmentation	RandomResizedCrop (scale 0.9–1.0); horizontal flip
Validation preprocessing	Resize to 224 × 224
Normalization	ImageNet
Initialization	ImageNet-pretrained backbone weights
Loss function	Positive-class-weighted binary cross-entropy (BCEWithLogitsLoss with disease-specific pos_weight)
Positive-class weight derivation	Calculated separately for each disease from the training split only
Positive-class weight transformation	Log-scaled negative-to-positive ratio with clipping to 1–50
Focal loss	Not used
Oversampling / class resampling	Not used
Batch size	32
Optimizer	AdamW
Initial learning rate	1×10^{-4}
Weight decay	1×10^{-4}
Scheduler	Cosine annealing
Minimum learning rate	1×10^{-6}
Maximum epochs	100
Early-stopping monitor	Validation loss
Patience	10
min_delta	0
Checkpoint selection	Lowest validation loss

Note: Ten independent random seeds were used for each of the five architectures, resulting in 50 planned training runs. Disease-specific positive-class weights were calculated exclusively from the training split and were applied identically across architectures through the pos_weight argument of BCEWithLogitsLoss. The same positive-class-weighted loss was used to calculate validation loss for early stopping and checkpoint selection. Model checkpoints were selected according to the lowest validation loss, without reference to the held-out test set. No focal loss, oversampling, or other class-resampling strategy was used.

Class-Imbalance Handling

Because the NIH ChestX-ray14 dataset exhibits substantial disease-label imbalance, all five base architectures were trained using positive-class-weighted binary cross-entropy implemented with PyTorch BCEWithLogitsLoss. Disease-specific positive-class weights were calculated exclusively from the training split.

For disease d, let N_d^+ and N_d^- denote the numbers of positive and negative training samples, respectively. The raw negative-to-positive imbalance ratio was defined as

$$r_d = \frac{N_d^- + \epsilon}{N_d^+ + \epsilon},$$

where $\epsilon = 10^{-6}$ was included for numerical stability.

The disease-specific positive-class weight was then calculated as

$$w_d^+ = \text{clip}[\ln(1 + r_d), 1{,}50].$$

Equivalently,

$$w_d^+ = \text{clip}\left[\ln\left(1 + \frac{N_d^- + \epsilon}{N_d^+ + \epsilon}\right), 1{,}50\right], \qquad \epsilon = 10^{-6}.$$

The resulting disease-specific weights were supplied to the pos_weight argument of BCEWithLogitsLoss. The logarithmic transformation attenuated extremely large raw imbalance ratios, while clipping constrained the resulting positive-class weights to the range 1–50. This procedure increased the relative contribution of positive examples for less prevalent disease labels without directly resampling the training data. The same weighting procedure was applied consistently across all five base architectures. No focal loss, oversampling, undersampling, or other class-resampling strategy was used, and neither validation-set nor test-set labels were used to estimate the positive-class weights.

Training Computational Cost

To characterize the computational requirements of model development, training duration and stopping behavior were summarized across the 10 independently trained runs for each architecture. Training time reflects the complete optimization procedure up to the final early-stopping epoch.

Supplementary Table S3. Training computational cost

Architecture	Best epoch, median (range)	Final epoch, median (range)	Training time, mean ± SD (min/run)	Range (min/run)	Total GPU time (h)
DenseNet121	4 (3–5)	14 (13–15)	100.282 ± 15.916	82.01–123.69	16.714
ViT	23.5 (16–31)	33.5 (26–41)	243.690 ± 52.914	173.01–363.86	40.615
SwinV2	6 (5–6)	16 (15–16)	116.228 ± 15.032	100.37–142.81	19.371
DenseNet + ViT	5 (4–6)	15 (14–16)	134.121 ± 5.265	124.36–141.12	22.354
DenseNet + SwinV2	5 (4–6)	15 (14–16)	136.049 ± 4.187	131.37–141.18	22.675

Note: All 50 planned training runs were completed. The cumulative training time was 121.728 GPU-hours, with a mean runtime of 146.074 ± 56.698 min/run, a median runtime of 132.385 min/run, and a range of 82.01–363.86 min/run.

Benchmarking Protocol and Computing Environment

Computational efficiency was evaluated using a standardized benchmarking protocol across the five base architectures and the same 10 independently trained checkpoints per architecture. A fixed subset of 200 test images was used consistently across all architecture–seed combinations. The supplementary benchmarking materials document the preprocessing and timing protocol, hardware and software environment, inference latency, throughput, model complexity, and GPU memory requirements.

Supplementary Table S4. Inference benchmark configuration and computing environment

Category	Item	Setting
Benchmark design	Architectures	DenseNet121, ViT, SwinV2, DenseNet + ViT, DenseNet + SwinV2
	Random seeds per architecture	10
	Total evaluated checkpoints	50
	Benchmark subset	200 test images
	Subset random seed	42
Input	Preprocessing	Resize (256) + TenCrop (224) + ImageNet normalization
	Original-image batch size	1
	Crops per original image	10
Timing	Warm-up iterations	20
	Precision	FP32
	Checkpoint loading included	No
	CSV writing included	No
Hardware	Benchmark GPU	NVIDIA GeForce RTX 3090
	GPU memory	24 GB
	Selected device	1 × NVIDIA GeForce RTX 3090 (cuda:0)
	Visible GPUs	2 × NVIDIA GeForce RTX 3090
	DataParallel	No
	CPU	Intel Core i9-10900K
	System RAM	32 GB
Software	Operating system	Windows 11
	Python	3.9.13
	PyTorch	2.7.1+cu126
	torchvision	0.22.1+cu126
	timm	1.0.28
	CUDA runtime	12.6
	cuDNN	90701

Note: Each timing observation refers to one original chest radiograph processed using TenCrop inference (10 crops per original image). Timing measurements were obtained after 20 warm-up iterations. Checkpoint loading and result-file writing were excluded from the reported latency.

Architecture-Level Computational Efficiency

Using the standardized protocol described above, latency, throughput, parameter count, and peak GPU memory were evaluated separately for each architecture. Values were summarized across the 10 independently trained checkpoints of each architecture.

Supplementary Table S5. Computational efficiency of the five architectures

Architecture	Checkpoints (n)	Parameters (M)	Model inference latency, mean ± SD (ms/image)	Median latency (ms/image)	Throughput (images/s)	End-to-end latency, mean ± SD (ms/image)	Peak GPU memory (GB)
DenseNet121	10	6.97	27.06 ± 15.42	22.78	36.96	83.45 ± 125.07	0.135
ViT	10	85.81	22.97 ± 0.67	23.00	43.53	44.41 ± 1.06	0.404
SwinV2	10	48.94	38.19 ± 0.97	38.61	26.19	60.25 ± 2.16	0.343
DenseNet + ViT	10	93.94	44.80 ± 0.67	45.08	22.32	66.40 ± 2.23	0.460
DenseNet + SwinV2	10	58.09	61.36 ± 2.06	62.34	16.30	83.93 ± 3.28	0.376

Note: Latency values are summarized across 10 independently trained checkpoints per architecture. Model inference latency represents model-only forward inference after preprocessing, whereas end-to-end latency represents the measured preprocessing and inference pipeline for each original image. Throughput was calculated from the architecture-level mean model inference latency. Peak GPU memory represents the maximum allocated GPU memory observed during benchmarking.

Overall, the five architectures exhibited different trade-offs between model complexity and computational efficiency. ViT achieved the lowest mean model-only inference latency (22.97 ms/image) and the highest throughput (43.53 images/s), whereas DenseNet + SwinV2 showed the highest mean inference latency (61.36 ms/image). DenseNet121 had the smallest parameter count (6.97 M), while DenseNet + ViT had the largest parameter count (93.94 M). These results demonstrate that model parameter count and inference latency were not directly proportional across architectures.

Supplementary S3. Validation-Derived Analysis Provenance

To document separation between model development and held-out test evaluation, the provenance of all model-selection, thresholding, FAHP weighting, and seed-aggregation procedures was audited. All quantities used to construct or configure the ensemble were derived exclusively from the validation data and were locked before application to the test set.

Supplementary Table S6. Validation-derived analysis provenance and aggregation strategies

Part A. Provenance of validation-derived model selection, thresholds, and ensemble weights

Analysis component	Derivation dataset	Rule	Locked before test?	Test-set role
Best base architecture	Validation	Highest mean AUROC across 10 seeds	Yes	Evaluation only
Base seed threshold	Validation	Youden J	Yes	Fixed application
FAHP architecture weights	Validation	Sensitivity, specificity, precision + FAHP	Yes	Fixed application
Soft Voting seed threshold	Validation	Youden J	Yes	Fixed application
FAHP seed threshold	Validation	Youden J	Yes	Fixed application
Base-final threshold	Validation	10-seed aggregation + Youden J	Yes	Evaluation only
Soft-final threshold	Validation	10-seed aggregation + Youden J	Yes	Evaluation only
FAHP-final threshold	Validation	10-seed aggregation + Youden J	Yes	Evaluation only
ΔNB seed weights	Validation	Validation-derived ΔNB	Yes	Fixed application
FAHP–ΔNB final threshold	Validation	Weighted validation aggregation + Youden J	Yes	Evaluation only
CWO architecture weights	Validation	Disease-specific Brier-score minimization under non-negativity and sum-to-one constraints	Yes	Fixed application
CWO final threshold	Validation	CWO-aggregated validation probabilities + Youden J	Yes	Evaluation only

Part B. Summary of final aggregation strategies

Method	Architecture-level aggregation	Seed-level aggregation	Final threshold
**VAL-selected best base**	Single architecture selected separately for each disease according to the highest mean validation AUROC across 10 seeds	Equal probability averaging across the 10 seeds of the selected architecture	Validation-derived Youden J
**Hard Voting**	Within each seed, majority vote across five	Positive vote rate across the 10 seed-	Fixed at 0.5

Method	Architecture-level aggregation	Seed-level aggregation	Final threshold
	binary model decisions obtained using model- and disease-specific validation-derived thresholds	level majority-vote decisions	
**Soft Voting**	Within each seed, equal probability averaging across the five architectures	Equal probability averaging across the 10 seed-level Soft Voting outputs	Validation-derived Youden J
**FAHP**	Within each seed, disease-specific FAHP-weighted probability fusion across the five architectures	Equal probability averaging across the 10 seed-level FAHP outputs	Validation-derived Youden J
**FAHP–ΔNB**	Within each seed, disease-specific FAHP-weighted probability fusion across the five architectures	Validation-derived disease-specific ΔNB weighting across the 10 seed-level FAHP outputs	Validation-derived Youden J
**CWO**	Disease-specific Brier-optimized weighting across five architecture-level probabilities after equal averaging of the 10 seeds within each architecture	Equal contribution of the 10 seeds; implemented by averaging seeds within each architecture before applying the optimized CWO architecture weights	Validation-derived Youden J

Note: All model-selection procedures, architecture-selection procedures, ensemble weights, seed-aggregation weights, and operating thresholds were derived exclusively from the validation set and were fixed before held-out test evaluation. CWO architecture weights were optimized separately for each disease by minimizing the validation-set Brier score subject to non-negativity and sum-to-one constraints. For CWO, the 10 seed-specific probabilities were equally averaged within each architecture before optimization of the disease-specific architecture weights; because the same disease-specific architecture-weight vector is applied to all seed contributions, this is mathematically equivalent to applying the fixed CWO weights within each seed followed by equal averaging of the resulting seed-level ensemble probabilities. The held-out test set was used exclusively for final performance assessment.

Validation-set dependence

Although no test-set information was used during model development, the same validation partition was used for multiple development steps, including checkpoint selection, disease-specific threshold derivation, estimation of FAHP performance criteria and architecture weights, ΔNet Benefit-guided seed weighting, CWO architecture-weight optimization, and final ensemble threshold determination. Repeated use of a single validation partition may increase dependence on that sample and may contribute to validation overfitting, particularly for rare disease labels with limited positive examples. Future studies should consider nested validation, separate model-selection and ensemble-construction/calibration subsets, or resampling-based validation procedures to reduce this dependence.

Analysis Method Definitions and Aggregation Conventions

To ensure consistent interpretation of the supplementary analyses, the ensemble methods and aggregation levels used throughout this study are defined below. Two analysis levels were distinguished. **Seed-level analysis** refers to predictions or performance estimates obtained separately for each of the 10 random seeds. **Final-level analysis** refers to a single set of predictions obtained after aggregation across the 10 seed-level outputs. Unless otherwise stated, all model selection, ensemble weights, and disease-specific operating thresholds were derived exclusively from the validation set and were fixed before application to the held-out test set.

Validation-Selected Best Base Model

The **validation-selected best base model** was defined separately for each disease. Among the five base architectures—DenseNet121, ViT, SwinV2, DenseNet + ViT, and DenseNet + SwinV2—the architecture with the highest mean validation AUROC across the 10 random seeds was selected for that disease. The selected architecture was determined using validation data only and was not reselected according to test-set performance.

For final-level evaluation, the predictions from the 10 independently trained checkpoints of the selected architecture were aggregated to obtain a single disease-specific probability for each image. A final operating threshold was then determined from the corresponding aggregated validation probabilities by maximizing Youden's index and was subsequently applied unchanged to the held-out test set.

Hard Voting

Hard Voting was implemented as a thresholded decision-level ensemble. For each disease and random seed, the probability generated by each of the five base models was first converted into a binary prediction using that model's seed-specific and disease-specific Youden threshold derived from the validation set.

Within each seed, the five binary model predictions were combined by majority voting. A seed-level Hard Voting prediction was classified as positive when at least three of the five base models produced positive predictions.

For seed-level discrimination analyses, the proportion of positive model votes among the five base models, ranging from 0/5 to 5/5, was used as the score for AUROC and AUPRC calculation rather than a single binary majority-vote output.

For final-level analysis, the 10 seed-level Hard Voting decisions were aggregated as the proportion of seeds producing a positive majority-vote decision. This final positive-vote rate ranged from 0 to 1, and a fixed threshold of 0.5 was used to obtain the final binary classification.

Because Hard Voting produces a discrete vote-rate score rather than a continuously calibrated probability, its final-level Brier score was calculated using the positive-vote rate across the 10 seed-level Hard Voting outputs. Accordingly, the Hard Voting Brier score should be interpreted as the squared prediction error of the vote-rate score and not as a direct assessment of calibration of a continuous probabilistic model.

Soft Voting

Soft Voting was implemented as a two-stage equal-weight probability-level ensemble. Within each random seed, the disease-specific predicted probabilities from the five base architectures were averaged with equal weights to produce one seed-level Soft

Voting probability:

$$p_{i,s,d}^{\text{Soft}} = \frac{1}{K}\sum_{k=1}^{K} p_{i,s,d,k},$$

where $p_{i,s,d,k}$denotes the predicted probability for image i, random seed s, disease d, and base model k, and $K = 5$denotes the number of base architectures.

At the final level, the 10 seed-level Soft Voting probabilities were again averaged with equal weights:

$$p_{i,d}^{\text{Soft,final}} = \frac{1}{S}\sum_{s=1}^{S} p_{i,s,d}^{\text{Soft}},$$

where $S = 10$denotes the number of random seeds.

For each disease, the final Soft Voting threshold was derived from the aggregated validation probabilities by maximizing Youden's index and was subsequently applied unchanged to the corresponding aggregated test probabilities.

Soft Voting therefore served as the principal equal-weight probability-aggregation baseline, with equal weighting applied both across architectures and across random seeds.

FAHP Model-Weighted Fusion

FAHP refers to disease-specific probability-level model weighting based on the fuzzy analytic hierarchy process. For each disease, validation-set sensitivity, specificity, and precision were first summarized for each of the five base architectures across the 10 random seeds. These performance criteria were combined using the predefined FAHP criterion weights to derive a disease-specific score for each architecture. Following application of the model-weighting exponent γ, the resulting scores were normalized to obtain a disease-specific model-weight vector:

$$\alpha_d = (\alpha_{d,1}, \ldots, \alpha_{d,K}), \sum_{k=1}^{K} \alpha_{d,k} = 1.$$

The FAHP model weights were derived exclusively from validation-set information and were held fixed for the corresponding test-set predictions.

For each random seed, the FAHP-weighted probability for disease $d$was calculated as:

$$p_{i,s,d}^{\text{FAHP}} = \sum_{k=1}^{K} \alpha_{d,k}\, p_{i,s,d,k}.$$

In the **seed-level analyses**, FAHP therefore represents only the disease-specific model-weighting stage. Each of the 10 seeds yields one FAHP-weighted probability set, and seed-level performance is summarized across these 10 independently trained ensemble members.

In the **final-level FAHP ablation analysis**, the 10 seed-level FAHP probabilities were aggregated using equal seed weights:

$$p_{i,d}^{\text{FAHP,final}} = \frac{1}{S}\sum_{s=1}^{S} p_{i,s,d}^{\text{FAHP}}.$$

The final FAHP threshold for each disease was derived from the corresponding equally aggregated validation probabilities using Youden's index and was subsequently applied unchanged to the test set.

Accordingly, final-level FAHP isolates the contribution of disease-specific FAHP model weighting without incorporating ΔNet Benefit-guided seed weighting.

Detailed FAHP preference specifications, triangular fuzzy numbers, criterion weights, defuzzification procedures, and consistency assessments are provided in Supplementary S3.

FAHP–ΔNB

FAHP–ΔNB denotes the complete proposed two-stage ensemble framework. The first stage is identical to FAHP and performs disease-specific probability-level fusion of the five base architectures using validation-derived FAHP model weights.

In the second stage, the 10 seed-level FAHP predictions are aggregated using disease-specific weights derived from validation-set

ΔNet Benefit (ΔNB), rather than equal seed averaging.
For each disease and seed, a validation-based Youden threshold was first determined from the corresponding seed-level FAHP validation probabilities. Net benefit (NB) was evaluated at this validation-derived threshold, and ΔNet Benefit was calculated relative to the better of the treat-none and treat-all reference strategies, as defined in the main Methods.
The resulting validation-derived ΔNB values were transformed into non-negative seed weights and normalized across the 10 seeds:

$$\sum_{s=1}^{S} w_{s,d}^{\Delta NB} = 1.$$

The final FAHP–ΔNB probability was then calculated as:

$$p_{i,d}^{\text{FAHP}-\Delta\text{NB}} = \sum_{s=1}^{S} w_{s,d}^{\Delta NB}\, p_{i,s,d}^{\text{FAHP}}.$$

For each disease, a single final operating threshold was subsequently derived from the ΔNB-weighted aggregated validation probabilities using Youden's index and was applied unchanged to the corresponding held-out test probabilities.
Thus, the distinction between FAHP and FAHP–ΔNB is specifically the second-stage seed aggregation procedure:

- FAHP uses equal weighting across the 10 seed-level FAHP predictions.
- FAHP–ΔNB uses validation-derived, disease-specific ΔNB weighting across the 10 seed-level FAHP predictions.

FAHP–ΔNB therefore represents the complete proposed framework, whereas seed-level FAHP and final-level equal-seed FAHP are used as ablation analyses to isolate the contribution of the disease-specific model-weighting stage.
ΔNet Benefit was used in this study as an auxiliary validation-derived weighting signal rather than as a full decision-curve analysis framework or as a procedure for selecting clinically actionable probability thresholds.

Constrained Weight Optimization

Constrained Weight Optimization (CWO) was included as a conventional validation-based weighted-ensemble comparator to assess whether direct numerical optimization of model weights provided advantages relative to equal-weight Soft Voting and preference-based FAHP weighting. CWO was evaluated only at the final aggregation level and was not included in the seed-level robustness analysis. Equal contribution of the 10 random seeds was retained in the final CWO ensemble.
For each disease, predicted probabilities were first equally averaged across the 10 random seeds within each of the five base architectures. The resulting architecture-level validation probability was defined as

$$\bar{p}_{i,d,k}^{\text{VAL}} = \frac{1}{S}\sum_{s=1}^{S} p_{i,s,d,k}^{\text{VAL}},$$

where $p_{i,s,d,k}^{\text{VAL}}$ denotes the validation-set predicted probability for image i, seed s, disease d, and base architecture k, and $S = 10$denotes the number of random seeds.
This implementation retains equal contribution of the 10 seeds. Because a single disease-specific CWO architecture-weight vector was subsequently applied to the seed-averaged architecture probabilities, the resulting final probability is mathematically equivalent to applying the same fixed CWO architecture weights within each seed and then equally averaging the 10 resulting seed-level ensemble probabilities.
The five architecture-level probabilities were then combined using a disease-specific CWO weight vector:

$$p_{i,d}^{\text{CWO,VAL}} = \sum_{k=1}^{K} w_{d,k}^{\text{CWO}}\, \bar{p}_{i,d,k}^{\text{VAL}},$$

where $K = 5$ denotes the number of base architectures and $w_{d,k}^{\text{CWO}}$ denotes the optimized weight assigned to architecture $k$for disease d.

The weights were constrained to be non-negative and to sum to one:

$$w_{d,k}^{\mathrm{CWO}} \geq 0,$$

and

$$\sum_{k=1}^{K} w_{d,k}^{\mathrm{CWO}} = 1.$$

The disease-specific CWO weights were estimated exclusively from the validation set by minimizing the Brier score:

$$w_{d}^{\mathrm{CWO}} = \arg\min_{w_d} \frac{1}{N_d} \sum_{i=1}^{N_d} \left(\sum_{k=1}^{K} w_{d,k}\, \bar{p}_{i,d,k}^{\mathrm{VAL}} - y_{i,d} \right)^2 ,$$

where N_d denotes the number of validation images evaluated for disease d, $\bar{p}_{i,d,k}^{\mathrm{VAL}}$ is the 10-seed-averaged validation probability for architecture k, and $y_{i,d}$ is the corresponding binary reference label.

Optimization was performed using sequential least-squares programming (SLSQP), initialized with equal architecture weights $1/K$. If optimization failed or produced an invalid solution, equal weighting across the five architectures was used as a predefined fallback. The optimized disease-specific weights were then fixed before test-set evaluation.

For the held-out test set, probabilities were likewise first equally averaged across the 10 seeds within each architecture:

$$\bar{p}_{i,d,k}^{\mathrm{TEST}} = \frac{1}{S} \sum_{s=1}^{S} p_{i,s,d,k}^{\mathrm{TEST}} .$$

The validation-derived CWO weights were subsequently applied unchanged:

$$p_{i,d}^{\mathrm{CWO,TEST}} = \sum_{k=1}^{K} w_{d,k}^{\mathrm{CWO}}\, \bar{p}_{i,d,k}^{\mathrm{TEST}} .$$

For each disease, the final CWO operating threshold was determined from the corresponding CWO validation probabilities by maximizing Youden's index and was subsequently applied unchanged to the held-out test probabilities.

Unlike FAHP, which derives disease-specific architecture weights from multiple validation performance criteria and a predefined preference structure, CWO directly estimates the architecture-weight vector that minimizes validation-set probability prediction error. CWO was therefore included as a conventional optimization-based comparator and was not a component of the proposed FAHP–ΔNB framework.

Supplementary S4. FAHP Configuration and Two-Stage Weighting

This appendix provides supplementary methodological details and analyses related to fuzzy analytic hierarchy process (FAHP) preference specification and configurability. Three predefined illustrative preference configurations were examined: sensitivity-priority, specificity-priority, and balanced. The primary sensitivity-priority configuration was author-defined as an illustrative screening-oriented preference scenario. No formal preference elicitation, survey, Delphi process, or consensus procedure involving a clinician panel was conducted in the present study. Accordingly, the term clinician-configurable refers to the ability of the FAHP framework to incorporate user-defined or clinician-defined diagnostic preference structures rather than to a preference model empirically elicited from clinicians in this experiment. The same trained model checkpoints, original validation and test predictions, γ parameter, ΔNet Benefit (ΔNB) weighting procedure, and evaluation dataset were retained across configurations. Only the FAHP criterion preference specification was changed; consequently, preference-dependent criterion weights and downstream ensemble quantities were recalculated separately for each configuration.

FAHP criterion weighting and consistency assessment

Pairwise comparisons among sensitivity, specificity, and precision were represented using triangular fuzzy numbers (TFNs). For a TFN l, m, u, the reciprocal comparison was defined as:

$$(l, m, u)^{-1} = \left(\frac{1}{u}, \frac{1}{m}, \frac{1}{l}\right)$$

Fuzzy criterion weights were obtained using the fuzzy geometric-mean procedure. For a fuzzy weight $w_i = (l_i, m_i, u_i)$, defuzzification was performed using the arithmetic mean:

$$w_i^{crisp} = \frac{l_i + m_i + u_i}{3}$$

The resulting crisp weights were then normalized to sum to 1:

$$w_i = \frac{w_i^{\text{crisp}}}{\sum_{j=1}^{n} w_j^{\text{crisp}}}$$

Consistency was evaluated using the corresponding defuzzified crisp comparison matrix. The consistency index (CI) was calculated as:

$$CI = \frac{\lambda_{max} - n}{n - 1}$$

and the consistency ratio (CR) as:

$$CR = \frac{CI}{RI}$$

where $n = 3$ and $RI = 0.58$. A $CR < 0.10$ was considered acceptable. The term *balanced configuration* refers to equal FAHP criterion weighting of sensitivity, specificity, and precision and is distinct from *balanced accuracy*, which is a diagnostic performance metric.

For the primary sensitivity-priority configuration, the fuzzy pairwise-comparison matrix was

$$\tilde{\mathbf{A}}_{\text{Sens}} = \begin{bmatrix} (1,1,1) & (5,6,7) & (7,8,9) \\ \left(\frac{1}{7}, \frac{1}{6}, \frac{1}{5}\right) & (1,1,1) & (1,2,3) \\ \left(\frac{1}{9}, \frac{1}{8}, \frac{1}{7}\right) & \left(\frac{1}{3}, \frac{1}{2}, 1\right) & (1,1,1) \end{bmatrix}$$

where the rows and columns are ordered as sensitivity, specificity, and precision.

Supplementary Table S7. FAHP fuzzy linguistic scale, pairwise-comparison configurations, and consistency assessment

Part A. Five-level fuzzy linguistic scale

Level	Linguistic judgment	Triangular fuzzy number
1	Equal importance	(1, 1, 1)
2	Moderate importance	(1, 2, 3)
3	Strong importance	(3, 4, 5)
4	Very strong importance	(5, 6, 7)
5	Extreme importance	(7, 8, 9)

Part B. Pairwise-comparison configurations

Configuration	Pairwise judgments
Sensitivity-priority	Sensitivity > Specificity: (5, 6, 7); Sensitivity > Precision: (7, 8, 9); Specificity > Precision: (1, 2, 3)
Specificity-priority	Specificity > Sensitivity: (5, 6, 7); Specificity > Precision: (7, 8, 9); Sensitivity > Precision: (1, 2, 3)
Balanced	Sensitivity = Specificity = Precision: (1, 1, 1)

Part C. Final fuzzy criterion weights and consistency

Configuration	Criterion	Fuzzy weight TFN	Defuzzified normalized weight	λmax	CI	CR
Sensitivity-priority	Sensitivity	(0.6120, 0.7692, 0.9641)	0.7646	3.0913	0.0457	0.0787
	Specificity	(0.0978, 0.1468, 0.2044)	0.1464			
	Precision	(0.0624, 0.0840, 0.1267)	0.0890			
Specificity-priority	Sensitivity	(0.0978, 0.1468, 0.2044)	0.1464	3.0913	0.0457	0.0787
	Specificity	(0.6120, 0.7692, 0.9641)	0.7646			
	Precision	(0.0624, 0.0840, 0.1267)	0.0890			
Balanced	Sensitivity	(0.3333, 0.3333, 0.3333)	0.3333	3.0000	0.0000	0.0000
	Specificity	(0.3333, 0.3333, 0.3333)	0.3333			
	Precision	(0.3333, 0.3333, 0.3333)	0.3333			

Note: TFN, triangular fuzzy number; CI, consistency index; CR, consistency ratio. Criterion weights were derived using the FAHP procedure described above. The reported defuzzified weights were normalized to sum to 1 within each preference configuration. Consistency statistics are reported at the configuration level.

Disease-Specific FAHP Architecture Weights

Disease-specific FAHP architecture weights were derived exclusively from the validation set under the primary sensitivity-priority preference configuration. For each disease, validation-set sensitivity, specificity, and precision for the five base architectures were first normalized and combined using the predefined FAHP criterion weights. The resulting architecture scores were then transformed using the prespecified model-weighting exponent, γ, and normalized across the five architectures.

The resulting disease-specific weights, $\alpha_{d,k}$, determine the relative contribution of architecture k to the seed-level FAHP probability for disease d, with

$$\sum_{k=1}^{5} \alpha_{d,k} = 1$$

All FAHP architecture weights were derived from validation data only and were locked before evaluation on the held-out test set.

Supplementary Table S8. Disease-specific FAHP architecture weights under the primary sensitivity-priority configuration

Disease	DenseNet121	DenseNet + ViT	ViT	SwinV2	DenseNet + SwinV2
Atelectasis	0.1529	0.1605	0.2034	0.0290	**0.4543**
Cardiomegaly	0.1897	0.1316	0.1437	**0.4963**	0.0387
Effusion	0.2995	0.1800	0.0000	**0.3198**	0.2007
Infiltration	**0.6581**	0.1956	0.0109	0.0577	0.0776
Mass	0.1569	0.3458	0.0000	0.1130	**0.3843**
Nodule	0.2356	0.2684	0.0001	0.1954	**0.3006**
Pneumonia	**0.3732**	0.3719	0.0715	0.1655	0.0179
Pneumothorax	0.1600	0.2385	0.0000	0.1951	**0.4064**
Consolidation	0.0251	**0.3341**	0.0581	0.2679	0.3148
Edema	0.0385	0.1285	**0.4578**	0.1141	0.2611
Emphysema	0.1490	0.3166	0.0000	0.1985	**0.3359**
Fibrosis	0.2413	0.0279	0.2947	**0.2978**	0.1383
Pleural Thickening	**0.3710**	0.2574	0.0053	0.2409	0.1254
Hernia	0.0579	0.0253	0.0610	**0.7963**	0.0595

Note: Values are normalized disease-specific FAHP architecture weights ($\alpha_{d,k}$) derived exclusively from validation-set sensitivity, specificity, and precision. The primary sensitivity-priority FAHP criterion weights and the prespecified model-weighting exponent $\gamma = 2.0$ were used. Weights sum to 1 across the five architectures within each disease. Bold values indicate the largest architecture contribution within each disease and do not represent model selection. All weights were locked before held-out test-set evaluation.

ΔNet Benefit-Guided Seed Aggregation Weights

Following the disease-specific FAHP architecture weighting, a second aggregation stage was used to combine the 10 seed-level FAHP probability predictions. For each disease and random seed, ΔNet Benefit was calculated exclusively from validation-set predictions at the corresponding validation-derived FAHP operating threshold.

Under the primary configuration, positive validation-derived ΔNet Benefit values were transformed using the prespecified exponent $\gamma_{\mathrm{NB}} = 1.0$. Seeds with non-positive or non-finite ΔNet Benefit retained a small baseline weight of $\epsilon = 10^{-6}$. The resulting values were normalized across the 10 seeds to obtain the disease-specific seed aggregation weights, $w_{d,s}^{\Delta NB}$, such that

$$\sum_{s=1}^{S} w_{d,s}^{\Delta NB} = 1, \qquad S = 10$$

When all seed-specific ΔNet Benefit values for a disease were non-positive or non-finite, all seeds received the same baseline contribution before normalization, resulting in equal normalized aggregation weights of $1/S = 0.1$. These validation-derived seed weights were subsequently fixed and applied unchanged to the held-out test-set probabilities.

Supplementary Table S9. Disease-specific ΔNet Benefit-guided seed aggregation weights under the primary sensitivity-priority configuration

Disease	306	346	668	696	985	797	492	775	762	228
Atelectasis	0.0296	0.0781	0.1324	0.0866	0.1263	0.0869	0.1243	0.1158	0.1166	0.1034
Cardiomegaly	0.0776	<0.0001	<0.0001	0.2285	0.2346	0.0043	0.0310	0.1111	0.1468	0.1660
Effusion	0.0737	0.0875	0.1072	0.1072	0.1014	0.0862	0.0998	0.1100	0.1065	0.1205
Infiltration	0.0833	0.1330	0.0604	0.0821	0.1754	0.1754	0.0429	0.0723	0.1298	0.0454
Mass	0.0746	0.0950	0.0302	0.0846	0.1628	0.1113	0.1103	0.1139	0.0982	0.1191
Nodule	0.1000	0.1000	0.1000	0.1000	0.1000	0.1000	0.1000	0.1000	0.1000	0.1000
Pneumonia	0.1000	0.1000	0.1000	0.1000	0.1000	0.1000	0.1000	0.1000	0.1000	0.1000
Pneumothorax	0.1016	0.1115	0.0848	0.0941	0.1124	0.1012	0.0993	0.0970	0.1008	0.0974
Consolidation	0.1000	0.1000	0.1000	0.1000	0.1000	0.1000	0.1000	0.1000	0.1000	0.1000
Edema	0.2327	<0.0001	0.1155	0.2292	0.2408	<0.0001	<0.0001	0.0470	<0.0001	0.1345
Emphysema	0.0879	0.2350	0.0418	0.0748	0.1227	0.0490	0.1799	0.0268	0.0782	0.1039
Fibrosis	0.1000	0.1000	0.1000	0.1000	0.1000	0.1000	0.1000	0.1000	0.1000	0.1000
Pleural Thickening	0.0007	0.0007	0.0007	0.7648	0.2297	0.0007	0.0007	0.0007	0.0007	0.0007
Hernia	0.1439	0.1244	0.1086	0.1692	0.1268	0.1569	0.0602	<0.0001	0.0920	0.0178

Note: Values represent disease-specific normalized seed aggregation weights used in the final FAHP–ΔNB ensemble. For each disease d and random seed s, ΔNet Benefit (ΔNB) was calculated exclusively from validation-set predictions at the corresponding validation-derived FAHP operating threshold. Under the primary configuration, the unnormalized seed-weighting quantity was defined as $u_{d,s}(\gamma_{\mathrm{NB}}) = \epsilon + \left(\Delta NB_{d,s}\right)^{\gamma_{\mathrm{NB}}}$ for positive ΔNB values and $u_{d,s}(\gamma_{\mathrm{NB}}) = \epsilon$ for non-positive or non-finite values, with $\gamma_{\mathrm{NB}} = 1.0$ and $\epsilon = 10^{-6}$. The resulting quantities were normalized across the 10 seeds to obtain $w_{d,s}^{\Delta NB}$, with $\sum_{s=1}^{S} w_{d,s}^{\Delta NB} = 1$. When all seed-specific ΔNB values for a disease were non-positive or non-finite, equal baseline contributions resulted in normalized seed weights of $1/S = 0.1$. All seed aggregation weights were derived exclusively from validation data and were locked before held-out test-set evaluation. Values reported as <0.0001 denote very small positive normalized weights rather than exact zeros.

Final-level performance summarization

For the final-level preference analysis, the 10 seed-level predictions were aggregated to obtain a single final prediction for each disease and preference configuration. Performance was calculated separately for each of the 14 disease labels and summarized using an unweighted macro-average:

$$\overline{M}_{macro} = \frac{1}{14}\sum_{d=1}^{14} M_d$$

where M_d denotes the disease-specific value of a given performance metric for disease d.

Balanced accuracy (BA) was defined as:

$$BA = \frac{\text{Sensitivity} + \text{Specificity}}{2}$$

For each preference configuration, disease-specific final decision thresholds were derived from the corresponding aggregated validation probabilities by maximizing Youden's index and were subsequently applied unchanged to the held-out test set.

The Brier score for disease d was calculated as:

$$BS_d = \frac{1}{N_d}\sum_{i=1}^{N_d} (p_{id} - y_{id})^2$$

where N_d denotes the number of evaluated samples for disease d, p_{id} is the final predicted probability for sample i and disease d, and y_{id} is the corresponding binary reference label. Disease-specific Brier scores were subsequently summarized using the same unweighted macro-averaging procedure across the 14 disease labels. The Brier score is threshold-independent, with lower values indicating lower probability prediction error.

Supplementary S5. Preference and Hyperparameter Robustness

Seed-level robustness analysis

In addition to the final FAHP–ΔNB analysis, preference-dependent performance was examined at the seed level before ΔNB-guided seed aggregation. For each disease, FAHP model weights were derived from validation-set sensitivity, specificity, and precision summarized across the 10 random seeds and were then applied to the corresponding seed-level model predictions. For each seed, performance metrics were calculated separately for the 14 disease labels and macro-averaged across diseases. The resulting seed-level values were subsequently summarized across the 10 random seeds. This analysis therefore evaluates the robustness of the disease-specific FAHP model-weighting stage rather than the complete FAHP–ΔNB framework.

Supplementary Table S10. Seed-level performance of FAHP weighted fusion under alternative preference configurations

Configuration	AUROC	AUPRC	Sensitivity	Specificity	Precision	F1 score	Balanced accuracy
Sensitivity-priority	0.850 ± 0.001	0.287 ± 0.004	0.759 ± 0.008	0.789 ± 0.009	0.148 ± 0.004	0.233 ± 0.005	0.774 ± 0.002
Specificity-priority	0.851 ± 0.001	0.294 ± 0.003	0.754 ± 0.014	0.794 ± 0.012	0.151 ± 0.004	0.236 ± 0.005	0.774 ± 0.002
Balanced	0.852 ± 0.001	0.295 ± 0.003	0.759 ± 0.013	0.791 ± 0.011	0.150 ± 0.004	0.235 ± 0.005	0.775 ± 0.003

Note: Values are presented as mean ± standard deviation across 10 random seeds. This table reports seed-level performance after disease-specific FAHP model weighting and before final ΔNB-guided seed aggregation.

Disease-specific operating characteristics

To examine the effect of alternative FAHP preference specifications at the individual disease level, final sensitivity, specificity, and balanced accuracy were evaluated separately for each of the 14 disease labels. The preference configurations specify the relative importance assigned to sensitivity, specificity, and precision during FAHP model weighting; they do not impose a deterministic constraint requiring the prioritized metric to be maximized for every disease. Because preference-dependent FAHP model weighting is followed by ΔNB-guided seed aggregation and configuration-specific validation-derived final thresholding, changes in disease-specific operating characteristics may vary across disease labels.

Supplementary S6. Statistical Robustness and Patient-Cluster Bootstrap Uncertainty

Two complementary levels of statistical analysis were used to characterize distinct sources of uncertainty. Seed-level nonparametric analyses were used to quantify variability associated with random initialization and stochastic model training, whereas disease-stratified patient-cluster bootstrap analyses were used to quantify sampling uncertainty in the held-out test cohort while preserving the dependence among multiple radiographs from the same patient. These two sources of variability were therefore evaluated and interpreted separately.

Seed-level nonparametric analysis

For each disease and each prespecified principal performance endpoint, the 10 random seeds were treated as paired repeated measurements across eight methods: DenseNet121, ViT, SwinV2, DenseNet + ViT, DenseNet + SwinV2, Hard Voting, Soft Voting, and FAHP weighted fusion under the primary sensitivity-priority configuration.

Overall differences among the eight methods were evaluated separately for each disease and performance endpoint using the Friedman test. With $K = 8$ methods, each Friedman test had $K - 1 = 7$ degrees of freedom.

Pairwise method comparisons were subsequently evaluated using two-sided Wilcoxon signed-rank tests based on the paired seed-level results. To account for multiple pairwise comparisons, the resulting Wilcoxon P values were adjusted using the Benjamini–Hochberg false discovery rate procedure within each disease-by-metric family across the full set of tested method pairs. Friedman omnibus P values were reported without multiplicity adjustment.

The corresponding focused seed-level pairwise comparisons of FAHP with Hard Voting, Soft Voting, and the validation-selected best base model, together with Benjamini–Hochberg-adjusted P values, are reported in Table 3 of the main manuscript. The

adjusted *P* values reported in Table 3 were derived from the disease-by-metric correction families described above rather than from correction restricted only to the displayed focused comparisons.

AUROC, AUPRC, balanced accuracy, and F1 score were prespecified as the principal seed-level comparative endpoints. CWO was not included in these seed-level nonparametric comparisons because CWO was defined and evaluated only at the final aggregation level.

Importantly, the 10 random seeds represent repeated model-training realizations rather than independent patient samples. Accordingly, the seed-level analyses quantify training-related stochastic variability and should not be interpreted as confidence intervals or inferential uncertainty for the underlying patient population.

Supplementary Table S11. Friedman omnibus test results for seed-level method comparisons under the primary sensitivity-priority FAHP configuration

Disease	AUROC χ^2	P value	AUPRC χ^2	P value	Balanced accuracy χ^2	P value	F1 score χ^2	P value
Atelectasis	65.17	<0.0001	64.20	<0.0001	51.67	<0.0001	32.27	<0.0001
Cardiomegaly	60.57	<0.0001	61.80	<0.0001	54.17	<0.0001	23.80	0.0012
Effusion	61.57	<0.0001	61.13	<0.0001	59.53	<0.0001	34.93	<0.0001
Infiltration	60.73	<0.0001	57.73	<0.0001	48.87	<0.0001	39.97	<0.0001
Mass	62.90	<0.0001	61.63	<0.0001	57.77	<0.0001	29.10	0.0001
Nodule	61.30	<0.0001	64.60	<0.0001	57.73	<0.0001	35.23	<0.0001
Pneumonia	56.87	<0.0001	58.27	<0.0001	35.33	<0.0001	22.73	0.0019
Pneumothorax	63.33	<0.0001	60.57	<0.0001	61.77	<0.0001	30.67	<0.0001
Consolidation	62.13	<0.0001	62.00	<0.0001	33.40	<0.0001	7.13	0.4151
Edema	61.20	<0.0001	61.37	<0.0001	28.50	0.0002	15.40	0.0312
Emphysema	61.27	<0.0001	59.67	<0.0001	50.43	<0.0001	31.87	<0.0001
Fibrosis	60.40	<0.0001	62.83	<0.0001	46.53	<0.0001	21.07	0.0037
Pleural Thickening	60.70	<0.0001	62.63	<0.0001	46.40	<0.0001	35.40	<0.0001
Hernia	56.03	<0.0001	60.57	<0.0001	43.13	<0.0001	39.30	<0.0001

Note: Friedman omnibus tests were performed separately for each disease and principal performance endpoint using the 10 random seeds as paired repeated measurements across eight methods: DenseNet121, ViT, SwinV2, DenseNet + ViT, DenseNet + SwinV2, Hard Voting, Soft Voting, and FAHP weighted fusion. With eight methods, each Friedman test had 7 degrees of freedom. AUROC, AUPRC, balanced accuracy, and F1 score were treated as the principal comparative endpoints. Friedman *P* values are unadjusted omnibus-test *P* values. Subsequent pairwise comparisons were performed using two-sided Wilcoxon signed-rank tests, with *P* values adjusted using the Benjamini–Hochberg false discovery rate procedure within each disease-by-metric family. CWO was not included because it was defined only at the final aggregation level. Seed-level analyses characterize variability across stochastic model-training realizations and should not be interpreted as patient-population uncertainty.

Disease-stratified patient-cluster bootstrap analysis

To quantify sampling uncertainty in the held-out test population while accounting for the presence of multiple radiographs from the same patient, a disease-stratified patient-cluster bootstrap analysis was performed. The patient, rather than the individual radiograph, was used as the resampling unit. The held-out test set comprised 6,187 patients and 22,433 radiographs.

For each of the 14 disease labels separately, a patient was classified as disease-positive if at least one test radiograph from that patient carried the corresponding disease label and as disease-negative otherwise. Because NIH ChestX-ray14 is a multi-label dataset, this positive/negative stratification was constructed independently for each disease rather than according to the complete 14-label combination. Consequently, the same patient could belong to the positive stratum for more than one disease across the corresponding disease-specific analyses.

For disease d, let P_d and N_d denote the numbers of disease-positive and disease-negative patients, respectively. Therefore,

$$P_d + N_d = N_{patient} = 6{,}187.$$

For each bootstrap replicate, P_d patients were sampled with replacement from the disease-positive patient stratum and N_d patients were sampled with replacement from the disease-negative patient stratum. Thus, every bootstrap replicate contained 6,187 patient-cluster draws while preserving the original disease-specific patient-level positive-to-negative composition of the held-out test cohort.

Because sampling was performed with replacement, a patient could be selected zero, one, or multiple times within a replicate, and the number of unique patients represented in an individual bootstrap replicate could therefore be smaller than 6,187.

Whenever a patient was selected, all test radiographs belonging to that patient were retained as a single cluster. If the same patient was sampled multiple times, the complete set of radiographs belonging to that patient was repeated accordingly. This procedure preserved the within-patient dependence among repeated radiographs while maintaining the disease-specific patient-level class composition.

A total of

$$B = 2{,}000$$

bootstrap replicates were generated separately for each of the 14 disease labels. A fixed random seed of 20260825 was used for reproducibility. Within each disease-specific bootstrap replicate, the identical resampled patient clusters were applied to all comparator methods, thereby preserving the paired structure required for method-to-method comparisons.

Model training, validation-selected architecture selection, disease-specific FAHP architecture weights, validation-derived ΔNet Benefit seed weights, CWO architecture weights, and validation-derived operating thresholds were not re-estimated during bootstrap resampling. All such quantities had been determined before held-out test evaluation and remained fixed throughout the bootstrap analysis.

For each bootstrap replicate, performance metrics were recalculated from the final test predictions associated with the resampled patient clusters. The reported bootstrap endpoints were AUROC, AUPRC, balanced accuracy, F1 score, and Brier score.

For disease d, method m, performance metric M, and bootstrap replicate b, let

$$M_{d,m}^{(b)}$$

denote the corresponding bootstrap estimate.

For the method-specific uncertainty estimates reported in Supplementary Table S12, percentile-based 95% confidence intervals were obtained from the empirical distribution of the 2,000 bootstrap estimates:

$$CI_{95\%}(M_{d,m}) = \left[Q_{0.025}\left(\left\{M_{d,m}^{(b)}\right\}_{b=1}^{B}\right),\ Q_{0.975}\left(\left\{M_{d,m}^{(b)}\right\}_{b=1}^{B}\right)\right]$$

where $Q_q(\cdot)$ denotes the empirical q-quantile and $B = 2{,}000$.

Paired Patient-Cluster Bootstrap Comparisons

For the paired method comparisons reported in Supplementary Table S13, FAHP–ΔNB and the comparator method were evaluated using the identical resampled patient clusters within every bootstrap replicate.

For disease d, comparator method c, performance metric M, and bootstrap replicate b, the paired difference was defined as

$$\Delta M_{d,c}^{(b)} = M_{d,\text{FAHP}-\Delta\text{NB}}^{(b)} - M_{d,c}^{(b)}$$

The corresponding point difference reported in Supplementary Table S13 was defined analogously from the observed final-level

test-set metrics:

$$\Delta M_{d,c} = M_{d,\mathrm{FAHP-\Delta NB}} - M_{d,c}$$

The percentile-based 95% confidence interval for the paired difference was obtained from the empirical distribution of the 2,000 paired bootstrap differences:

$$CI_{95\%}(\Delta M_{d,c}) = \left[Q_{0.025}\left(\left\{\Delta M_{d,c}^{(b)}\right\}_{b=1}^{B}\right),\ Q_{0.975}\left(\left\{\Delta M_{d,c}^{(b)}\right\}_{b=1}^{B}\right)\right]$$

For AUROC, AUPRC, balanced accuracy, and F1 score,

$$\Delta M_{d,c} > 0$$

indicates a higher value for FAHP–ΔNB than for the comparator. For Brier score,

$$\Delta M_{d,c} < 0$$

favors FAHP–ΔNB because lower Brier scores indicate lower squared probability prediction error.

A paired bootstrap 95% confidence interval excluding zero was interpreted as indicating that the direction of the observed difference was stable under patient-cluster resampling. These intervals quantify sampling uncertainty associated with the held-out patient population and are distinct from the seed-level analyses used to characterize stochastic variability arising from model training.

The patient-cluster bootstrap confidence intervals were not multiplicity-adjusted simultaneous confidence intervals. Therefore, bootstrap intervals excluding zero were interpreted descriptively as paired uncertainty estimates rather than as multiplicity-controlled confirmatory hypothesis tests. Multiplicity adjustment was applied separately to the seed-level Wilcoxon comparisons as described above.

Supplementary Table S12. Disease-stratified patient-cluster bootstrap confidence intervals for final-level performance under the primary sensitivity-priority FAHP configuration

Disease	Method	AUROC (95% CI)	AUPRC (95% CI)	Balanced accuracy (95% CI)	F1 score (95% CI)	Brier score (95% CI)
Atelectasis	VAL-selected best base	0.8344 (0.8246 to 0.8446)	0.3845 (0.3610 to 0.4090)	0.7578 (0.7474 to 0.7689)	0.3737 (0.3570 to 0.3902)	0.08654 (0.08294 to 0.08995)
	Hard Voting	0.7911 (0.7804 to 0.8017)	0.2590 (0.2432 to 0.2746)	0.7573 (0.7465 to 0.7679)	0.3841 (0.3669 to 0.4011)	0.24761 (0.23222 to 0.26312)
	Soft Voting	0.8327 (0.8232 to 0.8428)	0.3828 (0.3590 to 0.4076)	0.7569 (0.7462 to 0.7677)	0.3807 (0.3639 to 0.3975)	0.08473 (0.08131 to 0.08801)
	CWO	0.8331 (0.8236 to 0.8429)	0.3865 (0.3627 to 0.4119)	0.7540 (0.7434 to 0.7643)	0.3852 (0.3682 to 0.4022)	0.08167 (0.07829 to 0.08494)
	FAHP	0.8327 (0.8230 to 0.8428)	0.3819 (0.3587 to 0.4060)	0.7548 (0.7436 to 0.7660)	0.3896 (0.3720 to 0.4076)	0.08584 (0.08239 to 0.08917)
	FAHP–ΔNB	0.8325 (0.8227 to 0.8426)	0.3818 (0.3586 to 0.4060)	0.7561 (0.7452 to 0.7672)	0.3903 (0.3726 to 0.4083)	0.08553 (0.08211 to 0.08886)
Cardiomegaly	VAL-selected best base	0.9186 (0.9043 to 0.9303)	0.3251 (0.2734 to 0.3779)	0.8404 (0.8210 to 0.8578)	0.2134 (0.1872 to 0.2419)	0.02559 (0.02264 to 0.02872)
	Hard Voting	0.8917 (0.8749 to 0.9052)	0.1518 (0.1288 to 0.1770)	0.8490 (0.8310 to 0.8642)	0.2094 (0.1847 to 0.2369)	0.14168 (0.12849 to 0.15662)
	Soft Voting	0.9227 (0.9088 to	0.3278 (0.2769 to	0.8482 (0.8302 to	0.2067 (0.1819 to	0.02569 (0.02287 to

Disease	Method	AUROC (95% CI)	AUPRC (95% CI)	Balanced accuracy (95% CI)	F1 score (95% CI)	Brier score (95% CI)
		0.9343)	0.3788)	0.8636)	0.2344)	0.02867)
	CWO	0.9113 (0.8963 to 0.9241)	0.2921 (0.2423 to 0.3428)	0.8360 (0.8171 to 0.8530)	0.2011 (0.1761 to 0.2290)	0.02496 (0.02217 to 0.02804)
	FAHP	0.9222 (0.9083 to 0.9336)	0.3282 (0.2782 to 0.3794)	0.8484 (0.8302 to 0.8644)	0.2148 (0.1894 to 0.2432)	0.02556 (0.02272 to 0.02859)
	FAHP–ΔNB	0.9227 (0.9090 to 0.9340)	0.3274 (0.2771 to 0.3783)	0.8524 (0.8349 to 0.8668)	0.2070 (0.1823 to 0.2349)	0.02423 (0.02149 to 0.02713)
Effusion	VAL-selected best base	0.8866 (0.8774 to 0.8949)	0.5381 (0.5080 to 0.5685)	0.8116 (0.8017 to 0.8216)	0.5147 (0.4940 to 0.5365)	0.08257 (0.07720 to 0.08845)
	Hard Voting	0.8414 (0.8320 to 0.8506)	0.3701 (0.3493 to 0.3922)	0.8125 (0.8027 to 0.8221)	0.5032 (0.4826 to 0.5251)	0.18236 (0.16862 to 0.19696)
	Soft Voting	0.8887 (0.8794 to 0.8969)	0.5436 (0.5125 to 0.5734)	0.8131 (0.8037 to 0.8227)	0.4933 (0.4726 to 0.5141)	0.08063 (0.07549 to 0.08638)
	CWO	0.8884 (0.8790 to 0.8965)	0.5424 (0.5119 to 0.5714)	0.8122 (0.8022 to 0.8220)	0.4963 (0.4760 to 0.5178)	0.08000 (0.07493 to 0.08572)
	FAHP	0.8892 (0.8800 to 0.8974)	0.5447 (0.5133 to 0.5753)	0.8145 (0.8049 to 0.8239)	0.5069 (0.4861 to 0.5284)	0.08101 (0.07571 to 0.08692)
	FAHP–ΔNB	0.8893 (0.8801 to 0.8974)	0.5447 (0.5134 to 0.5751)	0.8123 (0.8025 to 0.8216)	0.4825 (0.4618 to 0.5036)	0.08085 (0.07556 to 0.08672)
Infiltration	VAL-selected best base	0.7140 (0.7010 to 0.7259)	0.3512 (0.3256 to 0.3758)	0.6531 (0.6399 to 0.6654)	0.4063 (0.3827 to 0.4285)	0.13903 (0.13482 to 0.14308)
	Hard Voting	0.6819 (0.6682 to 0.6946)	0.2927 (0.2722 to 0.3123)	0.6573 (0.6442 to 0.6697)	0.4123 (0.3888 to 0.4346)	0.25312 (0.24050 to 0.26586)
	Soft Voting	0.7198 (0.7072 to 0.7313)	0.3551 (0.3304 to 0.3793)	0.6579 (0.6454 to 0.6701)	0.4102 (0.3878 to 0.4316)	0.13797 (0.13387 to 0.14194)
	CWO	0.7201 (0.7075 to 0.7315)	0.3554 (0.3302 to 0.3795)	0.6571 (0.6435 to 0.6694)	0.4136 (0.3902 to 0.4359)	0.13737 (0.13329 to 0.14134)
	FAHP	0.7199 (0.7075 to 0.7312)	0.3540 (0.3296 to 0.3783)	0.6573 (0.6456 to 0.6683)	0.4045 (0.3832 to 0.4250)	0.13855 (0.13428 to 0.14252)
	FAHP–ΔNB	0.7195 (0.7071 to 0.7309)	0.3537 (0.3290 to 0.3777)	0.6578 (0.6453 to 0.6689)	0.4059 (0.3843 to 0.4267)	0.13763 (0.13341 to 0.14160)
Mass	VAL-selected best base	0.8652 (0.8492 to 0.8801)	0.3253 (0.2869 to 0.3664)	0.7877 (0.7690 to 0.8051)	0.2926 (0.2639 to 0.3242)	0.04796 (0.04483 to 0.05157)
	Hard Voting	0.8217 (0.8025 to 0.8402)	0.2055 (0.1767 to 0.2380)	0.7814 (0.7612 to 0.8008)	0.3070 (0.2756 to 0.3403)	0.13696 (0.12551 to 0.15004)
	Soft Voting	0.8631 (0.8463 to 0.8789)	0.3237 (0.2846 to 0.3664)	0.7797 (0.7601 to 0.7989)	0.3114 (0.2793 to 0.3455)	0.04662 (0.04365 to 0.05007)
	CWO	0.8626 (0.8462 to 0.8788)	0.3255 (0.2853 to 0.3694)	0.7689 (0.7473 to 0.7897)	0.3208 (0.2857 to 0.3556)	0.04494 (0.04196 to 0.04850)
	FAHP	0.8675 (0.8516 to	0.3274 (0.2888 to	0.7911 (0.7730 to	0.2926 (0.2636 to	0.04693 (0.04387 to

Disease	Method	AUROC (95% CI)	AUPRC (95% CI)	Balanced accuracy (95% CI)	F1 score (95% CI)	Brier score (95% CI)
		0.8826)	0.3683)	0.8080)	0.3246)	0.05046)
	FAHP–ΔNB	0.8676 (0.8516 to 0.8825)	0.3272 (0.2888 to 0.3679)	0.7915 (0.7737 to 0.8087)	0.2915 (0.2624 to 0.3231)	0.04637 (0.04334 to 0.04989)
Nodule	VAL-selected best base	0.8029 (0.7845 to 0.8190)	0.2838 (0.2490 to 0.3184)	0.7301 (0.7114 to 0.7465)	0.2603 (0.2376 to 0.2834)	0.05816 (0.05492 to 0.06146)
	Hard Voting	0.7634 (0.7430 to 0.7806)	0.1798 (0.1571 to 0.2020)	0.7232 (0.7047 to 0.7396)	0.2730 (0.2485 to 0.2965)	0.16767 (0.15629 to 0.17970)
	Soft Voting	0.7990 (0.7799 to 0.8154)	0.2812 (0.2466 to 0.3160)	0.7241 (0.7053 to 0.7403)	0.2811 (0.2563 to 0.3057)	0.05776 (0.05461 to 0.06091)
	CWO	0.7977 (0.7786 to 0.8141)	0.2785 (0.2433 to 0.3137)	0.7174 (0.6968 to 0.7349)	0.2940 (0.2655 to 0.3203)	0.05651 (0.05339 to 0.05970)
	FAHP	0.8036 (0.7848 to 0.8195)	0.2879 (0.2526 to 0.3225)	0.7229 (0.7041 to 0.7393)	0.2431 (0.2222 to 0.2655)	0.05770 (0.05446 to 0.06093)
	FAHP–ΔNB	0.8036 (0.7848 to 0.8195)	0.2879 (0.2526 to 0.3225)	0.7229 (0.7041 to 0.7393)	0.2431 (0.2222 to 0.2655)	0.05770 (0.05446 to 0.06093)
Pneumonia	VAL-selected best base	0.7729 (0.7417 to 0.8026)	0.0538 (0.0416 to 0.0778)	0.6976 (0.6666 to 0.7291)	0.0577 (0.0490 to 0.0672)	0.01281 (0.01195 to 0.01378)
	Hard Voting	0.7414 (0.7105 to 0.7724)	0.0299 (0.0247 to 0.0360)	0.7099 (0.6789 to 0.7398)	0.0585 (0.0505 to 0.0673)	0.18501 (0.16708 to 0.20327)
	Soft Voting	0.7781 (0.7491 to 0.8058)	0.0533 (0.0410 to 0.0747)	0.7047 (0.6762 to 0.7320)	0.0471 (0.0409 to 0.0541)	0.01295 (0.01210 to 0.01389)
	CWO	0.7626 (0.7327 to 0.7908)	0.0453 (0.0360 to 0.0653)	0.6907 (0.6614 to 0.7189)	0.0446 (0.0387 to 0.0508)	0.01259 (0.01176 to 0.01352)
	FAHP	0.7790 (0.7501 to 0.8073)	0.0537 (0.0408 to 0.0750)	0.7081 (0.6780 to 0.7389)	0.0553 (0.0479 to 0.0634)	0.01319 (0.01232 to 0.01416)
	FAHP–ΔNB	0.7790 (0.7501 to 0.8073)	0.0537 (0.0408 to 0.0750)	0.7081 (0.6780 to 0.7389)	0.0553 (0.0479 to 0.0634)	0.01319 (0.01232 to 0.01416)
Pneumothorax	VAL-selected best base	0.8906 (0.8705 to 0.9062)	0.3669 (0.3029 to 0.4255)	0.8212 (0.8001 to 0.8386)	0.3071 (0.2698 to 0.3439)	0.04061 (0.03673 to 0.04459)
	Hard Voting	0.8577 (0.8359 to 0.8750)	0.2241 (0.1881 to 0.2586)	0.8199 (0.7975 to 0.8377)	0.3133 (0.2753 to 0.3504)	0.14615 (0.13432 to 0.15754)
	Soft Voting	0.8935 (0.8741 to 0.9089)	0.3700 (0.3092 to 0.4261)	0.8192 (0.7961 to 0.8373)	0.3222 (0.2832 to 0.3603)	0.04026 (0.03649 to 0.04408)
	CWO	0.8945 (0.8753 to 0.9097)	0.3729 (0.3109 to 0.4287)	0.8262 (0.8040 to 0.8433)	0.3204 (0.2826 to 0.3576)	0.04014 (0.03634 to 0.04402)
	FAHP	0.8941 (0.8745 to 0.9095)	0.3725 (0.3097 to 0.4309)	0.8236 (0.8005 to 0.8417)	0.3257 (0.2870 to 0.3629)	0.04044 (0.03659 to 0.04434)
	FAHP–ΔNB	0.8941 (0.8745 to 0.9095)	0.3727 (0.3097 to 0.4309)	0.8247 (0.8020 to 0.8422)	0.3250 (0.2863 to 0.3621)	0.04043 (0.03658 to 0.04433)
Consolidation	VAL-selected	0.8188 (0.8040 to	0.1776 (0.1495 to	0.7437 (0.7294 to	0.1754 (0.1577 to	0.04380 (0.04057 to

Disease	Method	AUROC (95% CI)	AUPRC (95% CI)	Balanced accuracy (95% CI)	F1 score (95% CI)	Brier score (95% CI)
	best base	0.8333)	0.2087)	0.7570)	0.1937)	0.04741)
	Hard Voting	0.7732 (0.7567 to 0.7887)	0.1069 (0.0934 to 0.1207)	0.7434 (0.7275 to 0.7589)	0.1856 (0.1668 to 0.2049)	0.26532 (0.24707 to 0.28507)
	Soft Voting	0.8189 (0.8040 to 0.8335)	0.1767 (0.1511 to 0.2052)	0.7436 (0.7279 to 0.7588)	0.1807 (0.1626 to 0.1995)	0.04428 (0.04094 to 0.04798)
	CWO	0.8179 (0.8028 to 0.8324)	0.1744 (0.1500 to 0.2011)	0.7441 (0.7295 to 0.7586)	0.1758 (0.1584 to 0.1940)	0.04371 (0.04041 to 0.04734)
	FAHP	0.8192 (0.8045 to 0.8334)	0.1755 (0.1499 to 0.2035)	0.7452 (0.7296 to 0.7601)	0.1799 (0.1620 to 0.1980)	0.04416 (0.04085 to 0.04783)
	FAHP–ΔNB	0.8192 (0.8045 to 0.8334)	0.1755 (0.1499 to 0.2035)	0.7452 (0.7296 to 0.7601)	0.1799 (0.1620 to 0.1980)	0.04416 (0.04085 to 0.04783)
Edema	VAL-selected best base	0.9019 (0.8868 to 0.9156)	0.1677 (0.1346 to 0.2162)	0.8276 (0.8057 to 0.8472)	0.1379 (0.1226 to 0.1548)	0.02275 (0.02059 to 0.02511)
	Hard Voting	0.8664 (0.8487 to 0.8826)	0.0869 (0.0752 to 0.0999)	0.8295 (0.8090 to 0.8479)	0.1449 (0.1288 to 0.1625)	0.16164 (0.14275 to 0.18063)
	Soft Voting	0.9063 (0.8927 to 0.9189)	0.1755 (0.1399 to 0.2281)	0.8327 (0.8145 to 0.8498)	0.1355 (0.1209 to 0.1519)	0.02203 (0.01991 to 0.02429)
	CWO	0.9043 (0.8911 to 0.9166)	0.1703 (0.1363 to 0.2204)	0.8143 (0.7917 to 0.8349)	0.1538 (0.1359 to 0.1727)	0.02058 (0.01869 to 0.02261)
	FAHP	0.9066 (0.8933 to 0.9188)	0.1742 (0.1396 to 0.2258)	0.8349 (0.8167 to 0.8519)	0.1380 (0.1233 to 0.1546)	0.02120 (0.01922 to 0.02331)
	FAHP–ΔNB	0.9064 (0.8933 to 0.9185)	0.1745 (0.1398 to 0.2261)	0.8342 (0.8164 to 0.8506)	0.1332 (0.1191 to 0.1492)	0.02051 (0.01860 to 0.02251)
Emphysema	VAL-selected best base	0.9407 (0.9274 to 0.9525)	0.4258 (0.3364 to 0.5111)	0.8603 (0.8439 to 0.8744)	0.1833 (0.1570 to 0.2125)	0.01833 (0.01643 to 0.02018)
	Hard Voting	0.9088 (0.8920 to 0.9231)	0.1725 (0.1425 to 0.2072)	0.8663 (0.8479 to 0.8826)	0.2119 (0.1820 to 0.2441)	0.11995 (0.10867 to 0.13250)
	Soft Voting	0.9435 (0.9308 to 0.9545)	0.4139 (0.3263 to 0.5035)	0.8711 (0.8504 to 0.8890)	0.2501 (0.2150 to 0.2876)	0.01906 (0.01724 to 0.02088)
	CWO	0.9400 (0.9268 to 0.9518)	0.4206 (0.3305 to 0.5086)	0.8556 (0.8397 to 0.8698)	0.1740 (0.1489 to 0.2021)	0.01838 (0.01652 to 0.02018)
	FAHP	0.9452 (0.9324 to 0.9561)	0.4212 (0.3330 to 0.5113)	0.8725 (0.8534 to 0.8892)	0.2350 (0.2023 to 0.2697)	0.01915 (0.01722 to 0.02103)
	FAHP–ΔNB	0.9454 (0.9328 to 0.9561)	0.4213 (0.3335 to 0.5116)	0.8741 (0.8529 to 0.8922)	0.2780 (0.2402 to 0.3176)	0.01888 (0.01695 to 0.02078)
Fibrosis	VAL-selected best base	0.8439 (0.8205 to 0.8655)	0.1485 (0.1119 to 0.1920)	0.7667 (0.7397 to 0.7922)	0.1049 (0.0927 to 0.1174)	0.01757 (0.01639 to 0.01886)
	Hard Voting	0.8124 (0.7866 to 0.8364)	0.0614 (0.0519 to 0.0716)	0.7614 (0.7356 to 0.7863)	0.0938 (0.0833 to 0.1043)	0.19444 (0.18344 to 0.20585)
	Soft Voting	0.8484 (0.8247 to	0.1554 (0.1172 to	0.7583 (0.7350 to	0.0802 (0.0717 to	0.01672 (0.01561 to

Disease	Method	AUROC (95% CI)	AUPRC (95% CI)	Balanced accuracy (95% CI)	F1 score (95% CI)	Brier score (95% CI)
		0.8700)	0.1996)	0.7790)	0.0888)	0.01796)
	CWO	0.8452 (0.8203 to 0.8677)	0.1489 (0.1115 to 0.1920)	0.7629 (0.7415 to 0.7836)	0.0821 (0.0735 to 0.0911)	0.01641 (0.01531 to 0.01762)
	FAHP	0.8464 (0.8222 to 0.8682)	0.1540 (0.1157 to 0.1993)	0.7541 (0.7309 to 0.7761)	0.0783 (0.0700 to 0.0868)	0.01685 (0.01573 to 0.01808)
	FAHP–ΔNB	0.8464 (0.8222 to 0.8682)	0.1540 (0.1157 to 0.1993)	0.7541 (0.7309 to 0.7761)	0.0783 (0.0700 to 0.0868)	0.01685 (0.01573 to 0.01808)
Pleural Thickening	VAL-selected best base	0.8033 (0.7835 to 0.8211)	0.1576 (0.1333 to 0.1869)	0.7227 (0.7003 to 0.7428)	0.1707 (0.1524 to 0.1891)	0.03417 (0.03164 to 0.03683)
	Hard Voting	0.7690 (0.7471 to 0.7881)	0.0968 (0.0830 to 0.1116)	0.7292 (0.7074 to 0.7484)	0.1668 (0.1491 to 0.1846)	0.18679 (0.17373 to 0.20129)
	Soft Voting	0.7995 (0.7788 to 0.8179)	0.1543 (0.1303 to 0.1837)	0.7220 (0.6991 to 0.7417)	0.1763 (0.1574 to 0.1957)	0.03410 (0.03165 to 0.03670)
	CWO	0.7989 (0.7782 to 0.8178)	0.1515 (0.1279 to 0.1808)	0.7325 (0.7127 to 0.7505)	0.1534 (0.1380 to 0.1693)	0.03380 (0.03125 to 0.03652)
	FAHP	0.8026 (0.7821 to 0.8206)	0.1595 (0.1347 to 0.1894)	0.7262 (0.7038 to 0.7458)	0.1736 (0.1555 to 0.1921)	0.03402 (0.03152 to 0.03667)
	FAHP–ΔNB	0.8007 (0.7803 to 0.8188)	0.1543 (0.1305 to 0.1834)	0.7270 (0.7056 to 0.7459)	0.1617 (0.1450 to 0.1785)	0.03389 (0.03139 to 0.03651)
Hernia	VAL-selected best base	0.9625 (0.9302 to 0.9847)	0.4674 (0.2737 to 0.6461)	0.8724 (0.7892 to 0.9350)	0.0665 (0.0432 to 0.0956)	0.00138 (0.00108 to 0.00169)
	Hard Voting	0.8965 (0.8149 to 0.9568)	0.0484 (0.0237 to 0.0797)	0.8658 (0.7865 to 0.9205)	0.0374 (0.0246 to 0.0530)	0.05530 (0.05000 to 0.06079)
	Soft Voting	0.9499 (0.9085 to 0.9788)	0.4816 (0.2829 to 0.6605)	0.8477 (0.7722 to 0.9027)	0.0221 (0.0148 to 0.0311)	0.00138 (0.00112 to 0.00168)
	CWO	0.9485 (0.9055 to 0.9783)	0.4786 (0.2817 to 0.6564)	0.8790 (0.8030 to 0.9337)	0.0397 (0.0268 to 0.0559)	0.00133 (0.00107 to 0.00162)
	FAHP	0.9370 (0.8832 to 0.9753)	0.4295 (0.2348 to 0.6133)	0.8512 (0.7723 to 0.9061)	0.0275 (0.0180 to 0.0389)	0.00151 (0.00124 to 0.00191)
	FAHP–ΔNB	0.9423 (0.8917 to 0.9779)	0.4520 (0.2611 to 0.6346)	0.8654 (0.7899 to 0.9203)	0.0295 (0.0198 to 0.0414)	0.00145 (0.00117 to 0.00185)

Note: Values are final-level point estimates with 95% empirical percentile confidence intervals obtained from 2,000 disease-stratified patient-cluster bootstrap replicates. Patients were the resampling units, and all test radiographs belonging to a sampled patient were retained together. Positive and negative patient clusters were resampled separately for each disease while preserving the original disease-specific patient-level class composition. The same resampled patient clusters were used for all methods within each replicate. Validation-selected architectures, FAHP weights, ΔNet Benefit-derived seed weights, CWO weights, and operating thresholds remained fixed throughout bootstrap evaluation. These confidence intervals quantify held-out patient-population sampling uncertainty and are distinct from seed-level training variability. For Hard Voting, the reported Brier score is based on the final positive-vote rate and should be interpreted as squared prediction error of the vote-rate score rather than calibration of a continuous model-derived probability.

Supplementary Table S13. Paired disease-stratified patient-cluster bootstrap comparisons of FAHP–ΔNB with final-level comparator methods

Disease	Comparator	ΔAUROC (95% CI)	ΔAUPRC (95% CI)	Δbalanced accuracy (95% CI)	ΔF1 (95% CI)	ΔBrier (95% CI)
Atelectasis	VAL-selected best base	**-0.0020 (-0.0032 to -0.0008)**	-0.0028 (-0.0063 to +0.0009)	-0.0017 (-0.0070 to +0.0034)	**+0.0166 (+0.0115 to +0.0217)**	**-0.00102 (-0.00139 to -0.00063)**
	Hard Voting	**+0.0413 (+0.0374 to +0.0456)**	**+0.1228 (+0.1073 to +0.1401)**	-0.0012 (-0.0050 to +0.0025)	**+0.0062 (+0.0028 to +0.0096)**	**-0.16208 (-0.17611 to -0.14877)**
	Soft Voting	-0.0002 (-0.0008 to +0.0003)	-0.0011 (-0.0030 to +0.0009)	-0.0008 (-0.0044 to +0.0028)	**+0.0096 (+0.0064 to +0.0129)**	**+0.00079 (+0.00063 to +0.00095)**
	CWO	-0.0006 (-0.0027 to +0.0016)	-0.0048 (-0.0119 to +0.0020)	+0.0021 (-0.0032 to +0.0074)	**+0.0051 (+0.0002 to +0.0102)**	**+0.00386 (+0.00325 to +0.00445)**
	FAHP	**-0.0002 (-0.0004 to -0.0000)**	-0.0001 (-0.0007 to +0.0004)	+0.0013 (-0.0000 to +0.0028)	+0.0007 (-0.0007 to +0.0019)	**-0.00031 (-0.00037 to -0.00025)**
Cardiomegaly	VAL-selected best base	**+0.0041 (+0.0018 to +0.0064)**	+0.0022 (-0.0043 to +0.0086)	**+0.0120 (+0.0022 to +0.0224)**	**-0.0064 (-0.0116 to -0.0010)**	**-0.00135 (-0.00166 to -0.00108)**
	Hard Voting	**+0.0310 (+0.0250 to +0.0368)**	**+0.1756 (+0.1397 to +0.2148)**	+0.0034 (-0.0026 to +0.0102)	-0.0024 (-0.0060 to +0.0014)	**-0.11744 (-0.13065 to -0.10608)**
	Soft Voting	-0.0000 (-0.0014 to +0.0016)	-0.0005 (-0.0055 to +0.0040)	+0.0041 (-0.0018 to +0.0107)	+0.0003 (-0.0034 to +0.0040)	**-0.00145 (-0.00168 to -0.00124)**
	CWO	**+0.0113 (+0.0071 to +0.0158)**	**+0.0353 (+0.0240 to +0.0487)**	**+0.0164 (+0.0053 to +0.0287)**	+0.0059 (-0.0005 to +0.0123)	**-0.00073 (-0.00126 to -0.00027)**
	FAHP	+0.0004 (-0.0005 to +0.0014)	-0.0008 (-0.0038 to +0.0014)	+0.0039 (-0.0022 to +0.0108)	**-0.0078 (-0.0113 to -0.0045)**	**-0.00133 (-0.00154 to -0.00115)**
Effusion	VAL-selected best base	**+0.0026 (+0.0017 to +0.0036)**	**+0.0066 (+0.0028 to +0.0104)**	+0.0007 (-0.0042 to +0.0062)	**-0.0322 (-0.0387 to -0.0259)**	**-0.00172 (-0.00211 to -0.00130)**
	Hard Voting	**+0.0478 (+0.0436 to +0.0519)**	**+0.1746 (+0.1560 to +0.1919)**	-0.0002 (-0.0038 to +0.0039)	**-0.0208 (-0.0251 to -0.0163)**	**-0.10152 (-0.11124 to -0.09205)**
	Soft Voting	**+0.0006 (+0.0001 to +0.0011)**	+0.0011 (-0.0010 to +0.0034)	-0.0008 (-0.0036 to +0.0020)	**-0.0108 (-0.0142 to -0.0077)**	+0.00021 (-0.00004 to +0.00047)
	CWO	**+0.0009 (+0.0001 to +0.0017)**	+0.0023 (-0.0008 to +0.0053)	+0.0001 (-0.0030 to +0.0036)	**-0.0138 (-0.0175 to -0.0100)**	**+0.00085 (+0.00046 to +0.00133)**
	FAHP	+0.0000 (-0.0000 to +0.0001)	-0.0000 (-0.0002 to +0.0002)	-0.0022 (-0.0059 to +0.0020)	**-0.0244 (-0.0292 to -0.0197)**	**-0.00016 (-0.00019 to -0.00014)**
Infiltration	VAL-selected best base	**+0.0056 (+0.0025 to +0.0088)**	+0.0025 (-0.0026 to +0.0079)	+0.0047 (-0.0007 to +0.0101)	-0.0004 (-0.0067 to +0.0060)	**-0.00140 (-0.00192 to -0.00087)**
	Hard Voting	**+0.0377 (+0.0332 to +0.0423)**	**+0.0610 (+0.0516 to +0.0723)**	+0.0005 (-0.0040 to +0.0050)	**-0.0064 (-0.0117 to -0.0011)**	**-0.11549 (-0.12506 to -0.10581)**
	Soft Voting	-0.0003 (-0.0016 to +0.0010)	-0.0014 (-0.0042 to +0.0016)	-0.0001 (-0.0038 to +0.0038)	**-0.0043 (-0.0085 to -0.0001)**	**-0.00034 (-0.00059 to -0.00008)**
	CWO	-0.0006 (-0.0024 to +0.0011)	-0.0017 (-0.0052 to +0.0022)	+0.0007 (-0.0044 to +0.0057)	**-0.0076 (-0.0136 to -0.0018)**	+0.00026 (-0.00006 to +0.00058)
	FAHP	-0.0003 (-0.0008 to +0.0001)	-0.0003 (-0.0011 to +0.0004)	+0.0005 (-0.0017 to +0.0025)	+0.0015 (-0.0009 to +0.0036)	**-0.00092 (-0.00102 to -0.00082)**

Disease	Comparator	ΔAUROC (95% CI)	ΔAUPRC (95% CI)	Δbalanced accuracy (95% CI)	ΔF1 (95% CI)	ΔBrier (95% CI)
Mass	VAL-selected best base	**+0.0024 (+0.0008 to +0.0041)**	+0.0018 (-0.0015 to +0.0051)	+0.0038 (-0.0006 to +0.0083)	-0.0010 (-0.0048 to +0.0027)	**-0.00160 (-0.00193 to -0.00129)**
	Hard Voting	**+0.0459 (+0.0394 to +0.0519)**	**+0.1216 (+0.1029 to +0.1421)**	**+0.0101 (+0.0024 to +0.0185)**	**-0.0154 (-0.0216 to -0.0089)**	**-0.09059 (-0.10195 to -0.08070)**
	Soft Voting	**+0.0044 (+0.0023 to +0.0067)**	+0.0034 (-0.0012 to +0.0083)	**+0.0118 (+0.0047 to +0.0195)**	**-0.0198 (-0.0262 to -0.0131)**	-0.00025 (-0.00055 to +0.00004)
	CWO	**+0.0049 (+0.0006 to +0.0094)**	+0.0017 (-0.0072 to +0.0108)	**+0.0226 (+0.0115 to +0.0343)**	**-0.0292 (-0.0401 to -0.0181)**	**+0.00142 (+0.00089 to +0.00197)**
	FAHP	+0.0001 (-0.0003 to +0.0005)	-0.0003 (-0.0010 to +0.0005)	+0.0004 (-0.0013 to +0.0024)	-0.0011 (-0.0025 to +0.0004)	**-0.00056 (-0.00068 to -0.00045)**
Nodule	VAL-selected best base	+0.0007 (-0.0012 to +0.0027)	**+0.0042 (+0.0009 to +0.0074)**	**-0.0071 (-0.0118 to -0.0022)**	**-0.0172 (-0.0210 to -0.0134)**	**-0.00046 (-0.00065 to -0.00028)**
	Hard Voting	**+0.0402 (+0.0341 to +0.0465)**	**+0.1081 (+0.0885 to +0.1290)**	-0.0003 (-0.0073 to +0.0072)	**-0.0298 (-0.0361 to -0.0236)**	**-0.10997 (-0.12004 to -0.10058)**
	Soft Voting	**+0.0046 (+0.0027 to +0.0066)**	**+0.0067 (+0.0023 to +0.0112)**	-0.0011 (-0.0084 to +0.0062)	**-0.0380 (-0.0450 to -0.0311)**	-0.00006 (-0.00029 to +0.00019)
	CWO	**+0.0059 (+0.0024 to +0.0097)**	**+0.0094 (+0.0041 to +0.0150)**	+0.0055 (-0.0035 to +0.0151)	**-0.0508 (-0.0612 to -0.0397)**	**+0.00119 (+0.00081 to +0.00156)**
	FAHP	0.0000 (0.0000 to 0.0000)	0.0000 (0.0000 to 0.0000)	0.0000 (0.0000 to 0.0000)	0.0000 (0.0000 to 0.0000)	0.00000 (0.00000 to 0.00000)
Pneumonia	VAL-selected best base	+0.0061 (-0.0022 to +0.0149)	-0.0001 (-0.0111 to +0.0075)	+0.0104 (-0.0041 to +0.0272)	-0.0023 (-0.0055 to +0.0008)	**+0.00038 (+0.00025 to +0.00051)**
	Hard Voting	**+0.0376 (+0.0245 to +0.0517)**	**+0.0238 (+0.0136 to +0.0414)**	-0.0018 (-0.0088 to +0.0065)	**-0.0032 (-0.0047 to -0.0016)**	**-0.17182 (-0.18944 to -0.15434)**
	Soft Voting	+0.0009 (-0.0021 to +0.0038)	+0.0004 (-0.0023 to +0.0034)	+0.0034 (-0.0142 to +0.0182)	**+0.0082 (+0.0052 to +0.0111)**	**+0.00024 (+0.00019 to +0.00030)**
	CWO	**+0.0164 (+0.0035 to +0.0290)**	+0.0083 (-0.0025 to +0.0193)	+0.0173 (-0.0027 to +0.0366)	**+0.0108 (+0.0073 to +0.0143)**	**+0.00059 (+0.00039 to +0.00080)**
	FAHP	0.0000 (0.0000 to 0.0000)	0.0000 (0.0000 to 0.0000)	0.0000 (0.0000 to 0.0000)	0.0000 (0.0000 to 0.0000)	0.00000 (0.00000 to 0.00000)
Pneumothorax	VAL-selected best base	**+0.0035 (+0.0018 to +0.0055)**	**+0.0058 (+0.0005 to +0.0114)**	+0.0035 (-0.0017 to +0.0079)	**+0.0179 (+0.0133 to +0.0222)**	-0.00018 (-0.00039 to +0.00000)
	Hard Voting	**+0.0364 (+0.0312 to +0.0421)**	**+0.1486 (+0.1112 to +0.1843)**	+0.0048 (-0.0001 to +0.0096)	**+0.0117 (+0.0074 to +0.0160)**	**-0.10572 (-0.11467 to -0.09660)**
	Soft Voting	+0.0006 (-0.0009 to +0.0021)	+0.0027 (-0.0040 to +0.0086)	**+0.0055 (+0.0007 to +0.0109)**	+0.0028 (-0.0014 to +0.0072)	+0.00017 (-0.00004 to +0.00038)
	CWO	-0.0004 (-0.0017 to +0.0008)	-0.0002 (-0.0050 to +0.0046)	-0.0015 (-0.0054 to +0.0025)	**+0.0046 (+0.0011 to +0.0084)**	**+0.00028 (+0.00015 to +0.00042)**
	FAHP	+0.0000 (-0.0001 to +0.0001)	+0.0002 (-0.0004 to +0.0008)	+0.0011 (-0.0005 to +0.0031)	-0.0007 (-0.0022 to +0.0008)	**-0.00001 (-0.00002 to -0.00000)**

**Disease**	**Comparator**	**ΔAUROC (95% CI)**	**ΔAUPRC (95% CI)**	**Δbalanced accuracy (95% CI)**	**ΔF1 (95% CI)**	**ΔBrier (95% CI)**
Consolidation	VAL-selected best base	+0.0003 (-0.0023 to +0.0030)	-0.0020 (-0.0097 to +0.0043)	+0.0015 (-0.0058 to +0.0084)	**+0.0045 (+0.0014 to +0.0075)**	**+0.00036 (+0.00014 to +0.00059)**
	Hard Voting	**+0.0460 (+0.0394 to +0.0525)**	**+0.0687 (+0.0516 to +0.0893)**	+0.0017 (-0.0037 to +0.0076)	**-0.0057 (-0.0083 to -0.0031)**	**-0.22116 (-0.23818 to -0.20520)**
	Soft Voting	+0.0002 (-0.0008 to +0.0013)	-0.0012 (-0.0045 to +0.0016)	+0.0016 (-0.0024 to +0.0058)	-0.0009 (-0.0028 to +0.0010)	**-0.00012 (-0.00021 to -0.00003)**
	CWO	+0.0012 (-0.0001 to +0.0026)	+0.0011 (-0.0034 to +0.0062)	+0.0011 (-0.0038 to +0.0054)	**+0.0041 (+0.0019 to +0.0062)**	**+0.00045 (+0.00030 to +0.00059)**
	FAHP	0.0000 (0.0000 to 0.0000)	0.0000 (0.0000 to 0.0000)	0.0000 (0.0000 to 0.0000)	0.0000 (0.0000 to 0.0000)	0.00000 (0.00000 to 0.00000)
Edema	VAL-selected best base	**+0.0045 (+0.0013 to +0.0082)**	+0.0067 (-0.0027 to +0.0178)	+0.0066 (-0.0043 to +0.0183)	**-0.0047 (-0.0081 to -0.0014)**	**-0.00224 (-0.00279 to -0.00173)**
	Hard Voting	**+0.0400 (+0.0320 to +0.0488)**	**+0.0876 (+0.0591 to +0.1326)**	+0.0047 (-0.0049 to +0.0156)	**-0.0117 (-0.0155 to -0.0080)**	**-0.14113 (-0.15849 to -0.12378)**
	Soft Voting	+0.0001 (-0.0014 to +0.0015)	-0.0011 (-0.0059 to +0.0040)	+0.0014 (-0.0043 to +0.0075)	**-0.0024 (-0.0043 to -0.0004)**	**-0.00152 (-0.00186 to -0.00118)**
	CWO	**+0.0021 (+0.0002 to +0.0041)**	+0.0042 (-0.0018 to +0.0101)	**+0.0199 (+0.0067 to +0.0340)**	**-0.0207 (-0.0270 to -0.0152)**	-0.00007 (-0.00023 to +0.00011)
	FAHP	-0.0002 (-0.0013 to +0.0008)	+0.0002 (-0.0028 to +0.0033)	-0.0007 (-0.0049 to +0.0047)	**-0.0048 (-0.0066 to -0.0029)**	**-0.00069 (-0.00082 to -0.00056)**
Emphysema	VAL-selected best base	**+0.0047 (+0.0011 to +0.0088)**	-0.0044 (-0.0172 to +0.0091)	**+0.0138 (+0.0003 to +0.0261)**	**+0.0946 (+0.0788 to +0.1093)**	**+0.00055 (+0.00020 to +0.00090)**
	Hard Voting	**+0.0367 (+0.0305 to +0.0437)**	**+0.2488 (+0.1847 to +0.3132)**	+0.0078 (-0.0016 to +0.0159)	**+0.0661 (+0.0554 to +0.0767)**	**-0.10107 (-0.11277 to -0.09075)**
	Soft Voting	**+0.0019 (+0.0000 to +0.0039)**	**+0.0074 (+0.0005 to +0.0151)**	+0.0030 (-0.0036 to +0.0086)	**+0.0279 (+0.0222 to +0.0339)**	-0.00018 (-0.00039 to +0.00006)
	CWO	**+0.0054 (+0.0016 to +0.0096)**	+0.0008 (-0.0105 to +0.0136)	**+0.0185 (+0.0048 to +0.0312)**	**+0.1040 (+0.0877 to +0.1200)**	**+0.00050 (+0.00017 to +0.00085)**
	FAHP	+0.0002 (-0.0004 to +0.0009)	+0.0002 (-0.0019 to +0.0022)	+0.0016 (-0.0062 to +0.0084)	**+0.0430 (+0.0349 to +0.0510)**	**-0.00027 (-0.00032 to -0.00021)**
Fibrosis	VAL-selected best base	+0.0025 (-0.0027 to +0.0083)	+0.0055 (-0.0017 to +0.0138)	-0.0126 (-0.0283 to +0.0054)	**-0.0266 (-0.0324 to -0.0209)**	**-0.00072 (-0.00089 to -0.00056)**
	Hard Voting	**+0.0340 (+0.0252 to +0.0431)**	**+0.0926 (+0.0601 to +0.1327)**	-0.0073 (-0.0186 to +0.0057)	**-0.0155 (-0.0189 to -0.0117)**	**-0.17759 (-0.18832 to -0.16703)**
	Soft Voting	-0.0020 (-0.0039 to +0.0000)	-0.0014 (-0.0055 to +0.0040)	-0.0041 (-0.0125 to +0.0045)	**-0.0019 (-0.0035 to -0.0002)**	**+0.00014 (+0.00009 to +0.00018)**
	CWO	+0.0012 (-0.0035 to +0.0062)	+0.0051 (-0.0048 to +0.0187)	-0.0088 (-0.0199 to +0.0029)	**-0.0038 (-0.0061 to -0.0014)**	**+0.00044 (+0.00032 to +0.00055)**
	FAHP	0.0000 (0.0000 to 0.0000)	0.0000 (0.0000 to 0.0000)	0.0000 (0.0000 to 0.0000)	0.0000 (0.0000 to 0.0000)	0.00000 (0.00000 to 0.00000)

Disease	Comparator	ΔAUROC (95% CI)	ΔAUPRC (95% CI)	Δbalanced accuracy (95% CI)	ΔF1 (95% CI)	ΔBrier (95% CI)
Pleural Thickening	VAL-selected best base	-0.0027 (-0.0072 to +0.0018)	-0.0033 (-0.0098 to +0.0033)	+0.0042 (-0.0060 to +0.0146)	**-0.0089 (-0.0145 to -0.0033)**	**-0.00028 (-0.00056 to -0.00002)**
	Hard Voting	**+0.0317 (+0.0242 to +0.0402)**	**+0.0575 (+0.0400 to +0.0811)**	-0.0022 (-0.0098 to +0.0057)	**-0.0051 (-0.0090 to -0.0009)**	**-0.15290 (-0.16573 to -0.14129)**
	Soft Voting	+0.0012 (-0.0026 to +0.0051)	+0.0000 (-0.0053 to +0.0046)	+0.0050 (-0.0037 to +0.0135)	**-0.0146 (-0.0200 to -0.0093)**	-0.00021 (-0.00044 to +0.00002)
	CWO	+0.0018 (-0.0036 to +0.0069)	+0.0029 (-0.0052 to +0.0104)	-0.0055 (-0.0157 to +0.0036)	**+0.0084 (+0.0036 to +0.0130)**	+0.00008 (-0.00015 to +0.00034)
	FAHP	-0.0019 (-0.0050 to +0.0012)	**-0.0051 (-0.0101 to -0.0011)**	+0.0008 (-0.0067 to +0.0090)	**-0.0119 (-0.0163 to -0.0075)**	-0.00013 (-0.00031 to +0.00005)
Hernia	VAL-selected best base	-0.0202 (-0.0526 to +0.0039)	-0.0154 (-0.0775 to +0.0364)	-0.0070 (-0.0322 to +0.0350)	**-0.0370 (-0.0541 to -0.0224)**	+0.00008 (-0.00017 to +0.00035)
	Hard Voting	**+0.0458 (+0.0169 to +0.0856)**	**+0.4036 (+0.2320 to +0.5666)**	-0.0005 (-0.0135 to +0.0297)	**-0.0079 (-0.0120 to -0.0039)**	**-0.05384 (-0.05936 to -0.04859)**
	Soft Voting	-0.0075 (-0.0242 to +0.0038)	-0.0296 (-0.0758 to +0.0074)	**+0.0176 (+0.0158 to +0.0196)**	**+0.0074 (+0.0049 to +0.0103)**	+0.00008 (-0.00003 to +0.00021)
	CWO	-0.0062 (-0.0251 to +0.0107)	-0.0266 (-0.0839 to +0.0174)	**-0.0136 (-0.0156 to -0.0117)**	**-0.0102 (-0.0146 to -0.0066)**	+0.00012 (-0.00005 to +0.00032)
	FAHP	**+0.0053 (+0.0020 to +0.0101)**	+0.0225 (-0.0107 to +0.0601)	**+0.0141 (+0.0015 to +0.0443)**	**+0.0020 (+0.0007 to +0.0040)**	**-0.00006 (-0.00009 to -0.00003)**

Note: All paired differences were defined as FAHP–ΔNB minus the comparator method. Values are observed point differences with 95% empirical percentile confidence intervals based on 2,000 paired disease-stratified patient-cluster bootstrap replicates. Within each bootstrap replicate, identical resampled patient clusters were used for FAHP–ΔNB and the corresponding comparator method. For AUROC, AUPRC, balanced accuracy, and F1 score, positive differences favor FAHP–ΔNB; for Brier score, negative differences favor FAHP–ΔNB because lower values indicate lower probability prediction error. Boldface indicates a paired 95% bootstrap confidence interval that excludes zero. These intervals quantify patient-level sampling uncertainty and are not multiplicity-adjusted simultaneous confidence intervals; accordingly, they should be interpreted as descriptive paired uncertainty estimates rather than multiplicity-controlled confirmatory hypothesis tests.

Supplementary S7. Probability Calibration, Ensemble Ablation, and Conventional Weighting Comparison

Probability calibration assessment

In addition to discrimination and threshold-dependent performance measures, probability calibration was evaluated for the principal final-level probability-based ensemble methods on the held-out test set. The analysis included Soft Voting, Constrained Weight Optimization (CWO), FAHP, and FAHP–ΔNB. Calibration was assessed using the Brier score, calibration intercept and slope, and disease-specific reliability curves.

For disease d and method m, the Brier score was calculated directly from the final continuous predicted probabilities as

$$\text{Brier}_{d,m} = \frac{1}{N_d}\sum_{i=1}^{N_d}\left(\hat{p}_{i,d,m} - y_{i,d}\right)^2$$

where $\hat{p}_{i,d,m}$ denotes the final predicted probability for image i, disease d, and method m, $y_{i,d}$ denotes the corresponding binary reference label, and N_d denotes the number of evaluated images for disease d. Because the Brier score is calculated directly from the predicted probabilities, it is independent of the validation-derived operating threshold. Lower values indicate

lower squared probability prediction error.

Calibration intercept and slope were estimated separately for each disease–method combination using the logistic recalibration model

$$\mathrm{logit}\left[P\left(Y_{i,d} = 1\right)\right] = a_{d,m} + b_{d,m}\,\mathrm{logit}\left(\hat{p}_{i,d,m}\right)$$

where $a_{d,m}$ is the calibration intercept and $b_{d,m}$ is the calibration slope. Ideal calibration corresponds to

$$a_{d,m} = 0 \qquad \text{and} \qquad b_{d,m} = 1$$

The calibration intercept and slope were interpreted jointly with the disease-specific reliability curves. Ideal calibration corresponds to an intercept of 0 and a slope of 1. A calibration slope below 1 indicates overly dispersed or overly extreme predictions, whereas a slope above 1 indicates insufficient dispersion of the predicted log-odds. Systematic over- or underprediction was assessed descriptively using the reliability curves together with the fitted calibration coefficients.

For numerical stability, logit-transformed predicted probabilities were internally standardized during model fitting, and the fitted coefficients were subsequently back-transformed to the original logit-probability scale.

Disease-specific reliability curves were constructed using 10 quantile-based probability bins. Within each bin, the mean predicted probability was plotted against the corresponding observed event proportion. The identity line represents ideal calibration. Curves lying predominantly below the identity line indicate that predicted probabilities tend to exceed the observed event proportions, whereas curves lying predominantly above the identity line indicate underprediction.

All calibration quantities were estimated on the held-out test set strictly for model evaluation. No recalibration, model selection, ensemble-weight optimization, or threshold re-estimation was performed using the test-set calibration results. The validation-derived ensemble weights and operating thresholds remained fixed throughout the analysis.

Hard Voting was excluded from the calibration intercept, calibration slope, and reliability-curve analyses because its final output is a discrete positive-vote proportion rather than a continuously estimated model-derived probability. Its Brier-type prediction error is reported separately where relevant but should not be interpreted as probability calibration of a continuous probabilistic model.

Supplementary Table S14. Disease-specific probability calibration metrics of the principal final-level ensemble methods on the held-out test set

Part A. Disease-specific Brier scores

Disease	Soft Voting	CWO	FAHP	FAHP–ΔNB
Atelectasis	0.08473	0.08167	0.08584	0.08553
Cardiomegaly	0.02569	0.02496	0.02556	0.02423
Effusion	0.08063	0.07999	0.08101	0.08085
Infiltration	0.13797	0.13737	0.13855	0.13763
Mass	0.04662	0.04494	0.04693	0.04637
Nodule	0.05776	0.05651	0.05770	0.05770
Pneumonia	0.01295	0.01259	0.01319	0.01319
Pneumothorax	0.04026	0.04014	0.04044	0.04043
Consolidation	0.04428	0.04371	0.04416	0.04416
Edema	0.02203	0.02058	0.02120	0.02051
Emphysema	0.01906	0.01838	0.01915	0.01888
Fibrosis	0.01672	0.01641	0.01685	0.01685
Pleural Thickening	0.03410	0.03380	0.03402	0.03389
Hernia	0.00138	0.00133	0.00152	0.00145

Part B. Disease-specific calibration intercept and slope

Values are presented as **calibration intercept / calibration slope**; ideal values are **0 / 1**.

Disease	Soft Voting	CWO	FAHP	FAHP–ΔNB
Atelectasis	−0.440 / 1.208	−0.306 / 1.136	−0.517 / 1.188	−0.498 / 1.189
Cardiomegaly	−1.130 / 1.140	−1.011 / 1.082	−1.148 / 1.114	−1.023 / 1.088
Effusion	−0.602 / 1.124	−0.565 / 1.107	−0.649 / 1.083	−0.641 / 1.080
Infiltration	−0.407 / 1.180	−0.355 / 1.213	−0.457 / 1.142	−0.423 / 1.140
Mass	−0.939 / 1.126	−0.934 / 1.029	−1.028 / 1.056	−0.988 / 1.064
Nodule	−0.733 / 1.290	−0.775 / 1.203	−0.889 / 1.182	−0.889 / 1.182
Pneumonia	−1.044 / 1.226	−0.979 / 1.220	−1.148 / 1.199	−1.148 / 1.199
Pneumothorax	−0.791 / 1.088	−0.816 / 1.054	−0.845 / 1.037	−0.845 / 1.036
Consolidation	−0.852 / 1.126	−0.757 / 1.169	−0.848 / 1.121	−0.848 / 1.121
Edema	−1.284 / 1.078	−1.063 / 1.129	−1.154 / 1.121	−1.092 / 1.104
Emphysema	−0.805 / 1.329	−0.781 / 1.274	−0.995 / 1.216	−0.991 / 1.185
Fibrosis	−0.466 / 1.373	−0.482 / 1.317	−0.473 / 1.381	−0.473 / 1.381
Pleural Thickening	−0.814 / 1.157	−0.890 / 1.080	−0.868 / 1.120	−0.926 / 1.065
Hernia	0.589 / 1.651	0.030 / 1.463	1.113 / 1.832	1.575 / 1.822

Part C. Macro-average Brier score across the 14 disease labels

Method	Brier score, mean ± SD
Soft Voting	0.0446 ± 0.0349
CWO	0.0437 ± 0.0347
FAHP	0.0447 ± 0.0352
FAHP–ΔNB	0.0444 ± 0.0351

Note: Brier scores were calculated from final continuous predicted probabilities on the held-out test set and are threshold-independent; lower values indicate lower overall squared probability prediction error. Part A reports disease-specific Brier scores, Part B reports disease-specific calibration intercepts and slopes, and Part C reports the unweighted mean and standard deviation of the 14 disease-specific Brier scores. The standard deviation in Part C reflects between-disease variation and should not be interpreted as seed-level or patient-level uncertainty.

Soft Voting represents equal-weight probability aggregation across the five base architectures with equal contribution of the 10 random seeds. CWO represents disease-specific validation-derived architecture weighting optimized to minimize the Brier score under non-negativity and sum-to-one constraints while retaining equal contribution of the 10 seeds. FAHP represents disease-specific validation-derived FAHP architecture weighting with equal seed aggregation, whereas FAHP–ΔNB represents the complete proposed framework incorporating disease-specific FAHP architecture weighting and validation-derived ΔNB-guided seed aggregation.

All 56 disease–method calibration models (14 diseases × 4 probability-based ensemble methods) converged successfully. Calibration coefficients were calculated for evaluation purposes only; no test-set recalibration was performed, and no hypothesis testing was conducted for calibration intercept or slope. Boldface in Parts A and C indicates the lowest Brier score.

Calibration results

The four probability-based ensemble methods showed broadly similar disease-specific calibration patterns. For most disease labels, the reliability curves lay predominantly below the identity line, indicating that mean predicted probabilities generally exceeded the corresponding observed event proportions. Consistent with this visual pattern, 52 of the 56 disease–method calibration intercepts were negative.

All calibration slopes were greater than 1.0, indicating that the predicted log-odds were generally insufficiently dispersed relative to ideal calibration. Thus, the calibration results suggest a combination of overall probability overestimation for most disease–method combinations and under-dispersion of predicted log-odds. These findings should be interpreted as descriptive calibration characteristics rather than evidence of post-hoc recalibration, because all reported probabilities remained unchanged.

CWO yielded the lowest macro-average Brier score (0.0437) and the lowest disease-specific Brier score for 12 of the 14 disease labels. FAHP–ΔNB yielded the lowest Brier score for Cardiomegaly and Edema. Nevertheless, none of the evaluated methods achieved ideal calibration across all diseases, and differences among Soft Voting, CWO, FAHP, and FAHP–ΔNB were generally modest relative to the disease-specific variation in calibration characteristics. Calibration estimates for Hernia should be interpreted cautiously because of the very small number of positive cases.

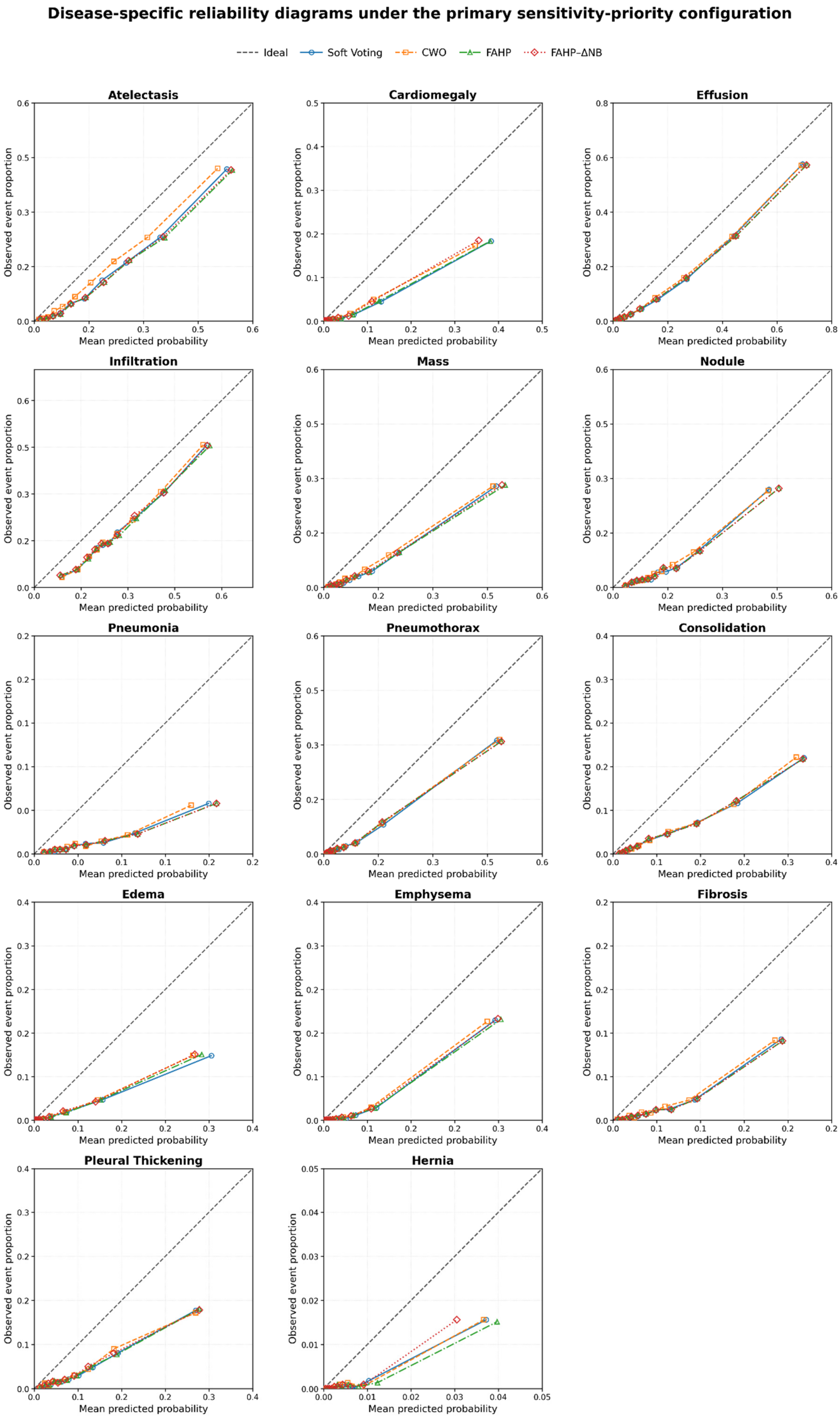

Supplementary Figure S1. Disease-specific calibration curves of the principal final-level ensemble methods under the primary sensitivity-priority configuration

Reliability curves compare the mean predicted probability with the observed event proportion using 10 quantile-based probability bins for each disease. The dashed diagonal line represents ideal calibration. Curves below the identity line indicate that the corresponding mean predicted probabilities exceed the observed event proportions. Soft Voting, Constrained Weight Optimization (CWO), FAHP, and FAHP–ΔNB are shown. Hard Voting was excluded because its final output is a discrete positive-vote proportion rather than a continuously estimated model-derived probability. Calibration curves were generated for evaluation only; no test-set recalibration was performed.

Ensemble Ablation and Conventional Weighting Comparison

To isolate the contributions of the individual ensemble components and to compare the proposed framework with a conventional validation-optimized weighting strategy, final-level performance was compared across Soft Voting, CWO, FAHP, and FAHP–ΔNB under the primary sensitivity-priority configuration.

Soft Voting served as the equal-weight probability-aggregation reference. Predicted probabilities from the five base architectures were equally averaged within each random seed, and the resulting 10 seed-level ensemble probabilities were subsequently averaged with equal seed weights.

FAHP isolated the contribution of disease-specific architecture weighting. The same validation-derived FAHP architecture weights used in the proposed framework were applied within each seed, whereas the resulting 10 seed-level FAHP probabilities were aggregated using equal seed weights. Thus, comparison of FAHP with Soft Voting evaluates the effect of replacing equal architecture weighting with disease-specific FAHP architecture weighting while leaving seed aggregation unchanged.

FAHP–ΔNB represented the complete proposed framework. It retained the same disease-specific FAHP architecture weights but replaced equal seed averaging with disease-specific ΔNet Benefit-guided seed weighting derived exclusively from the validation set. Consequently, comparison of FAHP–ΔNB with FAHP isolates the incremental effect of the ΔNB-guided seed-aggregation stage.

CWO was included as a conventional optimization-based comparator rather than as an ablation component of the proposed framework. For each disease, the 10 seed-specific probabilities were equally averaged within each architecture, after which a disease-specific architecture-weight vector was estimated exclusively from the validation set by minimizing the Brier score subject to non-negativity and sum-to-one constraints. The optimized CWO weights and corresponding validation-derived operating thresholds were fixed before evaluation on the held-out test set.

Supplementary Table S15. Component-wise ablation and conventional weighting comparison under the primary sensitivity-priority configuration

Part A. Aggregation structure of the evaluated probability-based ensemble methods

Method	Architecture-level aggregation	Seed-level aggregation	Final operating threshold
Soft Voting	Equal weighting across five architectures	Equal weighting across 10 seeds	Validation-derived Youden J
CWO	Disease-specific validation Brier-optimized architecture weights	Equal contribution of 10 seeds	Validation-derived Youden J
FAHP	Disease-specific validation-derived FAHP architecture weights	Equal weighting across 10 seeds	Validation-derived Youden J
FAHP–ΔNB	Disease-specific validation-derived FAHP architecture weights	Disease-specific validation-derived ΔNB weighting across 10 seeds	Validation-derived Youden J

Part B. Final-level macro-averaged performance across the 14 disease labels

Method	AUROC	AUPRC	Sensitivity	Specificity	Precision	F1 score	Balanced accuracy	Brier score
Soft Voting	0.8546	0.2996	0.7717	0.7825	0.1509	0.2355	0.7771	0.0446
CWO	0.8518	0.2959	0.7661	0.7840	0.1507	0.2325	0.7751	0.0437
FAHP	0.8547	0.2975	0.7773	0.7805	0.1480	0.2332	0.7789	0.0447
FAHP–ΔNB	0.8549	0.2986	0.7840	0.7768	0.1470	0.2329	0.7804	0.0444

Note: Values are unweighted macro-averages across the 14 disease labels on the held-out test set after final aggregation across the 10 random seeds. Because final predictions were obtained before performance calculation, these values represent single final-level estimates and are not reported as mean ± SD across seeds. For each probability-based method, disease-specific operating thresholds were derived exclusively from the corresponding aggregated validation probabilities using Youden's index and were applied unchanged to the held-out test set. Boldface indicates the highest value for metrics for which higher values are preferable and the lowest value for Brier score.

CWO was designed to minimize validation-set Brier score and therefore represents an optimization-based probability-weighting comparator rather than a component of the proposed FAHP–ΔNB framework. FAHP and FAHP–ΔNB used the primary sensitivity-priority FAHP preference configuration.

Ablation Results

Replacing equal architecture weighting in Soft Voting with disease-specific FAHP weighting while retaining equal seed aggregation increased macro-average sensitivity from 0.7717 to 0.7773 and balanced accuracy from 0.7771 to 0.7789. AUROC remained essentially unchanged (0.8546 versus 0.8547), whereas AUPRC, precision, F1 score, and Brier score showed small unfavorable changes. These results indicate that the FAHP architecture-weighting stage primarily altered the operating trade-off toward the prespecified sensitivity-oriented preference rather than uniformly improving every performance endpoint.

Adding validation-derived ΔNB-guided seed weighting to FAHP further increased sensitivity from 0.7773 to 0.7840 and balanced accuracy from 0.7789 to 0.7804. AUROC increased from 0.8547 to 0.8549, AUPRC from 0.2975 to 0.2986, and Brier score decreased from 0.0447 to 0.0444. These improvements were accompanied by small reductions in specificity, precision, and F1 score. The incremental comparison therefore suggests that ΔNB-guided seed aggregation provided additional sensitivity and balanced-accuracy gains beyond FAHP architecture weighting, while preserving the expected operating trade-off of the primary sensitivity-priority configuration.

CWO achieved the lowest macro-average Brier score (0.0437), consistent with its direct validation objective of Brier-score minimization. However, its AUROC (0.8518), AUPRC (0.2959), and balanced accuracy (0.7751) were lower than those of the complete FAHP–ΔNB framework. Thus, direct probability-error optimization and the proposed preference- and utility-guided ensemble addressed different optimization objectives and produced different performance trade-offs.

Taken together, the ablation results indicate that the two stages of the proposed framework had distinguishable roles: disease-specific FAHP weighting modified architecture contributions according to the predefined diagnostic preference structure, whereas ΔNB-guided seed aggregation provided an additional validation-derived utility-based adjustment across independently trained ensemble members. The results do not indicate uniform superiority of any single weighting strategy across all endpoints; rather, they demonstrate the intended trade-off between sensitivity-oriented diagnostic performance and probability prediction accuracy.

Hyperparameter Sensitivity Analysis

Two weighting exponents were evaluated to assess whether the performance of the proposed ensemble was materially dependent on the prespecified hyperparameter settings.

The first parameter, γ, controls the contrast among disease-specific FAHP architecture scores before normalization. For disease d and architecture k, the validation-derived criterion-weighted architecture score was defined as

$$S_{d,k} = \sum_{c \in C} w_c \, Z_{d,k,c},$$

where $Z_{d,k,c}$ denotes the normalized validation performance of architecture k for criterion c, w_c denotes the corresponding FAHP criterion weight, and C comprises sensitivity, specificity, and precision.

The disease-specific architecture weight was then calculated as

$$\alpha_{d,k}(\gamma) = \frac{S_{d,k}^{\gamma}}{\sum_{j=1}^{K} S_{d,j}^{\gamma}},$$

where $K = 5$ denotes the number of base architectures. Accordingly, $\gamma = 1$ corresponds to direct normalization of the criterion-weighted architecture scores, whereas larger values of γ progressively increase the contrast between higher- and lower-scoring architectures.

The second parameter, γ_{NB}, controls the relative emphasis assigned to positive validation-derived ΔNet Benefit values during aggregation of the 10 seed-level FAHP predictions. For disease d and seed s, the unnormalized seed weight was defined as

$$u_{d,s}(\gamma_{\mathrm{NB}}) = \begin{cases} \epsilon + \left(\Delta NB_{d,s}\right)^{\gamma_{\mathrm{NB}}}, & \Delta NB_{d,s} > 0, \\ \epsilon, & \Delta NB_{d,s} \leq 0 \text{ or is non-finite.} \end{cases}$$

where $\epsilon = 10^{-6}$ is a small baseline contribution retained for seeds with non-positive or non-finite ΔNet Benefit.

The corresponding normalized seed aggregation weight was

$$w_{d,s}^{\Delta NB}(\gamma_{\mathrm{NB}}) = \frac{u_{d,s}(\gamma_{\mathrm{NB}})}{\sum_{r=1}^{S} u_{d,r}(\gamma_{\mathrm{NB}})}$$

where $S = 10$ denotes the number of random seeds. Thus, increasing γ_{NB} increases the relative emphasis on seeds with larger positive validation-derived ΔNet Benefit, whereas seeds with non-positive or non-finite values retain only the predefined baseline contribution. When all seed-specific ΔNet Benefit values for a disease were non-positive or non-finite, all seeds received the same baseline contribution and therefore had equal normalized aggregation weights of $1/S = 0.1$.

The prespecified primary configuration was $\gamma = 2.0$ and $\gamma_{\mathrm{NB}} = 1.0$.

Sensitivity analyses were conducted using a one-factor-at-a-time design. For the FAHP architecture-weighting analysis, γ was varied across 1.0, 1.5, 2.0, and 3.0 while γ_{NB} was fixed at 1.0. For the ΔNet Benefit seed-weighting analysis, γ_{NB} was varied across 0.5, 1.0, and 2.0 while γ was fixed at 2.0.

The same data split, trained base-model predictions, sensitivity-priority FAHP preference configuration, and validation-only analysis pipeline were used for all sensitivity settings. For each parameter setting, the parameter-dependent architecture weights, seed aggregation weights, and corresponding operating thresholds were recalculated using validation data and subsequently locked before application to the held-out test set. Test-set results were used solely to assess robustness and were not used to re-select the prespecified primary hyperparameter values.

Across the tested values of γ, macro-average AUROC ranged from 0.8540 to 0.8553, AUPRC from 0.2959 to 0.3010, balanced accuracy from 0.7789 to 0.7804, and Brier score from 0.0444 to 0.0445. The total ranges were therefore only 0.0013 for AUROC and 0.0015 for balanced accuracy.

Varying γ_{NB} produced even smaller changes in discrimination. Across $\gamma_{\mathrm{NB}} =$ 0.5 to 2.0 , macro-average AUROC ranged from 0.8548 to 0.8550, AUPRC from 0.2985 to 0.2988, balanced accuracy from 0.7792 to 0.7804, and Brier score from 0.0443 to

0.0445.

These results indicate that the principal findings were stable across reasonable perturbations of both weighting exponents. The prespecified primary values, $\gamma = 2.0$ and $\gamma_{\mathrm{NB}} = 1.0$, were therefore retained for the primary analysis rather than re-selecting hyperparameters on the basis of held-out test-set performance.

Supplementary Table S16. Sensitivity analysis of the FAHP model-weighting exponent γ and the ΔNet Benefit seed-weighting exponent γ_{NB}

Part A. Sensitivity to the FAHP model-weighting exponent γ, with $\gamma_{\mathrm{NB}} = 1.0$

FAHP γ	γNB	AUROC	AUPRC	Sensitivity	Specificity	Precision	F1 score	Balanced accuracy	Brier score
1.0	1.0	0.8553	0.3010	0.7884	0.7710	0.1480	0.2326	0.7797	0.0445
1.5	1.0	0.8552	0.2999	0.7824	0.7779	0.1456	0.2295	0.7801	0.0445
**2.0†**	**1.0**	**0.8549**	**0.2986**	**0.7840**	**0.7768**	**0.1470**	**0.2329**	**0.7804**	**0.0444**
3.0	1.0	0.8540	0.2959	0.7856	0.7721	0.1457	0.2292	0.7789	0.0444

Part B. Sensitivity to the ΔNet Benefit seed-weighting exponent γ_{NB}, with $\gamma = 2.0$

FAHP γ	γNB	AUROC	AUPRC	Sensitivity	Specificity	Precision	F1 score	Balanced accuracy	Brier score
2.0	0.5	0.8548	0.2985	0.7797	0.7787	0.1466	0.2311	0.7792	0.0445
**2.0**	**1.0†**	**0.8549**	**0.2986**	**0.7840**	**0.7768**	**0.1470**	**0.2329**	**0.7804**	**0.0444**
2.0	2.0	0.8550	0.2988	0.7860	0.7737	0.1450	0.2287	0.7799	0.0443

Note: † Prespecified primary configuration. Values are unweighted macro-averages across the 14 disease labels on the held-out test set after final 10-seed aggregation. Sensitivity analyses were performed using the same base-model predictions and sensitivity-priority FAHP preference configuration. For each parameter setting, parameter-dependent ensemble weights and operating thresholds were derived from the validation set and locked before test-set evaluation. These analyses were conducted solely to assess robustness and were not used to re-select hyperparameters based on test-set performance. Lower Brier scores indicate better probability accuracy.

Supplementary Table S17. Final-level macro-averaged performance of FAHP–ΔNB under alternative FAHP preference configurations

Configuration	AUROC	AUPRC	Sensitivity	Specificity	Precision	F1 score	Balanced accuracy	Brier score
Sensitivity-priority	0.8549	0.2986	**0.7840**	0.7768	0.1470	0.2329	**0.7804**	0.0444
Specificity-priority	0.8550	0.3008	0.7594	**0.7973**	**0.1542**	**0.2383**	0.7783	**0.0441**
Balanced	**0.8559**	**0.3020**	0.7696	0.7909	0.1526	0.2381	0.7803	0.0442

Note: Values represent final-level performance after disease-specific FAHP model weighting and ΔNB-guided aggregation across 10 random seeds. Because seed-level predictions were aggregated before final evaluation, no across-seed standard deviation is reported for these final-level metrics. Boldface indicates the highest value among the three configurations for each metric; for Brier score, the lowest value is highlighted.

Supplementary Table S18. Disease-specific final-level operating characteristics of FAHP–ΔNB under alternative preference configurations

**Disease**	**Sensitivity-priority**			**Balanced**			**Specificity-priority**		
	Sens.	Spec.	BA	Sens.	Spec.	BA	Sens.	Spec.	BA
Atelectasis	0.781	**0.732**	0.756	**0.800**	0.713	**0.757**	0.795	0.719	**0.757**
Cardiomegaly	**0.881**	0.823	**0.852**	0.863	0.836	0.849	0.845	**0.844**	0.845
Effusion	**0.866**	0.759	0.812	0.824	**0.803**	**0.814**	0.826	0.802	**0.814**
Infiltration	**0.626**	0.689	0.658	0.591	0.728	**0.660**	0.582	**0.737**	0.659
Mass	0.770	0.813	0.791	**0.778**	0.808	**0.793**	0.713	**0.852**	0.783
Nodule	**0.705**	0.741	0.723	0.659	0.796	**0.728**	0.604	**0.839**	0.722
Pneumonia	**0.657**	0.759	**0.708**	0.587	0.812	0.700	0.579	**0.816**	0.697
Pneumothorax	**0.812**	0.838	**0.825**	**0.812**	0.837	0.824	0.805	**0.845**	**0.825**
Consolidation	0.812	0.678	**0.745**	0.803	**0.684**	0.743	**0.819**	0.666	0.743
Edema	0.881	0.787	**0.834**	**0.889**	0.776	0.832	0.872	**0.789**	0.830
Emphysema	0.847	0.901	**0.874**	0.845	**0.904**	**0.874**	**0.912**	0.828	0.870
Fibrosis	**0.823**	0.685	0.754	**0.823**	0.706	**0.764**	0.818	**0.710**	**0.764**
Pleural Thickening	**0.683**	0.771	**0.727**	0.668	0.783	0.725	0.580	**0.848**	0.714
Hernia	0.833	**0.897**	0.865	0.833	0.887	0.860	**0.881**	0.868	**0.874**

Note: Sens., sensitivity; Spec., specificity; BA, balanced accuracy. Values represent disease-specific final-level performance after FAHP model weighting and ΔNB-guided aggregation across 10 random seeds. Disease-specific final thresholds were derived from the corresponding aggregated validation probabilities using Youden's index and were applied unchanged to the held-out test set. Boldface indicates the highest displayed value among the three preference configurations within each disease and metric; ties at the reported precision are highlighted for all tied configurations.

Supplementary S8. Spatial Explainability Analysis

Grad-CAM-Based Ensemble Visualization

To provide a qualitative assessment of the spatial patterns associated with the ensemble predictions, gradient-weighted class activation mapping (Grad-CAM) was applied to representative cases from the held-out test set. This analysis was performed as a supplementary explainability assessment and did not contribute to model training, model selection, threshold derivation, ensemble weighting, or quantitative performance evaluation.

Grad-CAM maps were generated separately for the target disease class from each of the five base architectures—DenseNet121, ViT, SwinV2, DenseNet + ViT, and DenseNet + SwinV2—using the corresponding trained checkpoints from the 10 random seeds. Architecture-appropriate late feature layers were used as the Grad-CAM target layers. For transformer-based architectures, the intermediate token representations were reshaped into two-dimensional spatial feature maps before calculation of the class-specific activation map. Each individual activation map was normalized before subsequent aggregation.

Because the evaluated ensemble methods operate on predictions from multiple independently trained models rather than through a single differentiable end-to-end ensemble network, ensemble-level Grad-CAM maps were constructed by aggregating the corresponding base-model activation maps according to the aggregation structure of each method. These maps should therefore be interpreted as visualization-level spatial summaries of component-model Grad-CAM patterns associated with the target-class predictions, rather than as intrinsic saliency maps generated directly by a single ensemble network.

Ensemble-Level Grad-CAM Aggregation

For **Soft Voting**, activation maps were first equally aggregated across the 10 random seeds for each architecture and were subsequently averaged equally across the five base architectures. This corresponds to the equal-weight architecture and seed aggregation used by the final Soft Voting probability ensemble.

For **FAHP**, Grad-CAM maps were first equally aggregated across the 10 seeds within each architecture. The resulting architecture-level maps were then combined using the same disease-specific FAHP architecture weights derived exclusively from validation-set sensitivity, specificity, and precision. Thus, this visualization isolates the spatial effect of disease-specific FAHP architecture weighting while retaining equal contribution of the random seeds.

For **FAHP–ΔNB**, the same disease-specific FAHP architecture weights were retained, while the seed-level Grad-CAM maps were aggregated using the disease-specific validation-derived ΔNet Benefit (ΔNB) seed weights used in the complete proposed framework. Accordingly, differences between the FAHP and FAHP–ΔNB maps reflect the additional influence of ΔNB-guided seed aggregation.

For **Constrained Weight Optimization (CWO)**, seed-level maps were equally aggregated within each architecture and the resulting architecture-level maps were combined using the disease-specific CWO weights estimated on the validation set by Brier-score minimization under non-negativity and sum-to-one constraints. This visualization therefore reflects the architecture-weighting structure of the conventional optimization-based comparator while retaining equal seed contribution.

Hard Voting required a separate visualization strategy because majority voting is non-differentiable and does not itself generate a continuous class activation map. For each random seed, base architectures were classified as positive or negative using their corresponding disease- and seed-specific validation-derived Youden thresholds. For seed-level Hard Voting decisions classified as positive, the Grad-CAM maps of the positive-voting base models were averaged to obtain one seed-level spatial map. The resulting maps from positive Hard Voting seeds were subsequently averaged to construct the final Hard Voting visualization. Consequently, the Hard Voting map represents a visualization-level summary of spatial evidence from models that contributed positive votes and should not be interpreted as an intrinsic Grad-CAM of the majority-voting operation itself.

For visualization only, the aggregated activation maps were normalized to the range 0–1 and Gaussian smoothing was applied before overlaying the heatmaps on the corresponding chest radiographs. These display operations did not modify model

probabilities, operating thresholds, ensemble weights, or binary predictions.
